# GRADE‐Based Clinical Practice Guidelines for Emergency Department Delirium Risk Stratification, Screening, and Brain Imaging in Older Patients With Suspected Delirium

**DOI:** 10.1111/acem.70167

**Published:** 2025-10-27

**Authors:** Sangil Lee, Danya Khoujah, Debra Eagles, Maura Kennedy, Alexander X. Lo, Christian H. Nickel, Glenn Arendts, Luna Ragsdale, Justine Seidenfeld, Kerstin de Wit, Ines Luciani‐Mcgillivray, Christopher R. Carpenter, Shan W. Liu, the Geriatric Emergency Department Delirium Guidelines 2.0 Writing Team

**Affiliations:** ^1^ University of Iowa Iowa City Iowa USA; ^2^ Department of Emergency Medicine Tampa AdventHealth Tampa Florida USA; ^3^ Department of Emergency Medicine University of Maryland Baltimore Maryland USA; ^4^ Department of Emergency Medicine, School of Epidemiology and Public Health University of Ottawa Ottawa Ontario Canada; ^5^ Ottawa Hospital Research Institute Ottawa Ontario Canada; ^6^ Harvard Medical School, Massachusetts General Hospital Boston Massachusetts USA; ^7^ Department of Emergency Medicine Northwestern University School of Medicine Chicago Illinois USA; ^8^ Center for Health Services and Outcomes Research Northwestern University School of Medicine Chicago Illinois USA; ^9^ Emergency Department University Hospital Basel, University of Basel Basel Switzerland; ^10^ Medical School, University of Western Australia Perth Western Australia Australia; ^11^ Durham Veterans Affairs Health Care System Durham North Carolina USA; ^12^ Center of Innovation to Accelerate Discovery and Practice Transformation Durham Veterans Affairs Health Care System Durham North Carolina USA; ^13^ Department of Emergency Medicine University of Queens Kingston Ontario Canada; ^14^ Division of Emergency Medicine Department of Medicine, McMaster University Ontario Canada; ^15^ Department of Emergency Medicine Massachusetts General Hospital Boston Massachusetts USA; ^16^ Mayo Clinic Rochester Minnesota USA; ^17^ Harvard Medical School Boston Massachusetts USA

## Abstract

**Objectives:**

This portion of the Geriatric Emergency Department (GED) Guidelines 2.0 focuses on delirium in the emergency department (ED).

**Methods:**

A multidisciplinary group applied the Grading of Recommendations Assessment, Development, and Evaluation (GRADE) approach to assess the certainty of evidence and develop recommendations related to older ED patients with possible delirium.

**Results:**

The GED Guidelines 2.0 Delirium Work Group derived six evidence‐based recommendations for risk stratification, diagnosis, and brain imaging. To reduce universal screening, the Delirium Risk Score may be used to identify older adults at low risk for delirium, though the evidence certainty is very low. In adults over 65 admitted to ED observation units, Zucchelli's risk assessment tool (threshold ≥ 4) may stratify delirium risk, also with very low certainty. For adults over 75, the REDEEM Score may be used to identify low‐ or high‐risk individuals, again with very low certainty. For diagnosis, 4AT, bCAM, CAM‐ICU, mCAM, AMT‐4, or RASS may be used to rule delirium in or out, based on very low certainty. The Delirium Triage Screen (DTS) may be used to rule out, but not to rule in, delirium, also with very low certainty. For diagnostic imaging, there is very low certainty of evidence to recommend for or against obtaining a head CT as part of the evaluation for older ED patients with delirium. All recommendations are conditional, reflecting very low certainty of evidence due to the lack of high‐quality ED‐based studies and comparative effectiveness research.

**Conclusion:**

Rigorous ED‐based research is needed to strengthen evidence and guide delirium care for older adults in geriatric emergency medicine.

## 1 Introduction

In 2014, the Geriatric Emergency Department (GED) Guidelines 1.0 were published by multiple emergency medicine (EM) and geriatrics professional organizations [1]. The GED Guidelines 1.0 were developed in response to the growth in self‐labeled “geriatric emergency departments”, which were significantly variable in the absence of structure [1, 2]. The GED Guidelines 1.0 provided consensus‐based actionable recommendations and quality metrics for protocol development for geriatric syndromes and the requisite infrastructure to promote aged‐friendly care [1]. Subsequently, the American College of Emergency Physicians (ACEP) began to accredit hospitals when their EDs demonstrated adherence to these guidelines, [3, 4] with over 500 accredited EDs worldwide as of February 2025 [5, 6].

Since the GED Guidelines 1.0 were published, the process of creating clinical practice guidelines has evolved. The Grading of Recommendation, Assessment, Development, and Evaluation (GRADE) approach has been internationally adopted by over 100 guideline‐creating organizations, [7] including the Society for Academic Emergency Medicine (SAEM) Guidelines for Reasonable and Appropriate Care in the Emergency Department (GRACE) [8, 9, 10, 11]. GRADE provides a rigorous and transparent approach to assessing the quality and applicability of evidence for a focused question while also explicitly incorporating factors such as the quantitative balance of anticipated benefits, harms, costs, feasibility, health equity, and alliance with stakeholders' values from direct and indirect evidence [12, 13], in addition to identifying knowledge gaps requiring research as well as implementation approaches to accelerate the uptake of the recommendations.

In 2020, the GED Guidelines 2.0 Writing Team was organized to update the original GED Guidelines 1.0, focusing on older adults, selecting the GRADE methodology [14, 15], and electing to publish separate guideline documents for each geriatric syndrome (delirium, dementia, falls, medication safety, elder abuse, and frailty). This document provides recommendations for risk stratification, assessment, and brain imaging.

As outlined in Table 1, delirium is an acute brain dysfunction that commonly affects older adults—often manifesting as confusion, altered level of consciousness, inattention, and perceptual disturbances [17]. Older adults often present to the ED with altered mental status; approximately 6%–38% of older patients who visit the ED have delirium [18, 19, 20, 21]. The wide range of observed ED delirium is a consequence of differing criterion standards used across studies to define the presence or absence of delirium, as well as variable distinctions between and inclusion of incident and prevalent delirium [20, 22]. Delirium increases mortality rates and leads to functional decline at an estimated cost of $152 billion US dollars to the healthcare system annually [17, 23]. Nearly 15 years ago, the American Geriatrics Society (AGS) and SAEM recommended prioritizing delirium care, including delirium screening and preventative interventions [24, 25, 26, 27, 28]. Seven Cochrane reviews have been published since 2012 synthesizing delirium interventions across a variety of patient populations and clinical settings. However, none included studies from the ED, nor identified a consistent strategy or intervention to prevent incident delirium or treat prevalent delirium [20, 29]. The American Delirium Society houses a list of the existing guidelines for delirium care in the diverse clinical discipline, but its outreach has not involved the ED as of 2024 [30].

**TABLE 1:** Diagnostic criteria for delirium [16].

The DSM‐5 TR (Diagnostic and Statistical Manual of Mental Disorders, Fifth Edition, Text Revision) defines delirium as a disturbance in attention and awareness that develops over a short period of time and represents an acute change from baseline [16]. It is characterized by the following diagnostic criteria
1. A disturbance in attention (i.e., reduced ability to direct, focus, sustain, and shift attention) accompanied by reduced awareness of the environment.
2. Develops over a short period of time (usually hours to a few days), represents an acute change from baseline, and tends to fluctuate in severity during the course of the day.
3. An additional disturbance in cognition (e.g., memory deficit, disorientation, language, visuospatial ability, or perception).
4. The disturbances in attention and cognition are not better explained by another preexisting, established, or evolving neurocognitive disorder and do not occur in the context of a severely reduced level of arousal, such as coma.
5. There is evidence from the history, physical examination, or laboratory findings that the disturbance is a direct physiological consequence of another medical condition, substance intoxication or withdrawal (e.g., due to a drug of abuse or a medication), exposure to a toxin, or is due to multiple etiologies.

## 2 Scope and Purpose

**FIGURE 1:**
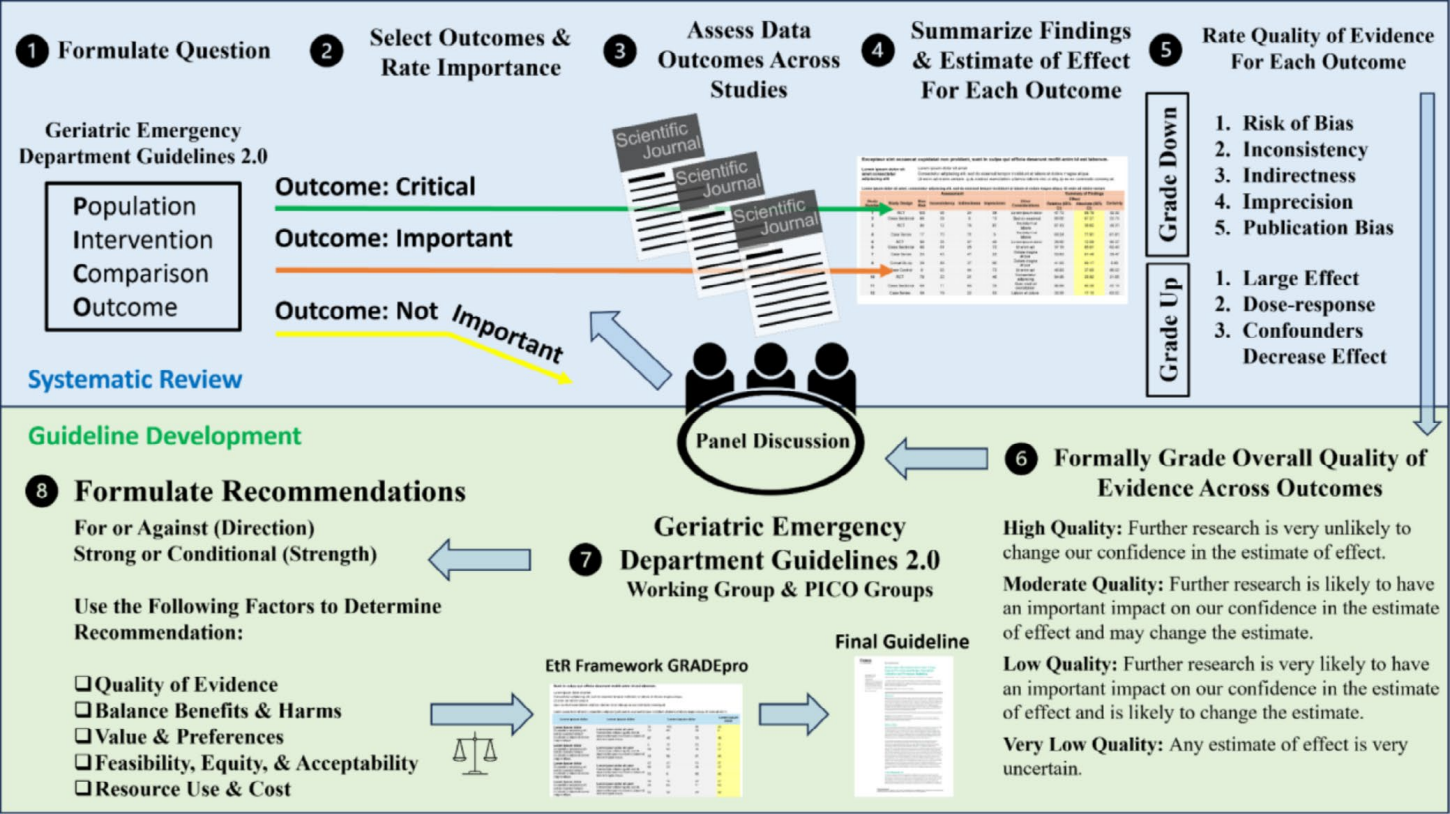
GRADE process adapted for the geriatric ED guidelines [31].

The GED Delirium Guidelines 2.0 evaluate the quality of direct and indirect evidence to create actionable recommendations on the following questions: (1) Which ED older adults should be further assessed for delirium? (2) Which diagnostic tests should be used to identify ED delirium?, and (3) Should acutely confused patients have a head computed tomography (CT) as part of their evaluation for delirium etiology? (Supplements S1–S3). The purpose of the GED Guidelines 2.0 is to provide a contemporaneous, transparent, evidence‐based, patient‐centred approach for clinicians in their evaluation and management of older adults in the ED based on GRADE methodology. These GED Guidelines 2.0 are designed for application in the United States and other countries with comparable healthcare resources (Figure 1).

## 3 Methods

### 3.1 Group Composition

The GED Guideline 2.0 Delirium Work Group included emergency physicians and nurses from demographically and geographically diverse academic and community facilities in the U.S., Canada, Australia, United Kingdom, Ireland, and Switzerland, listed in Supplement S1. Group members collectively possess content and health outcomes research experience in delirium, clinical practice guideline development, systematic reviews, brain imaging, and geriatric emergency medicine, and are not part of a specific organization's clinical practice guidelines' team. Two care partner representatives from the authors' network participated in reviewing these guidelines; one reviewed all of these recommendations, and another participated in the development of the brain imaging recommendation. The GED Guidelines 2.0 were supported in part by the John A. Hartford Foundation.

### 3.2 Group Interaction and Processes

From January 2021 until February 2024, the delirium work group met virtually monthly, either as the entire work group or as smaller subgroups, to develop the questions [32]. The smaller subgroups subsequently conducted systematic reviews to gather evidence and synthesize the findings to formulate recommendations [22, 33, 34]. A draft of the GED Guidelines Delirium document was distributed for external stakeholder review in February 2025, including the SAEM, AGS, ACEP, American Delirium Society, Canadian Association of Emergency Physicians (CAEP), European Society of Emergency Medicine (EUSEM), American Academy of Emergency Medicine (AAEM), American College of Osteopathic Emergency Physicians, Australasian College of Emergency Medicine, and Emergency Nurses Association (ENA) for a 6‐week review period. Once these guidelines are published, we will seek endorsements from these professional societies.

### 3.3 Training

The methodologists (CRC, SL, and SWL) received GRADE training at the GRADE conference (https://gradeconf.org/) and the entire GED Guidelines 2.0 Delirium Work Group reviewed the GRACE online videos describing the GRADE rationale and approach [14].

### 3.4 Declaration and Management of Competing Interests

All delirium work group members disclosed potential conflicts of interest as per GRADE recommendations [35, 36, 37]. These disclosures were adjudicated by impartial GRADE experts from the SAEM GRACE team. These potential conflicts are listed on pages 21–22, as well as at the beginning of each individual EtD for the corresponding workgroup. The final GEDG delirium document was reviewed by a GRADE expert using the AGREE‐II assessment tool [38] for a trustworthy clinical practice guideline, which is included as Supplement S4.

### 3.5 Definitions of the Intended Patient Population

The GED Guidelines 2.0 are relevant to geriatric patients presenting to the ED, defined as 65 years or older. This cutoff was arbitrarily chosen, recognizing that 55 or 75 are sometimes chosen as the age threshold in delirium research, without a clear rationale [39]. In this document, we defined those who presented to the ED with delirium as having *prevalent delirium*, while those who developed delirium after ED arrival as having *incident delirium* [32, 34].

### 3.6 Selection of Questions

Question proposal and selection was performed by the entire GED Guideline 2.0 Group, which included the delirium work group and members from other work groups, such as medication safety, falls, and dementia. These academic and clinician experts focused on questions relevant to the practicing clinicians, incorporating lessons learned from the growth of EM research and practical experience via geriatric emergency departments. Candidate questions were submitted using the standard PICO format (Population, Intervention, Comparator, and Outcome) [32, 40], addressing different aspects of delirium in the ED, including diagnosis, screening, evaluation, management, and prevention. Candidate questions were then rank‐ordered using an online survey form [41]. The delirium work group voted in favor of 3 areas—identifying, screening, and evaluation of causes of delirium (See Supplement S5). The delirium work group then divided into three smaller subgroups to refine their PICO questions. Each subgroup selected outcomes of interest judged to be most important by the subgroup itself. These questions were then further refined by the GEDG PICO approval group, which consisted of geriatric EM researchers with extensive systematic review experience (CRC, GA, CG, SWL, LS, MK, LW, and TS).

### 3.7 Evidence Synthesis and Development of Clinical Recommendations

Each delirium subgroup published a systematic review (and where appropriate, a meta‐analysis) of direct evidence for each PICO question [22, 33, 42]. Next, each delirium subgroup created Evidence‐to‐Decision (EtD) Framework documents outlining the various GRADE judgments and justifications (Appendices S1–S3), which were then circulated among the entire delirium work groups between December 2024 and February 2025 for review, discussion, edits, additional pertinent citations, and commentary.

### 3.8 Certainty of Evidence, Strength of Recommendations, and Indirect Evidence

The certainty of evidence focused on evidence from the systematic reviews of direct evidence, but also considered indirect evidence identified as part of the EtD Framework effort [43, 44, 45, 46, 47, 48, 49, 50, 51, 52, 53, 54, 55, 56, 57, 58, 59, 60, 61, 62]. Eight criteria were considered, including risk of bias (as it pertains to the methodology of included studies), inconsistency (among included studies), indirectness (evidence from non‐ED populations), imprecision (i.e., wide confidence intervals), and other considerations [43, 44, 45, 46, 47, 48, 49, 50, 51, 52, 53, 54, 55, 56, 57, 58, 59, 60, 61, 62]. Additional considerations such as benefits, harms, costs, feasibility, effect on equity, and congruence with stakeholders' values were incorporated in the EtD frameworks. Evidence is categorized into high, moderate, low, or very low quality and synthesized into summary tables [49, 63]. Evidence was considered “indirect” if any element of the published research used to inform a decision differs from the question, and this indirectness downgraded the certainty of evidence, limiting the strength of the resulting recommendation(s) [13, 45]. Recommendations were worded following the GRADE writing style; FOR/AGAINST/EITHER were used to indicate the direction of the recommendation, and “strong” versus “conditional” to indicate the strength of the recommendation [14, 15], with transparent reporting of the strength of evidence for that recommendation [59, 60, 61, 62]. “For” indicates that the evidence supports the intervention, “against” indicates that the evidence opposes the intervention, and “either” indicates that the evidence does not support nor oppose the intervention.

**FIGURE 2:**
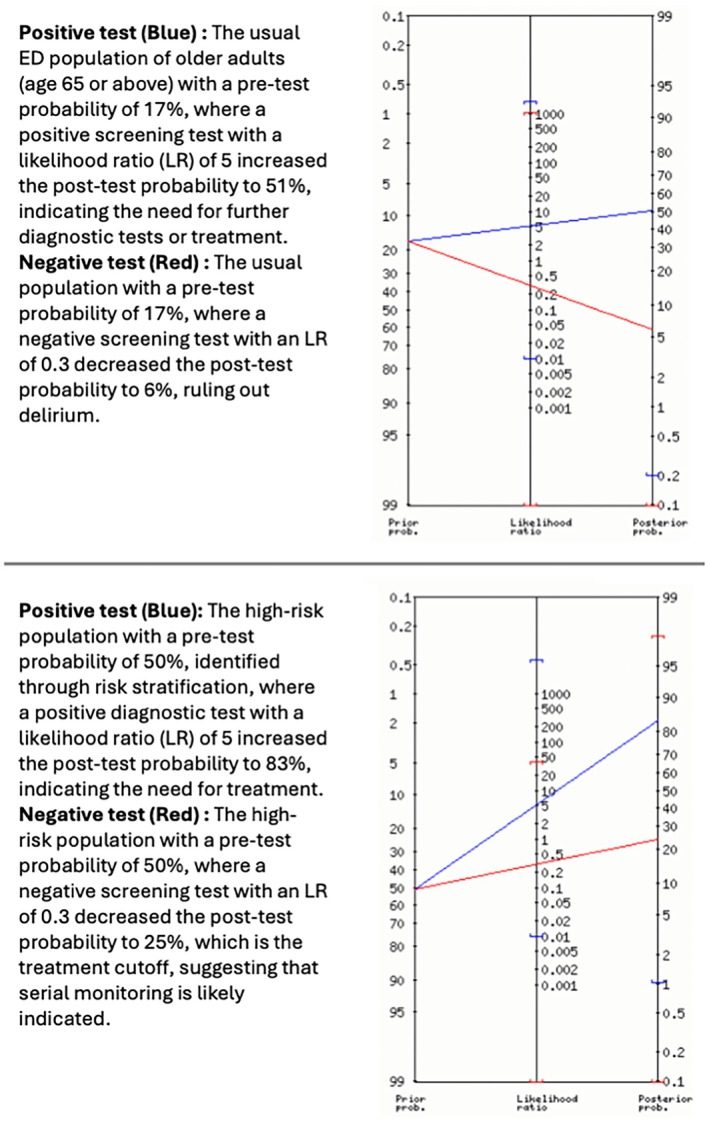
Pre‐test probability, LRs, and post‐test probability using the nomogram [68].

Two of the three PICO questions investigated were related to the use of risk stratification for screening or diagnostic tools to determine the presence or absence of delirium. Likelihood ratios (LRs) of > 4.0 and < 0.4 were considered strong enough associations to support the use of the tool given the large effect size as described by Rosenthal et al. [64] The lack of progress in delirium risk, detection, and mitigation in the ED drove us not to use the conventional cutoff of the LR+ > 10 and LR− < 0.1 [65, 66], as they would likely exclude every prognostic and diagnostic delirium instrument from the GED 2.0 Guidelines. This decision is not unique to the GED 2.0 guidelines and has been utilized by other groups including the GRACE guidelines in the past [67]. Several examples for the pre‐test probability, LR, and post‐test probability and treatment consideration appear in Figure 2.

For comparing the performance of clinical tools, we chose to focus on the point estimate rather than the 95% confidence intervals (CI) as the point estimate provides a summary measure of effect size across studies and simplifies comparisons. Precision and accuracy (calibration and discrimination) were assessed separately during the EtD framework synthesis and deliberations.

**FIGURE 3:**
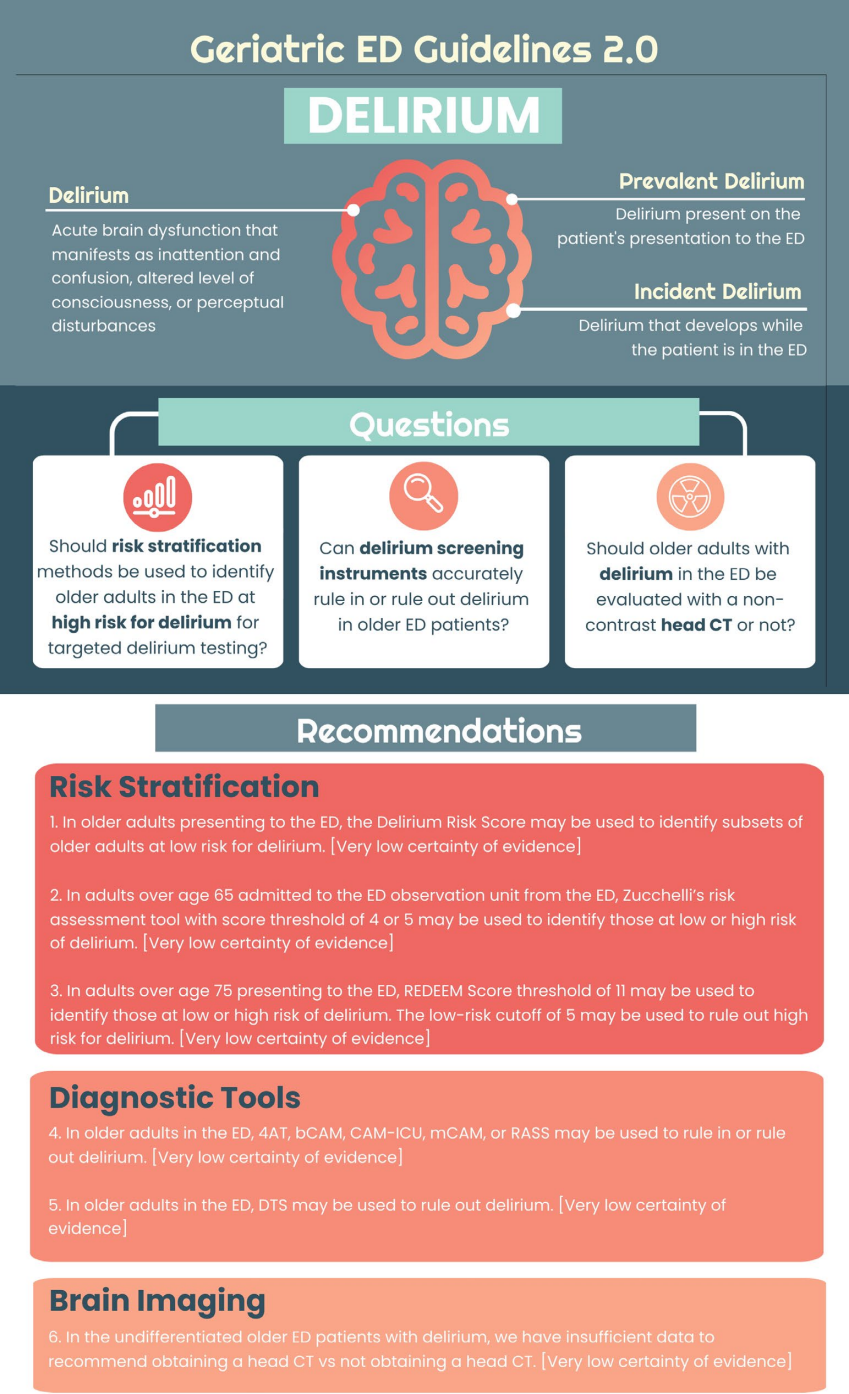
Infographic abstract.

**FIGURE 4:**
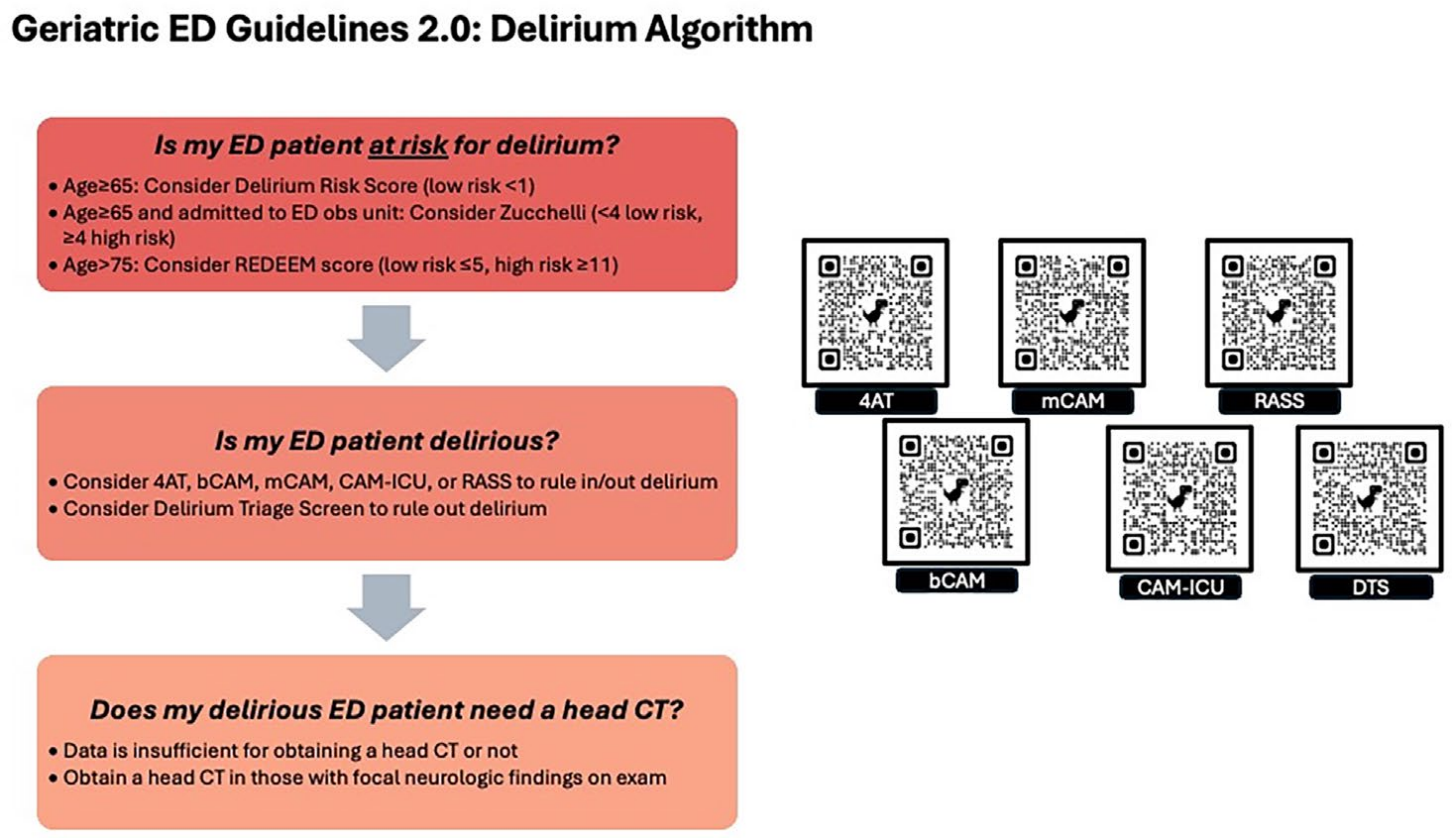
Geriatric ED Guidelines 2.0: Delirium Algorithm. This step‐wise evidence‐based flowchart outlines a systematic approach to evaluating patients for delirium, beginning with risk factor assessment and stratification. The pathway progresses through initial diagnostic tools, followed by whether imaging is recommended. QR codes embedded in the figure link to online calculators for most of the mentioned tools.

Figure 3 provides an overall summary of our PICO questions and recommendations. Figure 4 shows the recommended Geriatric ED Guidelines delirium algorithm as well as QR codes to various delirium tools.

#### 3.8.1 Question 1: Delirium Risk Stratification

*Can risk stratification methods identify a subset of older adults in the ED at high risk for delirium for targeted delirium testing?*

[Evidence to decision framework in Appendix S1].

Recommendation #1: In older adults presenting to the ED, the Delirium Risk Score may be used to identify subsets of older adults at low risk for delirium (Conditional recommendation, FOR) [Very low certainty of evidence].

Recommendation #2: In adults over age 65 admitted to the ED observation unit from the ED, Zucchelli's risk assessment tool with a score threshold of 4 may be used to identify those at low or high risk of delirium (Conditional recommendation, FOR) [Very low certainty of evidence].

Recommendation #3: In adults over age 75 presenting to the ED, a REDEEM Score threshold of 11 may be used to identify those at low or high risk of delirium. The low‐risk cutoff of 5 may be used to rule out high risk for delirium (Conditional recommendation, FOR) [Very low certainty of evidence].

##### 3.8.1.1 Summary of Evidence

Delirium is often underdiagnosed, and routine screening for all older adults is resource‐intensive [69]. Furthermore, structured approaches to screen *all* older adults in ED settings for prevalent (pre‐ED) or incident delirium do not consistently increase the frequency with which delirium is recognized [70]. Accurately and reliably identifying older ED adults at high risk for prevalent (pre‐ED) delirium might increase the yield of the diagnostic process while simultaneously minimizing the burden on ED resources. Risk factors associated with increased likelihood of delirium include dementia, cognitive impairment, functional dependence, sensory impairment, and drug burden [33]. Several risk stratification tools are featured here as they met the threshold for the LRs. The Delirium Risk Score may be used to identify subsets of older adults at low risk for delirium (LR− of 0.08), but not to identify high‐risk individuals (LR+ 1.9) [71]. In adults over age 65 admitted to the ED observation unit, Zucchelli et al. [72] evaluated various cutoffs for identifying delirium. When using the cutoff of 4 or 5, the risk assessment tool may be used to identify either high‐risk or low‐risk older adults (LR+ 4.6, LR− 0.26, LR+ 8.5, LR− 0.30). In adults over age 75 presenting to the ED, the REDEEM Score threshold of 11 may be used to identify those at low and high risk of delirium (LR+ 6.3, LR− 0.18) [73]. The Kennedy Risk Prediction Rule [19] should not be used to identify those at low or high risk of delirium. All these are conditional recommendations due to the very low certainty of evidence because of the evidence from single center studies and, in most cases, the absence of separate validation research. These risk stratification tools are summarized in Table 2.

**TABLE 2:** Delirium risk stratification tools [71, 72, 73].

Tool	Delirium risk score	Zucchelli	REDEEM score
Elements	Dementia (+1) Functional dependence (Katz Activities of Daily Living ≤ 4) (+1) Hearing impairment (+1)	Age ≥ 75 (+2) Dementia (+3) Hearing impairment (+2) Psychotropic drugs (+1)	Arrival via ambulance (+1) Triage chief complaint of AMS (+18) ESI level ≥ 3 (−3) Low oxygen saturation (< 92%) (+2) Low systolic BP < 111 mmHg (+2) High diastolic BP > 99 mmHg (+1) Low RR (< 16/min) (+3) High RR (> 24/min) (+6) Confusion or disorientation identified during fall risk assessment (+25) Altered elimination identified during fall risk assessment (+8) History of seizure disorders (+4)
Interpretation	Score of < 1 is considered negative (low‐risk)	Score of ≥ 4 is considered positive (high‐risk)	Score of ≥ 11 considered positive (high‐risk; Youden's index) Score of < 11 considered negative (low risk; Youden's index) Score of < 5 considered negative (low‐risk; highly sensitive)

Abbreviations: AMS, altered mental status; BP, blood pressure; ESI, emergency severity index; RR, respiratory rate.

##### 3.8.1.2 Direct and Indirect Evidence

Several studies have identified specific risk factors associated with an increased likelihood of prevalent (Pre‐ED) and incident delirium in the ED. Dementia, for example, shows a significant association with delirium, odds ratio (OR) of 3.4 (95% confidence interval [CI] 1.44–8.08) [74]. Cognitive impairment, as defined by abnormal telephone cognitive interview, similarly raised the risk, with an OR of 3.6 (95% CI not reported) [75]. Functional dependence, defined as immobility [76] significantly increases the risk, with a 37% prevalence of delirium among functionally dependent individuals compared to 5% for those who are functionally independent. Sensory impairments, including visual and hearing impairment (OR 8.07, 95% CI 3.80–17.44), are also important predictors of delirium [76]. Drug burden, as measured by the delirium drug scale [76], for example, increases the odds of delirium (OR 1.29, 95% CI 1.16–1.44) [76].

Risk stratification tools that incorporate some of the specific risk factors above have been developed to stratify the risk of prevalent (Pre‐ED) delirium. In an initial study of 303 patients (mean age 74, IQR 69–80), a positive Delirium Risk Score demonstrates a high sensitivity of 96.0% (95% CI 88%–100%) and LR− of 0.08 (95% CI 0.01–0.56) for prevalent (Pre‐ED) or incident delirium in the ED, and a specificity of 49.3% (95% CI 43.4%–55.2%) and LR+ of 1.9 (95% CI 1.6–2.2) [71]. The Delirium Risk Score encompasses the following elements, which are weighted equally: Dementia, functional dependence (Katz Activities of Daily Living ≤ 4), and hearing impairment, and a score of 1 or more is considered positive [71]. A retrospective validation study of 2582 hospitalized patients (indirect evidence) showed sensitivity of 91.2 (95% CI 90.0–92.3), specificity of 50.3 (95% CI 49.3–51.3), LR+ of 1.83 (95% CI 1.79–1.88), and LR− of 0.17 (95% CI 0.15–0.20) [77]. This combination of direct and indirect evidence supports using the Delirium Risk Score to rule out high risk for delirium, identifying those at low risk of developing delirium.

Zucchelli's delirium risk assessment tool [72] was another one reported in the literature. In adults over age 65 admitted to the ED observation unit, investigators evaluated various cutoffs for identifying delirium. The authors stated that the cutoff of 3 is to be used. When using the cutoff of 4, the sensitivity was 78.4% (95% CI not reported in the test set), specificity 82.9%, LR+ 4.6, and LR− 0.26. When using the cutoff of 5, the sensitivity was 73.0%, specificity 91.4%, LR+ 8.5, and LR− 0.30, so this was more specific. We suggest the Zucchelli's risk assessment tool be used at the cutoff of 4 or 5 as opposed to 3 to identify those at low or high risk of delirium to meet the LR threshold.

The REDEEM risk score [73] evaluated various cutoffs for identifying prevalent (Pre‐ED) delirium in patients over 75 years old. When using the high‐risk cutoff of 11, the sensitivity was 84.1% (95% CI 75.5%–90.2%), specificity 86.6% (95% CI 84.1%–88.8%), LR+ 6.29 (95% CI 5.21–7.60), and LR− of 0.18 (95% CI 0.12–0.28). When using the low‐risk cutoff of 5, the sensitivity was 91.6% (95% CI 84.2%–95.8%), specificity 72.7% (95% CI 69.5%–75.6%), LR+ 3.35 (95% CI 2.96–3.79), and LR− was 0.12 (95% CI 0.06–0.22). In adults over 75 presenting to the ED, the REDEEM Score threshold of 11 may be used to identify those at low or high risk of delirium. The low‐risk cutoff of 5 may be used to rule out high risk for delirium. It is important to note that the REDEEM risk score has not been evaluated in ages 65–74.

The Kennedy Risk Prediction Rule [19] is another delirium risk stratification tool. The derivation study included 700 patients (mean age 77, SD8) and used scores of ≤ 2 points, 3 or 4 points, and ≥ 5 points to identify patients at low, moderate, and high risk for delirium, respectively. For high risk ≥ 5, the LR+ was 3.9 (95% CI 2.7–5.4) and the LR− was 0.60 (95% CI 0.46–0.74). For the low risk group (Score ≤ 2), the LR+ was 0.36 (95% CI 0.22–0.56) and LR− was 2.2 (95% CI 1.8–2.5) [19]. A Kennedy Risk Prediction Rule validation study (*n* = 2582) [77] evaluating incident delirium hospital‐wide demonstrated the LR+ for the high‐risk group (score of 5) was 2.98, while the LR− was 0.63 without available confidence intervals. For the low‐risk group (score of 2), the LR+ was 1.86, and the LR− was 0.45. This direct and indirect evidence does not support the use of the Kennedy Risk Prediction Rule to either rule in or rule out high risk of delirium.

Screening older adults for high risk for delirium is recommended by other international expert societies. Although they don't recommend a specific screening tool, the Australian and New Zealand Society for Geriatric Medicine issued a position statement in 2021 that “All older people should be assessed for risk factors for delirium on admission to the hospital,” listing several risk factors identified in our systematic review, including components of the screening tools recommended by our guideline [78]. They did not specifically address the level of evidence associated with this statement.

##### 3.8.1.3 Benefits

The use of a risk stratification tool in the ED would optimize the diagnostic process for delirium by allowing resources to be directed towards the high‐risk group. In the derivation study, a REDEEM risk score of ≥ 11 identified one‐fifth of the older patient population as high‐risk [73], thus theoretically decreasing the screening burden by approximately 80%. The purpose of risk stratification rather than diagnosis further supports the choice of a cutoff LR of > 4 or < 0.4, as aiming for a more significant LR would obviate the need for a subsequent diagnostic tool after the risk stratification. As of the date of these guidelines, no implementation study has directly evaluated the real‐life benefits of risk stratification. Another benefit of identifying patients at high risk for delirium would be prioritizing patients for inpatient beds given the association with ED boarding and delirium [79].

##### 3.8.1.4 Harms and Burden

When delirium diagnosis is targeted only on a subset of patients identified as high‐risk for delirium, this may lead to missed delirium cases and subsequent harm from undertreatment, such as falls, pressure ulcers, dehydration, aspiration, and death [80]. For example, REDEEM score with sensitivity 84.1% at the cutoff of 11, 15.9% of patients with delirium would be identified as non‐high‐risk, and the diagnosis of delirium might be missed [73]. In addition, the reliance on binary results from a delirium‐risk screening could discount a change in mental status not meeting the threshold for delirium [81].

Implementation of these risk stratification tools presents several challenges, most importantly integrating the screening tool into the ED workflow without overwhelming clinicians. Utilizing the Electronic Medical Record (EMR) to automatically flag patients who meet predefined criteria may assist in this task, although not every ED has an EMR, nor do frontline clinicians have direct management decision‐making regarding which screening tools are built into the EMR [82]. Another issue is that this requires elements of the tools to be collected.

##### 3.8.1.5 Decision Criteria and Additional Considerations

There is no direct or indirect evidence on the impact of delirium risk stratification methods on ED older patient outcomes. Training of clinicians, nursing staff, and nursing aids on the use of risk stratification tools and organizational support, such as allocation of staff, time, and money is crucial for successful implementation of any delirium risk stratification tool [83]. More studies are needed to evaluate the effectiveness of these methods in diverse ED settings and their impact on hospital resources and patient care, including cost‐effectiveness studies. ED clinicians can overestimate their innate ability to identify delirium and often lack training in effective communication strategies to convey these concerns to patients already feeling overwhelmed with competing distractions in the chaotic ED. Clinicians suggest that the EMR is underutilized to provide an automated approach to geriatric syndrome screening [82, 84, 85, 86, 87, 88].

##### 3.8.1.6 Equity in Healthcare Delivery

Equity in using delirium risk stratification tools in emergency care hinges on ensuring the tools are valid across diverse populations [33]. Challenges include language barriers, limited caregiver input, and lack of representation of socially disadvantaged groups in tool development [39, 82]. To promote equity, tools must be adapted for diverse populations, consider social determinants of health, and be paired with culturally sensitive training to ensure all patients receive accurate and timely delirium care.

##### 3.8.1.7 Conclusion and Research Needs

Newly emerging or re‐purposed tools, such as Zucchelli's risk assessment tool or REDEEM score, show some promise in identifying high‐ or low‐risk groups for targeted delirium testing, but implementation would require integration into the EMR [89]. Balancing the cost‐effectiveness of introducing delirium screening with the presumed benefit of decreasing screening burden is necessary. In addition, a better understanding of the barriers and facilitators for clinician screening and EMR‐based risk stratification is needed. In the case of the Delirium Risk Score, which has the lowest LR, possible misuse of the tool to identify those who are high‐risk instead of those who are low‐risk (as intended) should be considered. Risk stratification for delirium in the ED is a promising strategy to enhance screening efficiency but requires prospective trials to fully understand clinician acceptance, fidelity, and adaptability along with the real‐world impact on patient‐oriented outcomes associated with the timely recognition and management of delirium. As Seidenfeld et al. mentioned, future research is needed via an implementation science approach to ensure comprehensive assessment of risks and benefits of delirium screening and approaches to streamlining delirium screening, including whether it poses a burden to patients and staff [33]. While we agree that delirium screening should be more prevalent, we balance this with what is actually happening in EDs (minimal screening). We see delirium risk scores as ways EDs and/or clinicians could possibly find a subset of patients that would have formal delirium testing to increase overall screening (e.g., understanding which patients were at high risk via EMR). Delirium risk scores could also identify those at risk of developing delirium for targeted delirium prevention, though there is low certainty of evidence that prevention works in the ED setting [29].

#### 3.8.2 Question 2: Delirium Diagnosis

*Can delirium screening instruments accurately rule in or rule out delirium in older emergency department patients?*

[Evidence to decision framework in Appendix S2].

Recommendation #4: In older adults in the ED, 4AT, bCAM, CAM‐ICU, mCAM, AMT‐4, or RASS may be used to rule in or rule out delirium (Conditional, FOR) [Very low certainty of evidence].

Recommendation #5: In older adults in the ED, DTS may be used to rule out delirium (Conditional, FOR) [Very low certainty of evidence].

##### 3.8.2.1 Summary of Evidence

In older ED patients with suspected delirium, there is sufficient direct evidence to select delirium diagnostic instruments that accurately rule in (i.e., confirm) or rule out (i.e., exclude) delirium [22]. The 4AT had the largest quantity of ED‐based research and may be used to rule in or rule out delirium [90]. The CAM‐ICU, brief Confusion Assessment Method (bCAM), modified Confusion Assessment Method (mCAM) for the Emergency Department, AMT‐4, and Richmond Agitation and Sedation Scale (RASS) are superior to clinical gestalt [91], and may also be used to rule in or rule out delirium. The Delirium Triage Screen is superior to clinical gestalt in ruling out delirium [92] (Table 3).

**TABLE 3:** Delirium screening tools comparison table.

Tool	Components	Use setting	Sensitivity	Specificity	Time to administer	Notes
4AT [92]	Alertness, AMT4 (orientation), attention (MOTYB), acute change/fluctuation	ED, hospital wards	89.7%	64.9%	< 2 min	No training required; good for rapid use; AMT4 is part of this tool.
CAM‐ICU [93]	Fluctuating mental status, inattention, altered consciousness, disorganized thinking	ICU, ED (including ventilated pts)	95%–100%	89%–93%	2–3 min	Adapted for non‐verbal/ventilated patients.
DTS [94]	Inattention via object recall + level of consciousness (RASS)	ED (initial triage)	98% (95% CI 90–100%)	55% (95% CI 50%–60%)	< 30 s	High sensitivity; intended as a rapid rule‐out screen; requires confirmation if positive.
bCAM [94]	Uses CAM algorithm with months backward (MOTYB) and 4 simple yes/no questions	ED	84% (95% CI 72%–92%)	96% (95% CI 93%–97%)	< 2 min	Tailored for ED use, highly specific, brief and reliable.
mCAM [95]	Modified CAM using standardized cognitive tasks to improve reliability	ED, hospital wards	85%–95%	85%–95%	~3–5 min	Includes objective cognitive tests to reduce rater variability.
AMT4 [92]	Orientation questions: age, date of birth, place, year	Used within 4AT and others	Not used alone	Not used alone	< 1 min	Component of 4AT; quick cognitive screen, not a delirium tool by itself.

##### 3.8.2.2 Direct and Indirect Evidence

Our systematic review [22] included 27 studies [18, 19, 70, 71, 91, 92, 93, 94, 95, 96, 97, 98, 99, 100, 101, 102, 103, 104, 105, 106, 107, 108, 109, 110, 111, 112, 113] that examined delirium diagnostic test accuracy in the ED. These studies were included when the original studies provided sufficient detail on the delirium diagnostic test and criterion standard to construct two‐by‐two tables. The meta‐analysis included three studies examining the accuracy of the 4 A's Test (4AT) [101, 108, 109] and found a pooled LR+ of 7.5 (95% confidence interval [CI] 2.7–20.7) to rule in and a pooled LR− of 0.18 (95% CI 0.09–0.34) to rule out delirium. Our meta‐analysis of two studies on Abbreviated Mental Test‐4 (AMT‐4) [70, 99] found a pooled LR+ of 4.3 (95% CI 2.4–7.8) which was lower than that observed for the 4AT with overlapping confidence intervals, but the pooled LR− of 0.22 (95% CI 0.05–1) was similar. FAM‐CAM was also evaluated, but given the LR+ 3.4 (95% CI 1.4–5.4) to rule in and LR− 0.5 (95% CI 0.3–0.7) to rule out delirium [114] which fell below our inclusion threshold of LR+ > 4 and LR− < 0.4, we did not include these in our recommendations. The LR+ for the diagnostic accuracy of these instruments from single studies ranged from 2.2 – 51.3 and 0.0 to 0.37 for LR−. It is worth noting that the included studies did not use the same gold standard and the QUADAS‐2 risk of bias varied, which subsequently affected our certainty of evidence [115].

Several additional delirium diagnostic tools were identified in our systematic review [22]. bCAM was reported in a single center study (*n* = 406) in which ED clinicians accurately rule in delirium (LR+ 19.9 [95% CI 12–33]) and rule out (LR− 0.17 [95% CI 0.09–0.32]) delirium using bCAM [91]. CAM‐ICU was also reported in a single center study (*n* = 406) [91] in which ED clinicians accurately rule in delirium (LR+ 51.3 [95% CI 21–125]) and to a lesser extent rule out (LR− 0.28 [95% CI 0.18–0.44]) delirium. mCAM was also evaluated in a single ED setting (*n* = 286) [103] and ED clinicians accurately rule in (LR+ 39.9 [95% CI 19–60]) and rule out (LR− 0.1 [95% CI 0.02–0.30]) delirium by using mCAM. Han et al. (*n* = 406) evaluated the use of RASS score for delirium detection. ED physicians accurately ruled in (LR+ 5.5 [95% CI 4–7]) and ruled out (LR− 0.21 [95% CI 0.12–0.38]) delirium [18, 22, 91]. Furthermore, Grossman found that among 285 patients, LR+ 10.31 (95% CI 6.06; 17.51), LR− 0.32 (95% CI 0.16; 0.63) for mRASS other than 0 when tested by trained nurses [113].

DTS was assessed in a single‐center study (*n* = 406) [22, 91] demonstrating that ED clinicians using DTS inaccurately rule‐in delirium (LR+ 2.2 [95% CI 1.9–2.5]) but can rule‐out delirium (LR− 0.04 [95% CI 0.01–0.25]) [22]. Indirect evidence from settings beyond the ED was concordant with our findings. A systematic review of 17 studies (3702 observations) examining the 4AT in acute medicine, surgery, care home, and the ED setting reported pooled sensitivity 0.88 (95% CI 0.90–0.93) and pooled specificity 0.88 (95% CI 0.82–0.92) indicating good diagnostic accuracy [116]. Miranda et al. [117] conducted a Cochrane review of 25 studies (2817 participants) of the CAM‐ICU for the diagnosis of delirium in the intensive care unit and estimated a pooled sensitivity of 0.78 (95% CI 0.72–0.83) and pooled specificity of 0.95 (95% CI 0.92–0.97). This indirect evidence further strengthened our findings, as it is unlikely that the setting of tool administration (i.e., ED vs. inpatient setting) significantly affects the tool's accuracy.

Ultimately, our delirium testing recommendations are congruent with the recommendations of other international expert societies. The British Geriatrics Society encourages urgent care services to actively look for delirium, specifically recommending bCAM, CAM‐ICU, and the 4AT. They recognize the role of RASS as well, noting that it has decreased diagnostic accuracy compared with the aforementioned tools [118]. The Australian and New Zealand Society for Geriatric Medicine issued a position statement in 2021 that “those who display altered cognition should be screened for delirium using a validated tool such as the Confusion Assessment Method or 4 A's Test” [78]. It is worth noting that neither of these societies employed the GRADE methodology and did not address the level of evidence associated with their statements.

##### 3.8.2.3 Benefits

There were no ED‐based studies reporting on patient‐centered outcomes of delirium recognition. However, leveraging accurate delirium diagnostic tests in the ED could identify those with or without delirium earlier, which could theoretically lead to quicker evaluation of the etiology of delirium and reduce the duration or severity of episodic delirium [20, 119]. Prior research suggests that early delirium detection and intervention, such as avoiding benzodiazepines and antipsychotics and environmental adjustments, can reduce hospital length of stay by 2 days and decrease delirium‐related falls from 23.4% to 17% [120]. It is worth emphasizing that the value of delirium diagnosis lies in the downstream effect of managing the identified delirium. This benefit is negated in settings where delirium management is not addressed after positive identification, such as in low‐resource settings or during ED overcrowding.

##### 3.8.2.4 Harms and Burden

Some patients expressed frustration when the delirium protocol questions are perceived as irrelevant to their medical condition [121]. Missing delirium in older ED patients (i.e., a false negative test) leads to undertreatment, with subsequent falls, pressure ulcers, dehydration, aspiration, and death [80]. On the other hand, overdiagnosing delirium (i.e., a false positive test) may result in an increased ED length of stay, care delays, anchoring bias, additional testing, and possible unnecessary hospitalization [122]. Until a delirium diagnostic tool that is both highly sensitive and specific and easy to implement is created, these aforementioned risks can be mitigated by choosing delirium diagnostic tools with high sensitivity and confirming the results with a highly specific diagnostic test [92].

The direct cost associated with performing these diagnostic tests is the time required for completion, as well as the time and cost required for training. However, all these delirium diagnostic instruments require less than 5 min to perform, with the DTS and RASS requiring less than 30 s. It appears that some ED clinicians are willing to spend up to 5 min on a similar task, and most are willing to spend less than 2 min [123], highlighting the importance of the brevity of the chosen tool. The time required for adequate training applying these delirium instruments and maintaining competency to use them with high fidelity is unknown outside the research settings where opinion leaders provided one‐time training to research assistants and/or clinicians.

##### 3.8.2.5 Decision Criteria and Additional Considerations

Diagnostic accuracy studies have mostly been conducted in academic centers, which limits the external validity to other settings, such as community EDs or low‐resource settings. Applying these diagnostic instruments in less‐resourced non‐research settings without local opinion leaders or delirium expertise may yield differing levels of instrument acceptance and fidelity, accuracy, and overall impact in reducing the sequelae of delirium [115, 124, 125]. It is also unclear if the diagnostic accuracy of these tools differs across delirium motor subtypes (hyperactive vs. hypoactive), or in identifying incident versus prevalent (pre‐ED) delirium.

Local resources and sustainability must be taken into consideration when implementing a delirium diagnosis program to ensure its success. Proactively addressing previously identified implementation barriers [29, 84] by utilizing electronic reminders, selecting a shorter test, and clarifying how to address a positive result may improve the adherence of ED clinicians to screening. Recognizing that language barriers may hinder the detection of delirium [22] may necessitate additional resources (e.g., in‐person interpreters, diverse healthcare workers) to maintain equity in healthcare delivery. The discordance in the language between the clinical staff and the patient/caregiver is thought to be the cause of the barrier, as some of these instruments have been translated to languages other than English [84, 85, 86, 88, 126, 127].

The inclusion of RASS as a delirium diagnosis tool, although only focusing on the level of consciousness, addresses several shortcomings of traditional tools, such as language barrier, time for completion, and requiring additional collateral history which may not be available. In addition, the assessment of the level of consciousness is an essential part of several other tools, such as 4AT, bCAM, CAM‐ICU, and mCAM.

Additionally, recommendations #4 and #5 specified ED physician or nurses' administration of tests, given that the evidence for those recommendations only assessed ED physicians or research assistants. It could be that other ED clinicians such as APPs could administer these delirium tests with training to assure similar proficiency.

##### 3.8.2.6 Conclusions and Research Needs

In older ED patients with suspected delirium, there is sufficient evidence to select delirium diagnostic instruments that accurately rule in or rule out delirium. Emerging tools, such as UB‐CAM, by the point‐of‐care app in the ED setting, have not yet been validated [128]. Further research is necessary to determine the accuracy of various instruments in subgroups of patients, such as those with differing delirium etiologies, underlying dementia, or different motor subtypes [20, 22]. Cost analyses and cost‐effectiveness research, including nurse/physician training requirements, are needed to determine the required resources for implementation. Implementation Science research exploring the acceptability and feasibility of ED delirium screening in addition to fidelity, adaptability, and sustainability of screening protocols is lacking [129, 130].

#### 3.8.3 Question 3: Delirium Evaluation

*Should older adults with delirium in the ED be evaluated with a noncontrast head computed tomography* (*CT*) *or not?*

[Evidence to decision framework in Appendix S3].

Recommendation #6: In the undifferentiated older ED patient with delirium or altered mental status, we have insufficient data to recommend ordering a head CT versus not ordering a head CT (Conditional recommendation, EITHER) [Very low certainty of evidence].

##### 3.8.3.1 Summary of Evidence

Delirium may be associated with many disease processes or medication interactions, including neurological etiologies such as stroke, intracranial hemorrhage, and brain tumors [131]. Often, the etiology for delirium is undifferentiated in the ED. Obtaining a head CT is common practice in older adults presenting to the ED with altered mental status (AMS), occurring in 13%–23% [132, 133]. No clear guidance exists on whether to obtain a head CT in older adults presenting to the ED with delirium. The American College of Radiology (ACR) Appropriateness Criteria recommend obtaining brain imaging (specifically, a non‐contrast head CT) in patients with AMS, coma, delirium, or acute psychosis “unless the etiology is clear and the risk of intracranial pathology is low” [134].

However, it is unclear how to quantify the risk of stroke, intracranial hemorrhage, or brain tumor in patients presenting with delirium when traditional flags such as focal neurologic deficits or acute trauma are absent. In addition, the recommended timing of this imaging is unclear, and the guidance is not specific to the ED, leaving the decision to the evaluating clinician's judgment.

The yield of head CT for acute contributory findings in older adults ranged from 2% to 45%. Different studies found variable levels of associations between abnormal head CT and risk factors, such as trauma, falls, or anticoagulation. The Australian and New Zealand Society for Geriatric Medicine recommends the avoidance of head CT as part of the delirium workup in the absence of trauma, anticoagulation use, or focal neurological deficit [78], noting that their statement is not specific to the ED setting, and they did not include a strength of recommendations.

##### 3.8.3.2 Direct and Indirect Evidence

There are no studies on the association between the diagnosis of delirium in older adults in the ED and abnormal CT head imaging, most likely due to the underdiagnosis of delirium in the ED [135]. Therefore, AMS or confusion was used as surrogates for delirium, because AMS has a high specificity (98.9%) for delirium [17].

The systematic review [42] identified two observational studies [136, 137] (total 189 patients) reporting on the association between confusion or AMS in older ED patients and abnormal head CT findings (defined as stroke, intracranial hemorrhage, or brain tumor). The meta‐analysis found no significant association between confusion/AMS and abnormal head CT (OR 0.35, 95% CI 0.031 to 3.97) [42]. Additional predictors of head CT abnormalities in delirious ED patients have not been well studied. For example, anticoagulant use has been proposed as a risk factor but was not associated with an abnormal head CT scan in a systematic review of 909 patients in 6 studies [34]. Headache was also proposed as a predictor, but no sufficient data were present to examine this association [34]. Only the presence of a focal neurological deficit was significantly associated with an abnormal head CT in this population (OR 102.0, 95% CI 30.0–340.0) [34].

The avoidance of head CT as part of the routine delirium workup in the absence of other factors, as recommended by the Australian and New Zealand Society for Geriatric Medicine [78], is based on 3 inpatient studies examining patients with confusion and/or delirium; hence, they were not included in our SR. The indirectness of these recommendations, based on inpatient research with potentially limited generalizability to the ED, is offset by our GED Guidelines 2.0 systematic review of ED‐based studies, which states that there is insufficient evidence to determine the need.

##### 3.8.3.3 Benefits

The prevalence of stroke, hemorrhage, or brain tumor on head CT in older adults in the ED presenting with AMS, confusion, or delirium was 13%–16% [34, 132]. Correctly diagnosing patients with stroke, intracranial hemorrhage, or tumor confers the benefit of offering timely and appropriate treatment. Age alone does not affect the benefits of treatments for acute ischemic stroke; older adults receive the same benefits as their younger counterparts with timely treatment [138]. Intracranial bleeding has a high mortality rate, 9.6% for hospitalized older adults [139], so early diagnosis is important to ensure timely intervention.

A study of trauma patients [140] showed 91% preferred a head CT and associated radiation exposure to detect acute life‐threatening traumatic injuries when the odds of such an injury were 25%. Seventy‐nine percent preferred head CT over none when the probability of the serious injury was 10%. Obtaining a head CT aligned with the value of one of our care partner representatives, while the other commented that it is important to determine what matters most to the patients and their care partners.

##### 3.8.3.4 Harms and Burdens

Indiscriminately ordering head CTs on all older adults with delirium will lead to an increase in radiation exposure and cost with an unclear benefit [141]. Longer ED stays associated with imaging delays may contribute to worsening delirium [142]. An additional harm in those with hyperactive delirium includes the need for sedation to facilitate obtaining the imaging, with its downstream effects of worsening delirium, falls, dizziness, respiratory complications, and further increasing length of stay (LOS) [143]. A normal head CT report could lead to premature closure for evaluation of delirium etiology with misdiagnosis of alternative life‐threatening conditions [144].

##### 3.8.3.5 Decision Criteria and Additional Considerations

Head CTs are routinely ordered for patients with AMS and delirium in clinical practice [34, 42]; up to 24% of patients presenting to the ED with AMS may receive a head CT, and it appears that the number of head CTs ordered for this indication has increased since 2000 [132, 133]. We sought to determine if head CT utilization could be safely decreased in patients with delirium, but we did not find direct or indirect evidence upon which to base such a recommendation. The data are limited by small study samples and bias. For example, no study included unselected ED patients with AMS. Instead, these studies include only patients whose clinician chose to order a head CT, which likely introduces both spectrum bias and differential verification bias [115]. A focal neurological deficit was significantly associated with the diagnosis of stroke, intracranial hemorrhage, or brain tumor on CT, but overall the certainty of the evidence is low to either recommend for or against routine CT imaging in older adults with suspected delirium in ED settings. There is no clear delineation on the benefits and harms of obtaining the head CT in the ED versus inpatient.

##### 3.8.3.6 Equity in Health Care Delivery

Availability and expense are two factors that affect the ability to access CT. We did not identify any article focused on equity in terms of using head CT imaging in delirious patients [36]. Patients in rural/remote areas and in lower socioeconomic groups may have more difficulty accessing timely imaging or affording imaging [36, 145, 146]. Overall, given the lack of direct or indirect evidence, it is unclear how our recommendation would impact equity in health care delivery.

##### 3.8.3.7 Conclusions and Research Needs

The GED Guidelines 2.0 Delirium Group concludes that there is insufficient evidence to recommend for or against routine head CTs in older adults presenting to the ED with delirium or AMS. Approximately 15% of patients with AMS who undergo head CT have acute abnormalities, which may be an etiology for their AMS, but real‐world diagnostic yield across all delirious patients is likely lower because our recommendations are derived from a population who underwent head CT because the ED clinician recognized a need for that CT. Research is needed to identify predictors of abnormal CT findings associated with delirium, to assess quality‐of‐life impacts of brain imaging, and analyze cost‐effectiveness to guide imaging decisions for older adults with delirium.

## 4 General Issues Necessary for Correct Interpretation and Implementation of Recommendations

### 4.1 Limitations

The most significant limitation is the paucity of high‐quality minimally biased ED‐based studies, with resulting reliance on observational and indirect evidence, making the certainty of evidence of the recommendations low. This includes the limited precision of risk stratification and delirium diagnostic tools as their 95% CI crossed our proposed cutoffs. Practical issues such as ethical difficulty in obtaining consent in patients with delirium, as well as the well‐documented underdiagnosis of delirium in the ED population [147] may be barriers to high‐quality research [148]. Another important limitation is the lack of reported data on patients' values as it relates to delirium diagnosis and workup, which is inherently difficult to study as some patients may lack the cognitive skills to assess and communicate their preference. Our care partner representatives informed our understanding of the caregivers' perspective for ED delirium approaches, but no systemic large‐scale research exploring values and preferences is available. Furthermore, no cost effectiveness research for ED delirium approaches exists, indicating a need for such research once efficacious approaches are confirmed. Also, while we endeavored to include perspectives from multiple countries, these guidelines may not be generalizable to all healthcare systems. Lastly, there is insufficient evidence to identify a reliable and sustainable mechanism that would facilitate any monitoring or auditing process, and so the proposal of any such criteria would be premature.

### 4.2 Implementation Considerations

Clinical practice guidelines are meant to inform management decisions for patients, but not to replace a clinician's judgment. In clinical practice, several factors can result in deviation from guideline recommendations, most commonly due to a lack of resources [149, 150, 151, 152]. For example, if the patient with delirium does not have an obvious cause for their delirium and is at a facility where a head CT is not available, the clinician may choose to observe the patient in lieu of transferring them to a different facility.

As for identifying patients who are high‐risk for delirium, as well as screening patients for delirium, implementation will require staff time, education costs, as well as an EMR to discover data, extract information, and risk‐stratify older adults for delirium. An important implementation consideration is the downstream effect of potential increased delirium identification. For example, increased identification of delirium may increase hospital admissions, further straining hospital capacity. These GED 2.0 Guidelines do not address the management of delirium given the paucity of ED‐based studies examining the impact of interventions on patient‐centered outcomes [29]. The recommendation relating to head CTs is not meant to increase the already high utilization of head CTs in this population, but rather acknowledge the paucity of evidence *for* obtaining the head CT in the undifferentiated confused older adult.

### 4.3 Future Directions

Interventions to identify, assess, prevent, and treat delirium in the ED are being developed and tested in the ED setting. A working group of content experts convened to develop a point‐of‐care tool to assist emergency physicians in the care of older adults in the ED, and it was designed to present and explain the Assess, Diagnose, Evaluate, Prevent, and Treat (ADEPT) [153]. Five core principles were identified by the group that ensure adequate and thorough care for older adults with delirium, though the ADEPT tool remains a theoretical consensus document needing objective assessment. Developed by ACEP, the ADEPT tool [153] gives collective knowledge from an expert panel on delirium evaluation and management. A systematic review on delirium intervention found that few ED‐initiated interventions decreased the duration or incidence of delirium [29]. One observational cohort study on the use of ED Foley catheters in the ED increased the duration of delirium by 1–2 days [154]. Another observational cohort reported that higher physical therapy and occupational therapy intensity may reduce delirium duration by nearly 60% in older ED patients who are hospitalized [155]. These GED 2.0 Guidelines are current as of January 2025. Now that clear knowledge gaps have been identified, high‐quality primary evidence will be periodically assessed to inform future updates of these guidelines [156, 157].

## Conflicts of Interest

All group members disclosed conflicts of interest, listed below in alphabetical order. All members were able to participate as a voting member with the following disclosures and management. Funders had no influence on the content of this document. Christopher R. Carpenter, MD, MSc: Serves on the Clinician Scientists Transdisciplinary Aging Research Leadership Core and the American College of Emergency Physicians' Geriatric Emergency Department Accreditation Advisory Board. Kerstin de Wit, MD: Nothing to disclose. Debra Eagles, MD, MSc: Nothing to disclose. Maura Kennedy, MD, MPH: Serves on the American College of Emergency Physicians' Geriatric Emergency Department Accreditation Board of Governors. Although a volunteer position, monetary remuneration is occasionally received on behalf of work done for the program. Danya Khoujah, MBBS, MEHP: Nothing to disclose. Sangil Lee, MD, MS: Nothing to disclose. Shan W. Liu, MD, SD: Serves on the American College of Emergency Physicians' Geriatric Emergency Department Accreditation Advisory Board, as well as the International Federation of Emergency Medicine Geriatric Emergency Medicine Special Interest Group. Alexander Lo, MD, PhD: Nothing to disclose. Ines Luciani‐Mcgillivray, RN: Nothing to disclose. Christian H. Nickel, MD: Serves as a member of the geriatric section of the European Society of Emergency Medicine (EUSEM). Luna Ragsdale, MD: Serves on the American College of Emergency Physicians' Geriatric Emergency Department Accreditation Board of Governors. Justine Seidenfeld, MD, MHS: Nothing to disclose.

## Supporting information


**Appendix S1:** PICO 1 Evidence to Decision document.
**Appendix S2:** PICO2 Evidence to Decision document.
**Appendix S3:** PICO3 Evidence to Decision document.

## Data Availability

The data that support the findings of this study are available on request from the corresponding author. The data are not publicly available due to privacy or ethical restrictions.
