# Supplementary material for: GRADE‐Based Clinical Practice Guidelines for Emergency Department Delirium Risk Stratification, Screening, and Brain Imaging in Older Patients With Suspected Delirium
Source: Acad Emerg Med. 2025 Oct 27;33(2):e70167. doi: 10.1111/acem.70167 (PMC12875304; doi:10.1111/acem.70167)

Appendix S1. **PICO 1 Evidence to Decision document**

# **Question**

#

| **Can risk stratification methods identify a subset of older adults in the ED at high risk for delirium for targeted delirium testing?** | |
| --- | --- |
| **Population:** | P=All older adults (65 years of age or older) in the ED |
| **Intervention:** | I=Use of a risk stratification method to identify a subset of patients at higher risk for delirium  C=No use of risk stratification  O=Proportion of older adults that would require additional delirium testing, sensitivity and/or specificity of risk stratification method |
| **Purpose of the test:** | A screening instrument, algorithm, or artificial intelligence platform to identify high risk patients for further in-depth delirium evaluation to reduce the burden of delirium testing among frontline healthcare teams |
| **Role of the test:** | To improve efficiencies in the detection of delirium by decreasing the overall screening burden |
| **Linked treatments:** | None currently  Few ED initiated interventions have been found that consistently reduce the incidence or duration of delirium Lee et al.[^1^](https://paperpile.com/c/6qpfgj/6ef68) |
| **Anticipated outcomes:** | More efficient delirium testing compared to universal screening  Higher levels of acceptability and compliance with delirium screening associated with scales of efficiency Higher positive predictive value of delirium screening |
| **Setting:** | Emergency Department |
| **Perspective:** | Patient, care partner, nurse, physician, and non-physician practitioner |
| **Background:** | Incident and prevalent (Pre-ED) delirium are prevalent geriatric syndromes affecting 6%-38% of older adults in the ED.[^2,3^](https://paperpile.com/c/6qpfgj/9SzrO+jWNOV)  Delirium screening enables more accurate detection of delirium than gestalt only, but routine use of time-consuming tests is burdensome and under-utilized.[^4–7^](https://paperpile.com/c/6qpfgj/NAIKg+S3ztw+Ya11E+ctokE) In an era of increasing patient volumes, prolonged lengths of stay, and hospital boarding issues, ED clinicians need to rule in or rule out delirium efficiently and pragmatically, to focus more in-depth testing to identify acute and time-dependent treatable pathology. Available approaches to identify older adult subsets at high-risk for delirium might include artificial intelligence or machine learning platforms that leverage electronic health records, more traditional clinical decision instruments, or individual patient-level risk factors.[^8,9^](https://paperpile.com/c/6qpfgj/5l1lo+GmSAc) We aim to review the evidence on risk stratification approaches that identify which patients are at high risk for delirium so that screening efforts can be focused on those patients, thereby making the screening process more efficient. |
| **Subgroups:** | Dementia |
| **Conflict of interests:** | **Maura Kennedy, MD, MPH:** Serves on the American College of Emergency Physicians’ Geriatric Emergency Department Accreditation Board of Governors. Although a volunteer position, monetary remuneration is occasionally received on behalf of work done for the program.  **Sangil Lee, MD, MS:** Nothing to disclose.  **Shan W. Liu, MD, SD:** Serves on the American College of Emergency Physicians’ Geriatric Emergency Department Accreditation Advisory Board, as well as the International Federation of Emergency Medicine Geriatric Emergency Medicine Special Interest Group.  **Christian H. Nickel, MD:** Serves as a member of the geriatric section of European Society of Emergency Medicine (EUSEM).  **Luna Ragsdale, MD:** Serves on the American College of Emergency Physicians’ Geriatric Emergency Department Accreditation Board of Governors.  **Justine Seidenfeld, MD, MHS:** Nothing to disclose. |

# **Assessment**

| **Problem** Is the problem a priority? | | |
| --- | --- | --- |
| **Judgement** | **Research evidence** | **Additional considerations** |
| ○ No  ○ Probably no  ○ Probably yes  X Yes   ○ Varies  ○ Don't know | The incidence of delirium in geriatric ED patients ranges from 6-38% of older ED patients.[^2^](https://paperpile.com/c/6qpfgj/9SzrO) Because the number of older adults presenting to the ED will continue to increase for decades[^10,11^](https://paperpile.com/c/6qpfgj/MhNwl+R1tBt) which combined with boarding may exacerbate the frequency and severity of episodic delirium,[^12,13^](https://paperpile.com/c/6qpfgj/PUMbU+rEjwf) clinical practice guidelines cognizant of the resources and capacity of contemporary emergency care in the context of evaluating older patients with delirium are currently lacking. | Other societies have clinical practice guidelines for delirium evaluation and management (e.g. trauma, ICU patients)[^14–16^](https://paperpile.com/c/6qpfgj/D2Jgu+XzEba+sHaXF) but these guidelines are not necessarily applicable to the ED.  Attributing mortality and morbidity solely to delirium is problematic in that the confounding influence of the illness or injury severity associated with the episode of delirium is not accounted for. Nonetheless, three decades of research seem to indicate a signal of preventable harm associated with missed delirium in the ED. Lewis et al reported an unadjusted increased 3-month mortality among patients discharged from the ED with delirium from 8% in the non-delirium group to 14% in those with delirium.[^17^](https://paperpile.com/c/6qpfgj/jwI9n) Kakuma et al reported a 6-month mortality when delirium was missed in the ED of 30.8% which was statistically significant when adjusted for age, sex, number of medications, and functional level (Hazards Ratio 7.24; 95% CI 1.62-32.35).[^18^](https://paperpile.com/c/6qpfgj/rimA8) Hustey et al noted that ED physicians did not alter management or disposition plans when informed of the presence of delirium and 26% of those with delirium were discharged home with 80% of those returning to the ED within 4-days.[^19^](https://paperpile.com/c/6qpfgj/JHQUF) Lee et al. noted that 50% of delirium cases were missed and that 6.7% of those with unrecognized delirium died within 1-week compared with 0% of those with delirium recognized.[^20^](https://paperpile.com/c/6qpfgj/VlXmK) Factors associated with safe ED discharge for persons with delirium have remained elusive.[^21^](https://paperpile.com/c/6qpfgj/gCBAE) |
| **Test accuracy** How accurate is the test? | | |
| **Judgement** | **Research evidence** | **Additional considerations** |
| ○ Very inaccurate  ○ Inaccurate  ○ Accurate  ○ Very accurate  x Varies  ○ Don't know | Seidenfeld et al.[^22^](https://paperpile.com/c/6qpfgj/fhVvn) showed a total of 19 studies evaluating diverse risk factors or combinations of risk factors, considered heterogeneous and not amenable for meta-analysis. Patients with dementia or functional, visual, and hearing impairments, high scores on the delirium risk stratification tools, and ED boarding are all high-risk for prevalent (Pre-ED) delirium and may be screened for delirium. There was no consensus, no multi-center implementation, or effectiveness study. Few included studies reported sensitivity or specificity.    A list of risk factors include;  dementia, odds ratio (OR 3.4, 95% confidence interval[CI]1.44-8.08),[^23^](https://paperpile.com/c/6qpfgj/PNjHa) functional dependence (prevalence 37% for functional dependent vs 5% for independent), delirium drug score (OR 1.29, 95%CI 1.16-1.44).[^24^](https://paperpile.com/c/6qpfgj/NB3GJ) Also, various sensorial impairments, such as hearing impairments, which can be seen in up to 28%,[^25^](https://paperpile.com/c/6qpfgj/suUPT) increased the odds of delirium (OR 8.07, 95%CI 3.80-17.44, [^26^](https://paperpile.com/c/6qpfgj/NyrS4) cognitive impairment (OR3.6).[^27^](https://paperpile.com/c/6qpfgj/QyjO1) ED boarding is not limited to older adults, but the risk of delirium increases with a prolonged ED length of stay, as reported by Emond et al.[^28^](https://paperpile.com/c/6qpfgj/1WkO6)  The accuracy of delirium scores for incident delirium:[^29^](https://paperpile.com/c/6qpfgj/f1q3P) Delirium risk score has area under the curve (AUC) 0.8 (95%CI 0.79-0.81), (-LR of 0.08, +LR1.8), Sensitivity and specificity of 91.2 and 50.3, Kennedy risk prediction rule has AUC 0.74, 95%CI 0.73-0.75, sensitivity and specificity of 72.5 and 61 for moderate and high vs low risk group, and high vs moderate and low risk group of 47.1 and 84.2. For high risk ≥5 the calculated were LR+=3.9 (95%CI 2.7-5.4), LR- =0.60(95% CI 0.46-0.74) and for low risk ≤2 the computed LR+ = 0.36 (95% CI 0.22-0.56) and LR- = 2.2 (95% CI 1.8-2.5), neither of which meets our point estimate threshold of 4, though the threshold >5 does cross 4 with the 95% CI.    The Zucchelli’s delirium risk assessment tool was another one reported in the literature. In adults over age 65 admitted to the ED observation unit, investigators evaluated various cutoffs for identifying delirium. When using the cutoff of 4, the sensitivity was 78.4% (95% CI not reported in test set), specificity 82.9%, LR+ 4.6, and LR− 0.26. When using the cutoff of 5, the sensitivity was 73.0%, specificity 91.4%, LR+ 8.5, and LR− 0.30. We suggest the Zucchelli’s risk assessment tool be used at the cutoff of 4 or 5 to identify those at low or high risk of delirium.  The REDEEM risk score[^30^](https://paperpile.com/c/6qpfgj/QN6n1) has been evaluated at various cutoffs for identifying prevalent (Pre-ED) delirium; using the high-risk cutoff ( ≥11), the sensitivity was 84.1% (95%CI 75.5%-90.2%), specificity 86.6% (95%CI 84.1%-88.8%) LR+ 6.29 (95%CI 5.21-7.60) and –LR of 0.18 (95%CI 0.12-0.28), while when using the low-risk cutoff ( ≥5), the sensitivity was 91.6% (95%CI 84.2%-95.8%), specificity 72.7% (95%CI 69.5%-75.6%), LR+ 3.35 (95%CI 2.96-3.79), and –LR was 0.12 (95%CI 0.06-0.22). | Numerous studies exist to evaluate risk factors, as well as develop and validate risk factor combinations. For example, Inouye et al.[^31^](https://paperpile.com/c/6qpfgj/bRrHG) reported the combination of vision impairment, severe illness, cognitive impairment, and high BUN and creatinine ratio to predict delirium in hospitalized patients. Some recent examples include Chen et al.,[^32^](https://paperpile.com/c/6qpfgj/KLWhz) who developed risk stratification for adult critically ill patients in ICU and reported AUC 0.78 from 11 risk factors. |
| **Desirable Effects** How substantial are the desirable anticipated effects?  In other words, if the desirable effects of screening for high-risk for delirium were true, how substantial would they be? | | |
| **Judgement** | **Research evidence** | **Additional considerations** |
| ○ Trivial  ○ Small  X Moderate  ○ Large  ○ Varies  ○ Don't know | No direct evidence implementing delirium risk stratification approaches into practice exist to enable quantification of patient-oriented or process-oriented desirable effects. | Increased risk of adverse events for patients discharged with unrecognized delirium and a more focused approach to identifying ED delirium could theoretically reduce these preventable outcomes.[^28^](https://paperpile.com/c/6qpfgj/1WkO6)  A more focused approach concentrating finite ED resources on patients at increased risk for delirium by decreasing may improve the acceptance, fidelity, and feasibility of delirium process improvement efforts[^33^](https://paperpile.com/c/6qpfgj/2Mydk) based on indirect evidence from other studies of screening burden.  Qualitative studies of delirium screening highlight that ED nurses and physicians reported several barriers to successful delirium screening and management. Specific challenges included difficulty with using delirium screening tools, an organizational culture not conducive to delirium prevention (eg variable institutional priority), and competing clinical priorities.[^5,7,9,33–35^](https://paperpile.com/c/6qpfgj/2Mydk+uPf5j+S3ztw+ctokE+g3Amm+GmSAc)  In the initial study, using a REDEEM risk score of ≥11 would have led to identification of one-fifth (20.5%) of the older patient population as high-risk, facilitate screening only this proportion of the ED patients, and theoretically increase a departments capacity to identify incident or prevalent (Pre-ED) delirium.[^30^](https://paperpile.com/c/6qpfgj/QN6n1)  Although the majority vote was for moderate desirable effects, a few group members felt that the effects were large. |
| **Undesirable Effects** How substantial are the undesirable anticipated effects? | | |
| **Judgement** | **Research evidence** | **Additional considerations** |
| ○ Large  ○ Moderate  ○ Small  ○ Trivial  ○ Varies  X Don't know | No direct or indirect evidence implementing delirium risk stratification approaches into practice data/studies exist to enable quantification of patient-oriented or process-oriented undesirable effects. | Labeling an individual patient as non-high risk for delirium when they are instead at increased risk (false negatives) can theoretically lead to premature closure with missed delirium/subclinical delirium and associated harms. [^36^](https://paperpile.com/c/6qpfgj/uo8Zy)  **Downstream undesirable effects of missed delirium:**  Mortality at 6-months is adversely affected when patients are discharged home with delirium.[^18^](https://paperpile.com/c/6qpfgj/rimA8)  Patients discharged home with delirium are likely to return to the ED (80%).[^19^](https://paperpile.com/c/6qpfgj/JHQUF)  Symptoms of delirium may be incorrectly attributed to psychiatric illness, especially in patients with a history of mental illness, with subsequent psychiatric admission rather than medical admission and therefore inadequate medical workup.[^37^](https://paperpile.com/c/6qpfgj/UpvCt)  Delirium superimposed on dementia is associated with decreased accuracy of the older patient's presenting illness and decreased comprehension of ED discharge instructions.[^38^](https://paperpile.com/c/6qpfgj/lD1rU)    Current detection rates are very low in the status quo, therefore although we're going to be missing individuals with delirium as no tool is 100%, this is probably not worse than what we are doing now which is missing 80% with gestalt.  Delirium risk score sets a high sensitivity and it misses 4% of delirium, but the specificity is limited.[^39^](https://paperpile.com/c/6qpfgj/n95pU) Kennedy risk prediction rule showed that 33% of high-risk group would be missed.[^40^](https://paperpile.com/c/6qpfgj/mbpF0) Data from REDEEM shows that 16% of the high risk group would miss cases of delirium.[^30^](https://paperpile.com/c/6qpfgj/QN6n1) |
| **Certainty of the evidence of test accuracy** What is the overall certainty of the evidence of test accuracy? | | |
| **Judgement** | **Research evidence** | **Additional considerations** |
| x Very low  ○ Low  ○ Moderate  ○ High  ○ No included studies  For each instrument:  Delirium Risk Score:  Judgement very low  Kennedy Risk Score:  Judgement very low  Zucchelli’s risk assessment tool:  Judgement very low  REDEEM:  Judgement very low | **Risk Stratification Instruments**  The risk of bias assessments for delirium risk stratification approaches (whether single risk factor or combination of risk factors) are synthesized by Seidenfeld et al.[^22^](https://paperpile.com/c/6qpfgj/fhVvn) Three studies.[^26,41,42^](https://paperpile.com/c/6qpfgj/NyrS4+exmPk+e67ly) were rated as having low risk of bias across all domains, and one study[^43^](https://paperpile.com/c/6qpfgj/7qeto) was rated as having high risk of bias across each of the three domains (source population, outcome assessment, and missing data), high risk of bias, namely spectrum bias, was found most commonly in source population (n = 10), partly due to the research studies recruiting the subjects who were able to consent.  In other words, the patients included in the study are less likely to be sick (as they are able to consent), which is a spectrum bias[^44^](https://paperpile.com/c/6qpfgj/yZVKw) that artificially inflates the specificity observed in the study results compared to applying the delirium risk stratification approach in real-world ED populations where patients would not need to consent to be screened with one of these delirium risk stratification instruments in a non-study setting and would include a greater mix of “sick” patients at risk for delirium. The already low specificities (delirium risk score 49%) would be even lower when applied to a more general (sicker) ED population. The specificity for REDEEM (87% for score >11) would also be lower in an actual ED population.  **Individual Risk Factors**  In a systematic review,[^45^](https://paperpile.com/c/6qpfgj/k36K1) 4 delirium risk factors were identified with high certainty: Nursing home residence (4 studies; odds ratio [OR], 3.45; 95% confidence interval [CI], 2.17 to 5.48), [cognitive impairment](https://www.sciencedirect.com/topics/medicine-and-dentistry/cognitive-defect) (7 studies; OR, 4.46; 95% CI, 3.38 to 5.89), hearing impairment (3 studies, OR, 2.57; 95% CI, 1.03 to 6.41), and a history of stroke (3 studies; OR, 3.20; 95% CI, 1.17 to 8.75). | No two studies evaluate the same delirium risk stratification instrument, algorithm, or protocol or provide comparative prognostic accuracy estimates evaluating different approaches on the same patient population.  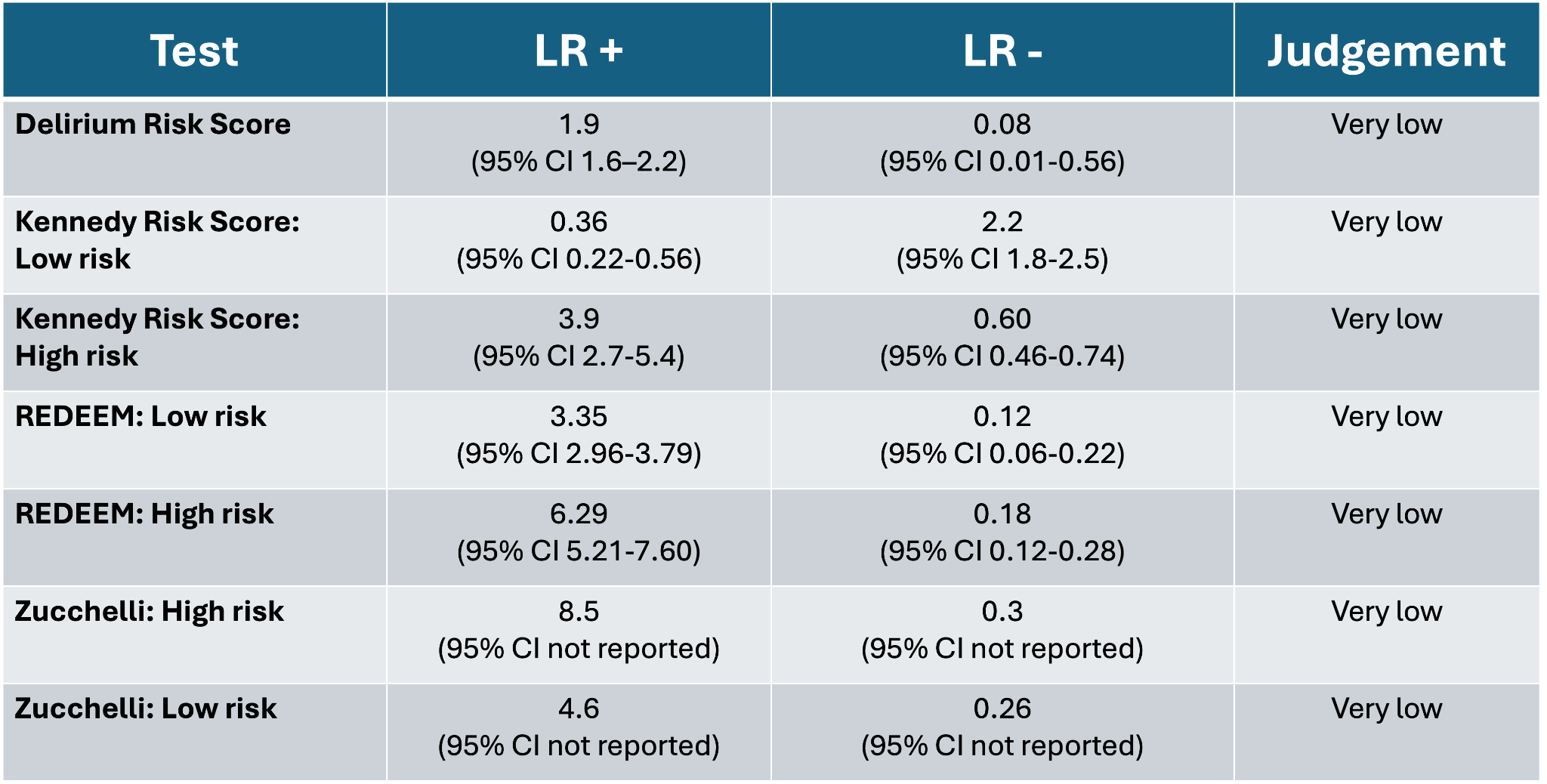 |
| **Certainty of the evidence of test's effects** What is the overall certainty of the evidence for any critical or important direct benefits, adverse effects or burden of the test? | | |
| **Judgement** | **Research evidence** | **Additional considerations** |
| ○ Very low  ○ Low  ○ Moderate  ○ High  X No included studies | No direct or indirect evidence implementing delirium risk stratification approaches into practice data/studies exist to quantify patient-oriented or process-oriented effects or the balance of benefits, adverse effects, or burden of the prognostic stratification approach. | The absence of published evidence evaluating the actual accuracy, feasibility, fidelity, and reliability of delirium risk stratification in the ED setting is a blindspot in evaluating the anticipated applicability and reproducibility of this approach.  No data is present outside of the original or validation studies. |
| **Certainty of the evidence of management's effects** What is the overall certainty of evidence of the effect of the management of delirium (which is the end-goal)? | | |
| **Judgement** | **Research evidence** | **Additional considerations** |
| x Very low ○ Low ○ Moderate ○ High ○ No included studies | Insufficient direct or indirect evidence that current delirium prevention or treatment management of delirium are effective. Acknowledging the absence of evidence, maintaining the status quo is also not the correct approach. | The absence of published evidence evaluating the real world application and impact of delirium risk stratification in the ED setting is a blindspot in evaluating the anticipated output of this approach.  The lack of demonstrably efficacious delirium prevention or treatment options for the management of ED delirium is another issue to establishing a link between delirium risk stratification and management effectiveness.[^1,2,6^](https://paperpile.com/c/6qpfgj/6ef68+Ya11E+9SzrO) |
| **Certainty of the evidence of test result/management** How certain is the link between test results and management decisions (decision to screen for delirium)? | | |
| **Judgement** | **Research evidence** | **Additional considerations** |
| ○ Very low  ○ Low  ○ Moderate  ○ High  X No included studies | No direct or indirect evidence implementing delirium risk stratification approaches into practice data/studies exist to quantify patient-oriented or process-oriented effects or the balance of benefits, adverse effects, or burden of the prognostic stratification approach so unable to comment upon certainty of the evidence linking the delirium risk stratification result and subsequent ED management of delirium. | Based on one test-treatment threshold analysis based on several assumptions quantifying approaches to recognizing and treating delirium,[^3^](https://paperpile.com/c/6qpfgj/jWNOV) if the probability of delirium before screening with instruments such as 4AT is less than 2%, then the risks of delirium screening outweigh the benefits. If the probability of delirium before screening with 4AT is greater than 11% then the risks of delirium screening are outweighed by the benefits of initiating (hypothetical because demonstrably effective ED treatment or prevention strategies are currently lacking[^1,6^](https://paperpile.com/c/6qpfgj/6ef68+Ya11E) delirium intervention without screening. |
| **Certainty of effects** What is the overall certainty of the evidence of effects of the test? | | |
| **Judgement** | **Research evidence** | **Additional considerations** |
| ○ Very low  ○ Low  ○ Moderate  ○ High  X No included studies | No direct or indirect evidence implementing delirium risk stratification approaches into practice data/studies exist to quantify patient-oriented or process-oriented effects or the balance of benefits, adverse effects, or burden of the prognostic stratification approach so unable to comment upon certainty of the evidence of effects of the test. | The assumption that implementing only an accurate delirium risk stratification instrument will alter disease outcome in isolation of any other interventions is problematic, as diagnosis of a medical condition alone does not change patient outcomes.  Future efforts to quantify effects attributable to delirium risk stratification may require patient-oriented randomized trials to control for myriad confounders of screening impact.[^46^](https://paperpile.com/c/6qpfgj/KNDVd) Although diagnostic RCTs are common and sometimes suggest benefits on patient-important outcomes (such as mortality, morbidities, symptoms/quality of life/functional status), they are often limited by sample size and bias.[^46^](https://paperpile.com/c/6qpfgj/KNDVd) |
| **Values** Is there important uncertainty about or variability in how much people value the main outcomes? | | |
| **Judgement** | **Research evidence** | **Additional considerations** |
| ○ Important uncertainty or variability X Possibly important uncertainty or variability  ○ Probably no important uncertainty or variability  ○ No important uncertainty or variability | Clinical gestalt by itself continues to miss delirium in the ED.[^47^](https://paperpile.com/c/6qpfgj/qtdDx) Lack of prevention and treatment for delirium in the ED makes it difficult to justify more screening effort for delirium in the ED.[^1^](https://paperpile.com/c/6qpfgj/6ef68) A qualitative study showed that delirium was recognized as an important clinical problem among ED nurses; however, it was not clinically prioritized;[^48^](https://paperpile.com/c/6qpfgj/tNx5X) Multiple qualitative studies consistently demonstrate a false self-perception of knowledge and ability to recognize delirium and hospital culture as a strong influencer of behavior.[^5,7,9,34,35,48^](https://paperpile.com/c/6qpfgj/tNx5X+uPf5j+S3ztw+ctokE+g3Amm+GmSAc) | **Physicians Values**  In a survey of 197 ED physicians, half of respondents reported neutrality or low levels of concern about discharging a patient with unrecognized delirium and most (64%) noted clinical time constraints in diagnosing and managing delirium.[^5,34^](https://paperpile.com/c/6qpfgj/uPf5j+S3ztw) Lack of delirium documentation, admission, or referral by emergency physicians suggests a lack of recognition of this important problem.[^49^](https://paperpile.com/c/6qpfgj/NVOP5)  **Nursing Values**  Nursing willing to screen, but skeptical whether management or outcomes altered in crowded EDs with many patients in the hallway unless those with or at high-risk for delirium are moved into quiet rooms. [Expert opinion]  ED delirium prevention initiatives are largely driven by bedside nurses and occur on an ad hoc basis, rather than systematically[^7^](https://paperpile.com/c/6qpfgj/ctokE). A focus group study of nurse difficulty with using delirium screening tools, an organizational culture not conducive to delirium prevention, and competing clinical priorities.[^33^](https://paperpile.com/c/6qpfgj/2Mydk)  Notable facilitators of successful implementation of delirium screening report having institutional and ED leadership support and designated clinical champions to longitudinally engage and educate frontline staff.[^9^](https://paperpile.com/c/6qpfgj/GmSAc)  **Healthcare systems values**  The Centers for Medicare & Medicaid Services released a new measure designed to improve the care of and outcomes of older adult patients. The measure will evaluate hospitals’ progress toward improving care of older patients and has 5 domains, one of which is screening for cognitive impairment including delirium. Hospitals could face penalties if they are not compliant.[^50^](https://paperpile.com/c/6qpfgj/Fhoca)  Healthcare systems values are most likely variable depending on the specific setting. Heterogeneity exists as per expert opinion and anecdotal data.  **Patient Stakeholder Values**  Caregivers believe that most people won’t know they are being screened as they might think this was just part of the medical history taken. [care partner representative perspective] On the other hand, some coming to the ED > 65 might be offended at certain screening and think there is ageism in health care.[^2,51–55^](https://paperpile.com/c/6qpfgj/9SzrO+T37S0+UJfKl+efQRL+OhJAX+rmhvM)  Older adults value function more than mortality,[^56^](https://paperpile.com/c/6qpfgj/zcdNa) and therefore would likely care about a disease that is associated with a high threat to functional independence.  **Overall**  Delirium risk stratification was identified as a high priority research focus by the GEAR Network, which included 49 transdisciplinary stakeholders including emergency physicians, geriatricians, nurses, social workers, pharmacists, and patient advocates.[^2^](https://paperpile.com/c/6qpfgj/9SzrO) |
| **Balance of effects** Does the balance between desirable and undesirable effects favor the intervention (screen for high-risk for delirium) or the comparison (status quo - not screen anybody for delirium)? | | |
| **Judgement** | **Research evidence** | **Additional considerations** |
| ○ Favors the comparison  ○ Probably favors the comparison  ○ Does not favor either the intervention or the comparison  ○ Probably favors the intervention  ○ Favors the intervention  ○ Varies  X Don't know | No direct or indirect evidence implementing delirium risk stratification approaches into practice exists to objectively assess the balance of desirable and undesirable effects of this approach to delirium management. | Confident understanding of the balance of desirable and undesirable effects for delirium risk stratification will require either pre- and post- studies or controlled trials that objectively evaluate the balance of beneficial effects of the delirium risk stratification and attributable associated adverse consequences.[^46^](https://paperpile.com/c/6qpfgj/KNDVd) |
| **Resources required** How large are the resource requirements (costs)? | | |
| **Judgement** | **Research evidence** | **Additional considerations** |
| ○ Large costs  ○ Moderate costs  ○ Negligible costs and savings  ○ Moderate savings  ○ Large savings  ○ Varies  X Don't know | No direct or indirect evidence explored or quantified the resource requirements for delirium risk stratification approaches. | Resources theoretically would be the cost of Electronic Health Record (EHR), training workforce, and monitoring. |
| **Certainty of evidence of required resources** What is the certainty of the evidence of resource requirements (costs)? | | |
| **Judgement** | **Research evidence** | **Additional considerations** |
| ○ Very low  ○ Low  ○ Moderate  ○ High  X No included studies | No direct or indirect evidence explored or quantified the resource requirements for delirium risk stratification approaches. | If efficacious delirium risk stratification strategies are identified for which the balance of anticipated benefits and harms favors the intervention, cost-benefit analyses would facilitate widespread implementation.[^57^](https://paperpile.com/c/6qpfgj/CmrKT) |
| **Cost effectiveness** Does the cost-effectiveness of the intervention favor the intervention (screen for high-risk for delirium) or the comparison (status quo - no delirium screening)? | | |
| **Judgement** | **Research evidence** | **Additional considerations** |
| ○ Favors the comparison  ○ Probably favors the comparison  ○ Does not favor either the intervention or the comparison  ○ Probably favors the intervention  ○ Favors the intervention  ○ Varies  X No included studies | No direct or indirect evidence of cost effectiveness of delirium screening or using risk factors to target delirium screening in the ED was identified. | Indirect evidence of multicomponent interventions, some in the ED and some in other settings, and some of which included screening in the intervention, demonstrate cost savings or no increase in costs compared to usual care.  1-Cost, but not cost-effectiveness: cost per problem identified in the ED – definite delirium $44.13, probable delirium $24.39, possible delirium $11.58. Of note, delirium was the most expensive geriatric syndrome to recognize.[^58^](https://paperpile.com/c/6qpfgj/Oh2UV)  2-GEDI-WISE program: various interventions including delirium screening in the ED, overall demonstrated “Per beneficiary, these savings were as much as $2905 after 30 days and $3202 after 60 days of the index ED visit”[^59^](https://paperpile.com/c/6qpfgj/bOLKG)  Missed detection of delirium by ED clinician had highest mortality over 6 months (30.8%). Mortality of delirious individuals detected was similar to non delirious individuals (11.8 vs 14.3). [^18^](https://paperpile.com/c/6qpfgj/rimA8)  Hustey noted recognition of 16% of those with delirium/altered mental status. When the ED physician was informed of the missed delirium, it did not alter their management of any of the cases. Five (5/19 = 26% of those with delirium) were discharged home with delirium and 4/5 returned within four days.[^19^](https://paperpile.com/c/6qpfgj/JHQUF) |
| **Equity** What would be the impact on health equity if we screen patients for high-risk delirium? | | |
| **Judgement** | **Research evidence** | **Additional considerations** |
| ○ Reduced  ○ Probably reduced  ○ Probably no impact  ○ Probably increased  ○ Increased  ○ Varies  X Don't know | No studies focused on the direct impact of using risk stratification for targeted screening for delirium on health care disparities. | **Non-English Speaking**  Patients with delirium and a non-English preferred language had an increased risk of receiving a physical restraint (RR 2.61, 95% CI 1.40-4.85) compared to patients who preferred English.[^60^](https://paperpile.com/c/6qpfgj/vxAG2) They also had an increased risk of receiving orders for new antipsychotic medications (RR 1.50, 95% CI 1.03–2.19).[^60^](https://paperpile.com/c/6qpfgj/vxAG2)  **Social Determinants of Health**  Area deprivation index was associated with delirium incidence and severity of delirium: “Neighborhood-level makers of social disadvantage are associated with delirium incidence and severity, and demonstrated an exposure-response relationship.”[^61^](https://paperpile.com/c/6qpfgj/pD5sD)  Varied evidence on social determinants of health (SDOH) demographic risk factors for delirium reported: “There have been some disagreeing viewpoints among researchers when studying the effect of demographic factors and SDOH on delirium risk[^62,63^](https://paperpile.com/c/6qpfgj/jCpfU+uOqRQ). Two separate studies investigating delirium risk in medical ICU patients in a Dutch region, and[^64^](https://paperpile.com/c/6qpfgj/5WnbG) surgical ICU patients in the US[^62^](https://paperpile.com/c/6qpfgj/jCpfU) found no significant effect of race or SDOH on delirium risk. In contrast, Dicks et al[^63^](https://paperpile.com/c/6qpfgj/uOqRQ). reported a substantial difference in delirium risk between non-Hispanic White and Black patients in the age groups[^65^](https://paperpile.com/c/6qpfgj/Q6379). These factors may need to be weighed in for a future design and application of risk stratification tools.  The impact of area deprivation index on delirium risk stratification approaches is unknown since these factors have not been the focus of or incorporated into existing research. Whether differences in area deprivation index or SDOH increase or decrease health disparities when applying delirium risk stratification is unknown. Further research is necessary to address this population.  **Delirium Superimposed on Dementia**  Patients with dementia may be a more vulnerable subset of delirium cases: “Identifying delirium in the presence of dementia, also described as delirium superimposed on dementia (DSD), is particularly challenging, as symptoms of delirium such as inattention, cognitive dysfunction, and altered level of consciousness, are also features of dementia. Because DSD is associated with poorer clinical outcomes than dementia alone, detecting delirium is important for reducing morbidity and mortality in this population.”[^66^](https://paperpile.com/c/6qpfgj/ITOzE) The risk of dementia associated with delirium is an important consideration, and caution is advised if a risk stratification does not include dementia.  Additionally, persons living with dementia tend to not be enrolled in ED delirium research due to concerts related to consent, and we cannot be certain whether this increases or decreases health inequities when deploying delirium risk stratification approaches in this population. Further research is necessary to address this population.  **Rurality**  Geriatric emergency medicine research has not historically been transparent about the inclusion or exclusion of rural patients in delirium research.[^67^](https://paperpile.com/c/6qpfgj/RJW41) When designing or implementing delirium risk stratification protocols for less resourced rural EDs, access to EHRs may increase health inequities.[^68^](https://paperpile.com/c/6qpfgj/ziZzL)  Overall research exploring the health equity implications of ED delirium risk stratification for subsets of gender/sex, ethnic/racial, rural, and social determinants of health strata are lacking.[^67^](https://paperpile.com/c/6qpfgj/RJW41) |
| **Acceptability** Is the intervention acceptable to key stakeholders? | | |
| **Judgement** | **Research evidence** | **Additional considerations** |
| ○ No  ○ Probably no  x Probably yes  ○ Yes  ○ Varies ○ Don't know | No direct evidence quantified the patient- or clinician-acceptability for ED delirium risk stratification approaches. | The 52 Geriatric Emergency Care Applied Research Network stakeholders including multidisciplinary clinicians, ED nurses, patients and patient surrogates prioritized research to identify pragmatic delirium risk stratification approaches, which implies that if such approaches could be derived, validated, and efficiently implemented they would be acceptable.[^2^](https://paperpile.com/c/6qpfgj/9SzrO) However, given that the participants of this study were not randomly selected, the results of this study may not be adequate to inform our understanding of acceptability as the sample is not unbiased or adequately representative of the stakeholders.  Focusing the delirium screening on those who are high-risk only would decrease the total screening burden which is possibly acceptable to patients/caregivers.  The acceptability is under assumption that delirium screening and automated process is not dependent on extra effort by nurse or physician. |
| **Feasibility** Is the intervention feasible to implement? | | |
| **Judgement** | **Research evidence** | **Additional considerations** |
| ○ No  ○ Probably no  ○ Probably yes  ○ Yes  ○ Varies  X Don't know | No direct or indirect evidence evaluated the feasibility for ED delirium risk stratification approaches. | Leveraging EHR and/or artificial intelligence to risk stratify for delirium may ultimately accelerate widespread implementation via enhanced feasibility. [^2,8,69,70^](https://paperpile.com/c/6qpfgj/HzrvM+9SzrO+qgnoA+5l1lo)  Otherwise, clinicians or nursing who are tasked to screen for delirium might appreciate the risk stratification tools to reduce their task burden.[^45,71^](https://paperpile.com/c/6qpfgj/nbrQv+k36K1) |

# **Summary of judgements**

|  | **Judgement** | | | | | | |
| --- | --- | --- | --- | --- | --- | --- | --- |
| **Problem** | No | Probably no | Probably yes | **Yes** |  | Varies | Don't know |
| **Test accuracy** | Very inaccurate | Inaccurate | Accurate | Very accurate |  | **Varies** | Don't know |
| **Desirable Effects** | Trivial | Small | **Moderate** | Large |  | Varies | Don't know |
| **Undesirable Effects** | Large | Moderate | Small | Trivial |  | Varies | **Don't know** |
| **Certainty of the evidence of test accuracy** | **Very low** | Low | Moderate | High |  |  | No included studies |
| **Certainty of the evidence of test's effects** | Very low | Low | Moderate | High |  |  | **No included studies** |
| **Certainty of the evidence of management's effects** | **Very low** | Low | Moderate | High |  |  | No included studies |
| **Certainty of the evidence of test result/management** | Very low | Low | Moderate | High |  |  | **No included studies** |
| **Certainty of effects** | Very low | Low | Moderate | High |  |  | **No included studies** |
| **Values** | Important uncertainty or variability | **Possibly important uncertainty or variability** | Probably no important uncertainty or variability | No important uncertainty or variability |  |  |  |
| **Balance of effects** | Favors the comparison | Probably favors the comparison | Does not favor either the intervention or the comparison | Probably favors the intervention | Favors the intervention | Varies | **Don't know** |
| **Resources required** | Large costs | Moderate costs | Negligible costs and savings | Moderate savings | Large savings | Varies | **Don't know** |
| **Certainty of evidence of required resources** | Very low | Low | Moderate | High |  |  | **No included studies** |
| **Cost effectiveness** | Favors the comparison | Probably favors the comparison | Does not favor either the intervention or the comparison | Probably favors the intervention | Favors the intervention | Varies | **No included studies** |
| **Equity** | Reduced | Probably reduced | Probably no impact | Probably increased | Increased | Varies | **Don't know** |
| **Acceptability** | No | Probably no | **Probably yes** | Yes |  | Varies | Don't know |
| **Feasibility** | No | Probably no | Probably yes | Yes |  | Varies | **Don't know** |

# **Type of recommendation 1: Regarding the Delirium Risk Score**

| Strong recommendation against the intervention | Conditional recommendation against the intervention | Conditional recommendation for either the intervention or the comparison | Conditional recommendation for the intervention | Strong recommendation for the intervention |
| --- | --- | --- | --- | --- |
| ○ | ○ | ○ | x | ○ |

**Type of recommendation 2: Regarding the REDEEM Score**

| Strong recommendation against the intervention | Conditional recommendation against the intervention | Conditional recommendation for either the intervention or the comparison | Conditional recommendation for the intervention | Strong recommendation for the intervention |
| --- | --- | --- | --- | --- |
| ○ | ○ | ○ | x | ○ |

# **Conclusions**

| **Recommendation** |
| --- |
| Recommendation #1: In older adults presenting to the ED, the Delirium Risk Score may be used to identify subsets of older adults at low risk for delirium. (Conditional recommendation, FOR) [Very low certainty of evidence]  Recommendation #2: In adults over age 65 admitted to the ED observation unit from the ED, Zucchelli’s risk assessment tool with score threshold of 4 may be used to identify those at low or high risk of delirium. (Conditional recommendation, FOR) [Very low certainty of evidence]  Recommendation #3: In adults over age 75 presenting to the ED, REDEEM Score threshold of 11 may be used to identify those at low or high risk of delirium. The low-risk cutoff of 5 may be used to rule out high risk for delirium. (Conditional recommendation, FOR) [Very low certainty of evidence] |
|  |
| **Justification** |
| The quantity and quality of direct and indirect evidence regarding targeted screening for delirium in the ED is lacking. Direct evidence is sparse, explores different approaches to delirium risk stratification, is at high risk of bias with respect to subject selection, and does not evaluate patient-oriented outcomes. However, acknowledging the morbidity and mortality associated with ED delirium, establishing pragmatic delirium risk stratification approaches within the time and resource constraints of the ED will likely improve scales of efficiency for recognizing, preventing, and treating delirium. |

| **Subgroup considerations** |
| --- |
| No specific subgroups were identified *a priori*. Patients with dementia are at higher risk for delirium, as noted above, and warrant a thorough assessment, such as an administration of delirium screening and serial assessments over time. |
| **Implementation considerations** |
| Implementation approaches for delirium risk stratification should incorporate implementation toolbox, education, and local opinion leader(s), while maintaining awareness of staff time and EHR costs or delays. More efficient screening of high risk patients may inadvertently bias against some patients who may have delirium. Furthermore, we need to identify evidence that anything we do with delirium care improves outcomes. Increased recognition of delirium may increase hospital admission further straining hospital capacity. |

| **Monitoring and evaluation** |
| --- |
| No recommendation for monitoring or evaluation until higher quantity and quality of evidence is available. |
| **Research priorities** |
| Resource priorities include explanatory and pragmatic multi-center studies with different resource capacities and diverse patient populations exploring the impact of delirium risk stratification on patient-centered and hospital outcomes, as well as on operational efficiency. If these explanatory and pragmatic multi-center studies show positive patient-centered outcomes, hybrid effectiveness implementation trials evaluating strategies to introduce delirium risk assessment approaches into diverse settings will inform pathways to scale up this approach to delirium management.  Research examining potential health disparities are needed for rural settings and patients who speak a language discordant from the examiner as well as those who have chronic cognitive impairment or who do not have caregivers with them and have no collateral information.  Substantial knowledge gaps persist for delirium risk stratification around the cost-effectiveness of various strategies (nurse- or clinician- administered instrument vs. EHR AI algorithm), patient values and what matters most around screening priorities, and components of ED and inpatient clinician acceptability. |

**References**

1. [Lee S, Chen H, Hibino S, et al. Can we improve delirium prevention and treatment in the emergency department? A systematic review. *J Am Geriatr Soc*. 2022;70(6):1838-1849.](http://paperpile.com/b/6qpfgj/6ef68)

2. [Carpenter CR, Hammouda N, Linton EA, et al. Delirium prevention, detection, and treatment in emergency medicine settings: A Geriatric Emergency care Applied Research (GEAR) Network scoping review and consensus statement. *Acad Emerg Med*. 2021;28(1):19-35.](http://paperpile.com/b/6qpfgj/9SzrO)

3. [Carpenter CR, Lee S, Kennedy M, et al. Delirium detection in the emergency department: A diagnostic accuracy meta-analysis of history, physical examination, laboratory tests, and screening instruments. *Acad Emerg Med*. 2024;31(10):1014-1036.](http://paperpile.com/b/6qpfgj/jWNOV)

4. [Smith HAB, Han JH, Ely EW. Meeting the challenges of delirium assessment across the aging spectrum. *Crit Care Med*. 2016;44(9):1775-1777.](http://paperpile.com/b/6qpfgj/NAIKg)

5. [Chary AN, Castilla-Ojo N, Joshi C, et al. Evaluating older adults with cognitive dysfunction: A qualitative study with emergency clinicians. *J Am Geriatr Soc*. 2022;70(2):341-351.](http://paperpile.com/b/6qpfgj/S3ztw)

6. [Shih RD, Carpenter CR, Tolia V, Binder EF, Ouslander JG. Balancing vision with pragmatism: The geriatric emergency department guidelines-realistic expectations from emergency medicine and geriatric medicine. *J Am Geriatr Soc*. 2022;70(5):1368-1373.](http://paperpile.com/b/6qpfgj/Ya11E)

7. [Chary A, Liu SW, Santangelo I, et al. A qualitative study of emergency department delirium prevention initiatives. *Delirium Commun*. 2022;2022. doi:](http://paperpile.com/b/6qpfgj/ctokE)[10.56392/001c.55690](http://dx.doi.org/10.56392/001c.55690)

8. [Chary AN, Brickhouse E, Torres B, et al. Leveraging the electronic health record to implement emergency department delirium screening. *Appl Clin Inform*. 2023;14(3):478-486.](http://paperpile.com/b/6qpfgj/5l1lo)

9. [Chary AN, Bhananker AR, Brickhouse E, et al. Implementation of delirium screening in the emergency department: A qualitative study with early adopters. *J Am Geriatr Soc*. 2024;72(12):3753-3762.](http://paperpile.com/b/6qpfgj/GmSAc)

10. [Pallin DJ, Allen MB, Espinola JA, Camargo CA Jr, Bohan JS. Population aging and emergency departments: visits will not increase, lengths-of-stay and hospitalizations will. *Health Aff (Millwood)*. 2013;32(7):1306-1312.](http://paperpile.com/b/6qpfgj/MhNwl)

11. [Salvi F, Morichi V, Grilli A, Giorgi R, De Tommaso G, Dessì-Fulgheri P. The elderly in the emergency department: a critical review of problems and solutions. *Intern Emerg Med*. 2007;2(4):292-301.](http://paperpile.com/b/6qpfgj/R1tBt)

12. [van Loveren K, Singla A, Sinvani L, et al. Increased emergency department hallway length of stay is associated with development of delirium. *West J Emerg Med*. 2021;22(3):726-735.](http://paperpile.com/b/6qpfgj/PUMbU)

13. [Moura Junior V, Westover MB, Li F, et al. Hospital complications among older adults: Better processes could reduce the risk of delirium. *Health Serv Manage Res*. 2022;35(3):154-163.](http://paperpile.com/b/6qpfgj/rEjwf)

14. [Devlin JW, Skrobik Y, Gélinas C, et al. Executive summary: Clinical practice guidelines for the prevention and management of pain, agitation/sedation, delirium, immobility, and sleep disruption in adult patients in the ICU. *Crit Care Med*. 2018;46(9):1532-1548.](http://paperpile.com/b/6qpfgj/D2Jgu)

15. [Grover S, Avasthi A. Clinical practice guidelines for management of delirium in elderly. *Indian J Psychiatry*. 2018;60(Suppl 3):S329-S340.](http://paperpile.com/b/6qpfgj/XzEba)

16. [*Delirium: Prevention, Diagnosis and Management in Hospital and Long-Term Care*. National Institute for Health and Care Excellence (NICE); 2023.](http://paperpile.com/b/6qpfgj/sHaXF)

17. [Lewis LM, Miller DK, Morley JE, Nork MJ, Lasater LC. Unrecognized delirium in ED geriatric patients. *Am J Emerg Med*. 1995;13(2):142-145.](http://paperpile.com/b/6qpfgj/jwI9n)

18. [Kakuma R, du Fort GG, Arsenault L, et al. Delirium in older emergency department patients discharged home: effect on survival. *J Am Geriatr Soc*. 2003;51(4):443-450.](http://paperpile.com/b/6qpfgj/rimA8)

19. [Hustey FM, Meldon SW, Smith MD, Lex CK. The effect of mental status screening on the care of elderly emergency department patients. *Ann Emerg Med*. 2003;41(5):678-684.](http://paperpile.com/b/6qpfgj/JHQUF)

20. [Lee JS, Tong T, Chignell M, et al. Prevalence, management and outcomes of unrecognized delirium in a National Sample of 1,493 older emergency department patients: how many were sent home and what happened to them? *Age Ageing*. 2022;51(2). doi:](http://paperpile.com/b/6qpfgj/VlXmK)[10.1093/ageing/afab214](http://dx.doi.org/10.1093/ageing/afab214)

21. [Howick AS, Thao P, Boie MA, Maia IW, Mullan AF, Bower SM, Palmer AK, Carpenter CR, Jeffery MM, Bellolio F. Evaluating Outcomes and Healthcare Utillization in Older Adults with Delirium Discharged from the Emergency Department. *Ann Emerg Med*. (In press).](http://paperpile.com/b/6qpfgj/gCBAE)

22. [Seidenfeld J, Lee S, Ragsdale L, Nickel CH, Liu SW, Kennedy M. Risk factors and risk stratification approaches for delirium screening: A Geriatric Emergency Department Guidelines 2.0 systematic review. *Acad Emerg Med*. 2024;31(10):969-984.](http://paperpile.com/b/6qpfgj/fhVvn)

23. [Rizzi MA, Torres Bonafonte OH, Alquezar A, et al. Prognostic value and risk factors of delirium in emergency patients with decompensated heart failure. *J Am Med Dir Assoc*. 2015;16(9):799.e1-e6.](http://paperpile.com/b/6qpfgj/PNjHa)

24. [Nguyen PVQ, Pelletier L, Payot I, Latour J. The Delirium Drug Scale is associated to delirium incidence in the emergency department. *Int Psychogeriatr*. 2018;30(4):503-510.](http://paperpile.com/b/6qpfgj/NB3GJ)

25. [Ryan S, Cooney E, Sayers K, et al. 262 training and use of the 4AT by a multi-disciplinary team to screen for delirium in the frail adult. *Age Ageing*. 2019;48(Supplement_3):iii17-iii65.](http://paperpile.com/b/6qpfgj/suUPT)

26. [Zucchelli A, Apuzzo R, Paolillo C, et al. Development and validation of a delirium risk assessment tool in older patients admitted to the Emergency Department Observation Unit. *Aging Clin Exp Res*. 2021;33(10):2753-2758.](http://paperpile.com/b/6qpfgj/NyrS4)

27. [Béland E, Nadeau A, Carmichael PH, et al. Predictors of delirium in older patients at the emergency department: a prospective multicentre derivation study. *CJEM*. 2021;23(3):330-336.](http://paperpile.com/b/6qpfgj/QyjO1)

28. [Émond M, Grenier D, Morin J, et al. Emergency Department stay associated delirium in older patients. *Can Geriatr J*. 2017;20(1):10-14.](http://paperpile.com/b/6qpfgj/1WkO6)

29. [Lee S, Harland K, Mohr NM, et al. Evaluation of emergency department derived delirium prediction models using a hospital-wide cohort. *J Psychosom Res*. 2019;127(109850):109850.](http://paperpile.com/b/6qpfgj/f1q3P)

30. [Silva L, Stanich JA, Jeffery MM. REcognizing Delirium in geriatric emergency medicine: the REDEEM risk stratification score. *Acad Emerg Med*. 2022;29(4):476-485.](http://paperpile.com/b/6qpfgj/QN6n1)

31. [Inouye SK, Viscoli CM, Horwitz RI, Hurst LD, Tinetti ME. A predictive model for delirium in hospitalized elderly medical patients based on admission characteristics. *Ann Intern Med*. 1993;119(6):474-481.](http://paperpile.com/b/6qpfgj/bRrHG)

32. [Chen Y, Du H, Wei BH, Chang XN, Dong CM. Development and validation of risk-stratification delirium prediction model for critically ill patients: A prospective, observational, single-center study. *Medicine (Baltimore)*. 2017;96(29):e7543.](http://paperpile.com/b/6qpfgj/KLWhz)

33. [Ragheb J, Norcott A, Benn L, et al. Barriers to delirium screening and management during hospital admission: a qualitative analysis of inpatient nursing perspectives. *BMC Health Serv Res*. 2023;23(1):712.](http://paperpile.com/b/6qpfgj/2Mydk)

34. [Chary AN, Lesser A, Inouye SK, Carpenter CR, Stuck AR, Kennedy M. A survey of delirium self-reported knowledge and practices among emergency physicians in the United States. *J Geriatr Emerg Med*. 2021;2(12). doi:](http://paperpile.com/b/6qpfgj/uPf5j)[10.17294/2694-4715.1010](http://dx.doi.org/10.17294/2694-4715.1010)

35. [Chary A, Joshi C, Castilla-Ojo N, et al. Emergency clinicians’ perceptions of communication tools to establish the mental baseline of older adults: A Qualitative study. *Cureus*. 2021;13(12):e20616.](http://paperpile.com/b/6qpfgj/g3Amm)

36. [Duggan MC, Van J, Ely EW. Delirium assessment in critically ill older adults: Considerations during the COVID-19 pandemic. *Crit Care Clin*. 2021;37(1):175-190.](http://paperpile.com/b/6qpfgj/uo8Zy)

37. [Reeves RR, Parker JD, Burke RS, Hart RH. Inappropriate psychiatric admission of elderly patients with unrecognized delirium. *South Med J*. 2010;103(2):111-115.](http://paperpile.com/b/6qpfgj/UpvCt)

38. [Han JH, Bryce SN, Ely EW, et al. The effect of cognitive impairment on the accuracy of the presenting complaint and discharge instruction comprehension in older emergency department patients. *Ann Emerg Med*. 2011;57(6):662-671.e2.](http://paperpile.com/b/6qpfgj/lD1rU)

39. [Han JH, Zimmerman EE, Cutler N, et al. Delirium in older emergency department patients: recognition, risk factors, and psychomotor subtypes. *Acad Emerg Med*. 2009;16(3):193-200.](http://paperpile.com/b/6qpfgj/n95pU)

40. [Kennedy M, Enander RA, Tadiri SP, Wolfe RE, Shapiro NI, Marcantonio ER. Delirium risk prediction, healthcare use and mortality of elderly adults in the emergency department. *J Am Geriatr Soc*. 2014;62(3):462-469.](http://paperpile.com/b/6qpfgj/mbpF0)

41. [Evensen S, Saltvedt I, Ranhoff AH, et al. Delirium and cognitive impairment among older patients in Norwegian emergency departments. *Tidsskr Nor Laegeforen*. 2019;139(6). doi:](http://paperpile.com/b/6qpfgj/exmPk)[10.4045/tidsskr.18.0578](http://dx.doi.org/10.4045/tidsskr.18.0578)

42. [Kennedy M, Helfand BKI, Gou RY, et al. Delirium in older patients with COVID-19 presenting to the emergency department. *JAMA Netw Open*. 2020;3(11):e2029540.](http://paperpile.com/b/6qpfgj/e67ly)

43. [Elmstahl S, Wahlfried C, Jerntorp P. Precipitating and predisposing factors of acute confusional state among emergency department patients. *Int Psychogeriatr*. 1995;7(4):519-526.](http://paperpile.com/b/6qpfgj/7qeto)

44. [Kohn MA, Carpenter CR, Newman TB. Understanding the direction of bias in studies of diagnostic test accuracy. *Acad Emerg Med*. 2013;20(11):1194-1206.](http://paperpile.com/b/6qpfgj/yZVKw)

45. [Oliveira J E Silva L, Berning MJ, Stanich JA, et al. Risk factors for delirium in older adults in the emergency department: A systematic review and meta-analysis. *Ann Emerg Med*. 2021;78(4):549-565.](http://paperpile.com/b/6qpfgj/k36K1)

46. [El Dib R, Tikkinen KAO, Akl EA, et al. Systematic survey of randomized trials evaluating the impact of alternative diagnostic strategies on patient-important outcomes. *J Clin Epidemiol*. 2017;84:61-69.](http://paperpile.com/b/6qpfgj/KNDVd)

47. [Grossmann FF, Hasemann W, Graber A, Bingisser R, Kressig RW, Nickel CH. Screening, detection and management of delirium in the emergency department - a pilot study on the feasibility of a new algorithm for use in older emergency department patients: the modified Confusion Assessment Method for the Emergency Department (mCAM-ED). *Scand J Trauma Resusc Emerg Med*. 2014;22(1):19.](http://paperpile.com/b/6qpfgj/qtdDx)

48. [Eagles D, Cheung WJ, Avlijas T, et al. Barriers and facilitators to nursing delirium screening in older emergency patients: a qualitative study using the theoretical domains framework. *Age Ageing*. 2022;51(1). doi:](http://paperpile.com/b/6qpfgj/tNx5X)[10.1093/ageing/afab256](http://dx.doi.org/10.1093/ageing/afab256)

49. [Hustey FM, Meldon SW. The prevalence and documentation of impaired mental status in elderly emergency department patients. *Ann Emerg Med*. 2002;39(3):248-253.](http://paperpile.com/b/6qpfgj/NVOP5)

50. [CMS Age Friendly Hospital Measure. ACS. 2024. Accessed February 14, 2025.](http://paperpile.com/b/6qpfgj/Fhoca) <https://www.facs.org/quality-programs/accreditation-and-verification/geriatric-surgery-verification/cms-age-friendly-hospital-measure/>

51. [Carpenter CR, Leggett J, Bellolio F, et al. Emergency department communication in persons living with dementia and care partners: A scoping review. *J Am Med Dir Assoc*. 2022;23(8):1313.e15-e1313.e46.](http://paperpile.com/b/6qpfgj/T37S0)

52. [Nowroozpoor A, Dussetschleger J, Perry W, et al. Detecting cognitive impairment and dementia in the emergency department: A scoping review. *J Am Med Dir Assoc*. 2022;23(8):1314.e31-e1314.e88.](http://paperpile.com/b/6qpfgj/UJfKl)

53. [Dresden SM, Taylor Z, Serina P, et al. Optimal emergency department care practices for persons living with dementia: A scoping review. *J Am Med Dir Assoc*. 2022;23(8):1314.e1-e1314.e29.](http://paperpile.com/b/6qpfgj/efQRL)

54. [Chera T, Tinetti M, Travers J, et al. “What Matters” in the emergency department: A prospective analysis of older adults’ concerns and desired outcomes. *Med Care*. 2024;62(12 Suppl 1):S50-S56.](http://paperpile.com/b/6qpfgj/OhJAX)

55. [Cohen RG. Ageism in health care? Yep, it’s a thing. *The Boston globe*. April 28, 2022.](http://paperpile.com/b/6qpfgj/rmhvM)

56. [Steinhauser KE, Christakis NA, Clipp EC, McNeilly M, McIntyre L, Tulsky JA. Factors considered important at the end of life by patients, family, physicians, and other care providers. *JAMA*. 2000;284(19):2476-2482.](http://paperpile.com/b/6qpfgj/zcdNa)

57. [Carpenter CR, Southerland LT, Lucey BP, Prusaczyk B. Around the EQUATOR with clinician-scientists transdisciplinary aging research (Clin-STAR) principles: Implementation science challenges and opportunities. *J Am Geriatr Soc*. 2022;70(12):3620-3630.](http://paperpile.com/b/6qpfgj/CmrKT)

58. [Miller DK, Lewis LM, Nork MJ, Morley JE. Controlled trial of a geriatric case-finding and liaison service in an emergency department. *J Am Geriatr Soc*. 1996;44(5):513-520.](http://paperpile.com/b/6qpfgj/Oh2UV)

59. [Hwang U, Dresden SM, Vargas-Torres C, et al. Association of a geriatric emergency department innovation program with cost Outcomes among Medicare beneficiaries. *JAMA Netw Open*. 2021;4(3):e2037334.](http://paperpile.com/b/6qpfgj/bOLKG)

60. [Reppas-Rindlisbacher C, Shin S, Purohit U, et al. Association between non-English language and use of physical and chemical restraints among medical inpatients with delirium. *J Am Geriatr Soc*. 2022;70(12):3640-3643.](http://paperpile.com/b/6qpfgj/vxAG2)

61. [Arias F, Chen F, Fong TG, et al. Neighborhood-level social disadvantage and risk of delirium following major surgery. *J Am Geriatr Soc*. 2020;68(12):2863-2871.](http://paperpile.com/b/6qpfgj/pD5sD)

62. [Khan BA, Perkins A, Hui SL, et al. Relationship between African-American race and delirium in the ICU. *Crit Care Med*. 2016;44(9):1727-1734.](http://paperpile.com/b/6qpfgj/jCpfU)

63. [Dicks R, Choi J, Waszynski C, et al. Health disparities in delirium. *Innov Aging*. 2021;5(Supplement_1):147-147.](http://paperpile.com/b/6qpfgj/uOqRQ)

64. [Wu TT, Zegers M, Kooken R, et al. Social determinants of health and delirium occurrence and duration in critically ill adults. *Crit Care Explor*. 2021;3(9):e0532.](http://paperpile.com/b/6qpfgj/5WnbG)

65. [Tripathi S, Fritz BA, Avidan MS, Chen Y, King CR. Algorithmic bias in machine learning based delirium prediction. *arXiv [csLG]*. Published online 2022. doi:](http://paperpile.com/b/6qpfgj/Q6379)[10.48550/ARXIV.2211.04442](http://dx.doi.org/10.48550/ARXIV.2211.04442)

66. [Fong TG, Hshieh TT, Tabloski PA, et al. Identifying delirium in persons with moderate or severe dementia: Review of challenges and an illustrative approach. *Am J Geriatr Psychiatry*. 2022;30(10):1067-1078.](http://paperpile.com/b/6qpfgj/ITOzE)

67. [Chary AN, Suh M, Ordoñez E, et al. A scoping review of geriatric emergency medicine research transparency in diversity, equity, and inclusion reporting. *J Am Geriatr Soc*. 2024;72(11):3551-3566.](http://paperpile.com/b/6qpfgj/RJW41)

68. [Anzalone AJ, Geary CR, Dai R, Watanabe-Galloway S, McClay JC, Campbell JR. Lower electronic health record adoption and interoperability in rural versus urban physician participants: a cross-sectional analysis from the CMS quality payment program. *BMC Health Serv Res*. 2025;25(1):128.](http://paperpile.com/b/6qpfgj/ziZzL)

69. [Pendlebury ST, Lovett NG, Smith SC, Wharton R, Rothwell PM. Delirium risk stratification in consecutive unselected admissions to acute medicine: validation of a susceptibility score based on factors identified externally in pooled data for use at entry to the acute care pathway. *Age Ageing*. 2017;46(2):226-231.](http://paperpile.com/b/6qpfgj/HzrvM)

70. [Southerland LT, Hunold KM, Van Fossen J, et al. An implementation science approach to geriatric screening in an emergency department. *J Am Geriatr Soc*. 2022;70(1):178-187.](http://paperpile.com/b/6qpfgj/qgnoA)

71. [Wong A, Young AT, Liang AS, Gonzales R, Douglas VC, Hadley D. Development and validation of an electronic health record-based machine learning model to estimate delirium risk in newly hospitalized patients without known cognitive impairment. *JAMA Netw Open*. 2018;1(4):e181018.](http://paperpile.com/b/6qpfgj/nbrQv)

# **Appendix S2. PICO2 Evidence to Decision document**

| **QUESTION** | |
| --- | --- |
| Can delirium screening instruments accurately rule in or rule out delirium in older emergency department patients? | |
| **POPULATION:** | Older adults with suspected incident or prevalent (Pre-ED) delirium during episode of emergency department (ED) care |
| **INTERVENTION:** | Delirium diagnosis using structured instrument or clinician gestalt |
| **PURPOSE OF THE TEST:** | Accurately and reliably distinguish presence or absence of delirium |
| **ROLE OF THE TEST:** | Facilitate feasible approaches to rule-in or rule-out of delirium in the ED setting |
| **LINKED TREATMENTS:** | Few interventions initiated in the ED were found to consistently reduce the incidence or duration of delirium. These include the use of melatonin, regional anesthesia for hip fracture, and avoidance of Foley catheters.[^1^](https://paperpile.com/c/aGrzfz/jJ5Gw) |
| **ANTICIPATED OUTCOMES:** | Reduced ED length of stay, preventable ED returns, delirium severity, delirium duration, or mortality. Reduced hospital length of stay. Reduced hospital admission rates. Reduced hospital incident delirium rates and pressure injuries. Patient-centered outcomes: reduced functional decline, reduced use of restraints and sedatives, reduced falls (ED or inpatient or home). |
| **SETTING:** | ED |
| **PERSPECTIVE:** | Patient, family or care partner, emergency medicine clinician, inpatient team, hospital administrators |
| **BACKGROUND:** | The American College of Emergency Physicians’, American Geriatrics Society, Society for Academic Emergency Medicine, and Emergency Nurses Association 2014 Geriatric Emergency Department Guidelines advocate for recognizing the presence of delirium.[^2^](https://paperpile.com/c/aGrzfz/3Olhh) The majority of ACEP Geriatric Emergency Department Accreditation (GEDA) Level 1 sites and nearly half of Level 2 sites report delirium detection protocols.[^3^](https://paperpile.com/c/aGrzfz/ZUKiA) Process quality indicators in ED settings for quantifiable assessment of higher quality delirium care exist and prioritize the recognition of incident or prevalent (Pre-ED)delirium.[^4,5^](https://paperpile.com/c/aGrzfz/f5qH1+BYxZm) Nonetheless, ED clinicians have identified multiple pragmatic barriers to routine delirium detection.[^6,7^](https://paperpile.com/c/aGrzfz/DulHv+SVlpC) Delirium can have significant negative psychological impacts on family caregivers, including anxiety, depression, fear, and uncertainty.[^8^](https://paperpile.com/c/aGrzfz/GEvpq) Caregivers often feel responsible for supporting their loved ones but perceive their role negatively due to increasing demands and lack of support.[^9^](https://paperpile.com/c/aGrzfz/7GnQQ) The onset of delirium, personality changes, and confusion contribute to caregivers' emotional exhaustion and distress, affecting the quality of their relationships with patients[^9^](https://paperpile.com/c/aGrzfz/7GnQQ) |
| **SUBGROUPS:** | Hypoactive delirium, hyperactive delirium, incident delirium, prevalent delirium, pre-existing cognitive impairment, LTCF/assisted living vs. community dwelling |
| **CONFLICT OF INTERESTS:** | **Christopher R. Carpenter, MD, MSc:**  Geriatric Emergency Care Applied Research (GEAR) Network Implementation Science Core Lead, Geriatric Emergency Department Collaborative co-Investigator, *Academic Emergency Medicine* Deputy Editor-in-Chief, Guidelines for Reasonable & Appropriate Care (GRACE) Lead, *Journal of the American Geriatrics Society* Associate Editor, Clinician-Scientists Transdisciplinary Aging Research Leadership Core, Geriatric Emergency Department Guidelines co-lead.  **Debra Eagles, MD, MSc:**  Nothing to disclose.  **Maura Kennedy, MD, MPH:** Serves on the American College of Emergency Physicians’ Geriatric Emergency Department Accreditation Board of Governors. Although a volunteer position, monetary remuneration is occasionally received on behalf of work done for the program.  **Sangil Lee MD, MS:** Nothing to disclose.  **Shan W. Liu, MD, SD:** Serves on the American College of Emergency Physicians’ Geriatric Emergency Department Accreditation Advisory Board, as well as the International Federation of Emergency Medicine Geriatric Emergency Medicine Special Interest Group. |

# **ASSESSMENT**

| **Problem** Is the problem a priority? | | |
| --- | --- | --- |
| **JUDGEMENT** | **RESEARCH EVIDENCE** | **ADDITIONAL CONSIDERATIONS** |
| ○ No ○ Probably no ○ Probably yes X Yes ○ Varies ○ Don't know | Delirium occurs in 6% to 38% of adults over age 65 when formal cognitive assessments occur.[^10^](https://paperpile.com/c/aGrzfz/AqCnh) ED nurses and physicians miss up to 70% of patients with delirium.[^11–13^](https://paperpile.com/c/aGrzfz/55uXg+fweGj+shvWi) Mortality at 6-months is adversely affected when patients are discharged home with delirium.[^14^](https://paperpile.com/c/aGrzfz/cy2C5) Even when recognized, the occurrence of delirium is associated with increased hospital length of stay, as well as accelerated progression of dementia, depression, and increased mortality.[^15–19^](https://paperpile.com/c/aGrzfz/Z4PCV+pzXFN+2iSlc+0AfrB+DpgeV) Clinicians may recognize that a patient has altered mental status, which is a constellation of symptoms, but not recognize the specific diagnosis of delirium.[^20^](https://paperpile.com/c/aGrzfz/DwcMv) | The GEAR Network identified 27 distinct delirium instruments evaluated in ED settings, so evaluating the comparative accuracy, reliability, feasibility, balance of desirable and undesirable effects, resources, and acceptability of these multiple instruments using a GRADE-based process is an unmet need.[^10^](https://paperpile.com/c/aGrzfz/AqCnh) |
| **Test accuracy** How accurate is the test? | | |
| **JUDGEMENT** | **RESEARCH EVIDENCE** | **ADDITIONAL CONSIDERATIONS** |
| ○ Very inaccurate ○ Inaccurate ○ Accurate ○ Very accurate X Varies ○ Don't know | **Clinical gestalt (4 studies)**  · ED clinicians accurately rule-in delirium (Likelihood Ratio positive [LR+] 21.8 [95% CI 4.5-104.9]), but do not accurately rule-out delirium[^21^](https://paperpile.com/c/aGrzfz/xRp5Y).    **4AT (3 studies)**[^22–24^](https://paperpile.com/c/aGrzfz/yqACa+BWkuo+uqH0t)  · Research assistant-administered accurately rules-in (LR+ 7.5 [95% CI 2.7-20.7]) and rules-out (Likelihood Ratio negative [LR-] 0.18 [95% CI 0.09-0.34]) delirium.[^21^](https://paperpile.com/c/aGrzfz/xRp5Y)    **AMT-4 (2 studies)**[^25,26^](https://paperpile.com/c/aGrzfz/pEZFm+1iOhL)  · Acceptable to rule in (LR+ 4.3 [95% CI 2.4-7.8]) but inaccurate to rule out (LR- 0.22 [95% CI 0.05-1] delirium.[^21^](https://paperpile.com/c/aGrzfz/xRp5Y)    **O3DY (1 study each evaluating single O3DY vs. serial O3DY)**  · Single O3DY inaccurate to rule-in (LR+ 1.6 [95% CI 1.2-1.7]) or rule-out (LR- 0.30 [95% CI 0.1-0.73]) delirium.  · Serial O3DY inaccurate to rule-in (LR+ 2.0 [95%CI 1.4-2.3] or rule-out (LR- 0.27 [95% CI 0.07-0.70]) delirium.[^21^](https://paperpile.com/c/aGrzfz/xRp5Y)    **bCAM (1 study)**  · ED clinicians accurately rule-in delirium (LR+ 19.9 [95% 12-33]) and rule-out (LR- 0.17 [95% CI 0.09-0.32) delirium.[^21^](https://paperpile.com/c/aGrzfz/xRp5Y)    **CAM-ICU (1 study)**  · ED clinicians accurately rule-in delirium (LR+ 51.3 [95% 21-125]) and to a lesser extent rule-out (LR- 0.28 [95% CI 0.18-0.44) delirium. [^21^](https://paperpile.com/c/aGrzfz/xRp5Y)  **Delirium Triage Screen (1 study)**  · ED clinicians inaccurate to rule-in delirium (LR+ 2.2 [95% CI 1.9-2.5]) but accurately rule-out delirium (LR- 0.04 [95% CI 0.01-0.25]).[^21^](https://paperpile.com/c/aGrzfz/xRp5Y)    **FAM-CAM (1 study)**[^27^](https://paperpile.com/c/aGrzfz/zUAyN)  · Used by untrained family members at ED bedside does not rule-in (LR+ 3.4 [95% CI 1.4-5.4]) or rule-out (LR- 0.5 [95% CI 0.3-0.7]) delirium.[^21^](https://paperpile.com/c/aGrzfz/xRp5Y)    **mCAM (1 study)**  · ED clinicians accurately rule-in (LR+ 39.9 [95% CI 19-60]) and rule-out (LR- 0.1 [95% CI 0.02-0.30]) delirium.[^21^](https://paperpile.com/c/aGrzfz/xRp5Y)    **NuDESC (1 study)**  · ED nurses accurately rule-in delirium (LR+ 7.4 [95% CI 5-11]) but less accurately rule-out delirium (LR- 0.37 [95% CI 0.25-0.53]).[^21^](https://paperpile.com/c/aGrzfz/xRp5Y)    **RASS (2 studies)**   - Han et al (n=406) evaluated the use of RASS score for delirium detection. ED physicians accurately ruled in (LR+ 5.5 [95% 4-7]) and ruled out (LR- 0.21 [95% CI 0.12-0.38]) delirium.[^21,28,29^](https://paperpile.com/c/aGrzfz/xRp5Y+Ep0ID+lxMjR) Furthermore, Grossman found that among 285 patients, LR+ 10.31 (95% CI 6.06; 17.51), LR- 0.32 (95% CI 0.16; 0.63) for mRASS other than 0 when tested by trained nurses.[^30^](https://paperpile.com/c/aGrzfz/ORy9o) | Few studies evaluated inter-rater agreement for individual delirium diagnosis instruments. Agreement between research assistants and ED physicians was good for DTS and bCAM (k = 0.79) and RASS (k = 0.63) and excellent for CAM-ICU (k = 0.92).[^31^](https://paperpile.com/c/aGrzfz/WNFq9)    The median time to obtain the b-CAM was 3 minutes compared with 0.5-2 minutes for the O3DY.[^10^](https://paperpile.com/c/aGrzfz/AqCnh)  The criterion standard to define the presence or absence of delirium ranged significantly across studies and few studies explicitly adhere to the EQUATOR Network STARD criteria for diagnostic accuracy.[^32^](https://paperpile.com/c/aGrzfz/rkc3Z)  The GED Guideline delirium group had long discussions about the likelihood ratio thresholds for inclusion of delirium detection instruments. While classic evidence-based medicine acknowledges LR+>10 or LR-<0.1 as the thresholds of relevance given their large effect on posttest probability, others recognize a role for lower thresholds. For example, an LR+ 2-5 or LR- 0.2-0.5 has a lesser (but present) effect on posttest probability.[^33^](https://paperpile.com/c/aGrzfz/qc2RW)  As for who conducts these screenings, the studies included a variable mixture of physicians, nurses, and research assistants.  **AMT-4:**  Arendts 2017:[^25,26^](https://paperpile.com/c/aGrzfz/pEZFm+1iOhL) RNs only; Dyer 2017:[^25,26^](https://paperpile.com/c/aGrzfz/pEZFm+1iOhL) research assistants only. physician accuracy was never assessed.  **mCAM**:  Hasemann 2018: RNs only. physician accuracy was not assessed.  **4AT and CAM:**  Shenkin 2019: RNs or graduate assistants only (not physicians); Gagne and O’Sullivan evaluated 4AT, but both used trained research assistants (not nurses or physicians). physician accuracy was not assessed.  Some studies imply comparability between administering and interpreting the accuracy of screening by physicians and research assistants:  **In a study by Han et al**[^34^](https://paperpile.com/c/aGrzfz/cyW8P):  The kappa between the EP and RAs in performing the DTS was 0.79 (95% CI: 0.73 – 0.85)  The kappa between the EP and RAs in performing the bCAM was 0.88 (95%CI: 0.81 – 0.95)  **In another study by Han et al**[^29^](https://paperpile.com/c/aGrzfz/lxMjR):  The kappa between the EP and RAs in performing the CAM-ICU was 0.92 (95% CI = 0.85 to 0.98)  There are also several studies from outside the ED on RNs administering these tools.  Therefore, it was not felt that the clinician type (physician vs RN) should be differentiated in the recommendations.(consensus based on information above) |
| **Desirable Effects** How substantial are the desirable anticipated effects?  In other words, if the desirable effects of screening for delirium were true, how substantial would they be? | | |
| **JUDGEMENT** | **RESEARCH EVIDENCE** | **ADDITIONAL CONSIDERATIONS** |
| X Trivial ○ Small ○ Moderate ○ Large ○ Varies ○ Don't know | No direct or indirect evidence implementing delirium identification approaches into practice exists to enable quantification of on patient-oriented or process-oriented desirable effects. | Implementing routine delirium diagnostic testing into clinical care is theoretically associated with substantial desirable effects. Indirect evidence implies that early delirium detection and intervention can reduce hospital length of stay by 2.27 days for patients with delirium and decrease delirium-related falls from 23.4% to 17%.[^35^](https://paperpile.com/c/aGrzfz/f5fbt) Effective delirium identification implementation strategies include addressing barriers, multifaceted training, and interdisciplinary communication.[^36^](https://paperpile.com/c/aGrzfz/aDUyX) Patients with delirium symptoms require twice the duration of delirium-specific care and have a 4.4 times higher fall risk compared to those without symptoms.[^37^](https://paperpile.com/c/aGrzfz/FImLQ) However, introducing widespread and uniform delirium detection initiatives face real-world challenges, as current practices often deviate from research methods and clinical practice guideline recommendations.[^38^](https://paperpile.com/c/aGrzfz/oIfOd) To overcome these obstacles, tailored implementation strategies and staff education are crucial.[^39,40^](https://paperpile.com/c/aGrzfz/mG8zm+eAoLK) Overall, routine delirium detection has the theoretical potential to significantly improve patient outcomes and safety in hospital settings, particularly for older and critically ill patients.  From a reimbursement standpoint, the ICD-9 code for AMS generally results in lower reimbursement compared to delirium or encephalopathy because AMS is considered a symptom complex rather than a specific diagnosis, while delirium and encephalopathy represent more severe, identifiable medical conditions. Moreover, “encephalopathy” codes are designated as major complication or comorbidity, whereas “delirium” codes are designated as complication or comorbidity and thus associated with a lower reimbursement and lesser impact on value metrics.[^41^](https://paperpile.com/c/aGrzfz/uhnsi)  Therefore, placing the diagnosis of “delirium” on the chart would improve the reimbursement for this condition and support the added costs of delirium diagnostic testing & management. |
| **Undesirable Effects** How substantial are the undesirable anticipated effects? | | |
| **JUDGEMENT** | **RESEARCH EVIDENCE** | **ADDITIONAL CONSIDERATIONS** |
| ○ Large ○ Moderate ○ Small ○ Trivial ○ Varies X Don't know | No direct or indirect evidence implementing delirium detection approaches into practice data/studies exist to enable quantification of patient-oriented or process-oriented undesirable effects. | **Time Constraints**  Although the proposed tests take 5 minutes or less, it is unclear if ED clinicians are willing to spend this much time on geriatric screening or interventions.; they might be willing to spend less than 2 minutes on this task.  Extrapolating from a survey of ED clinicians (n=102) regarding geriatric fall risk assessment and interventions, most were not willing to spend more than 5 minutes on risk assessment and prevention despite most (80%) acknowledging its importance. However, almost half (44%) are willing to spend up to 5 minutes on this task, and  almost all (90%) were willing to spend less than 2 minutes on this task,[^42^](https://paperpile.com/c/aGrzfz/FdV6K) highlighting the importance of the brevity of the chosen tool.  **Delirium Detection Obstacles**  Chary et al’s survey of ED physicians noted that among the 26% of respondents who reported nonadherence to a delirium screening protocol, suspected reasons for non-adherence were forgetting to apply protocol, time constraints, not being convinced the protocol is necessary, limited staff awareness about the protocol, and follow-up actions for a positive screen not being clear or feasible.[^43^](https://paperpile.com/c/aGrzfz/uH3DR)  False positive cases may lead to unnecessary testing, prolonged hospitalizations, and subsequently increased health care costs.[^44^](https://paperpile.com/c/aGrzfz/bqlnn)  Delirium detection may be resource intensive - note the resources box below for details.  **Downstream undesirable effects of missed delirium:**  Mortality at 6-months is adversely affected when patients are discharged home with delirium.[^14^](https://paperpile.com/c/aGrzfz/cy2C5)  Patients discharged home with delirium are likely to return to the ED (80%).[^45^](https://paperpile.com/c/aGrzfz/ROsyo)  Symptoms of delirium may be incorrectly attributed to psychiatric illness, especially in patients with a history of mental illness, with subsequent psychiatric admission rather than medical admission and therefore inadequate medical workup.[^46^](https://paperpile.com/c/aGrzfz/NslMb)  Delirium superimposed on dementia is associated with decreased accuracy of the older patient's presenting illness and decreased comprehension of ED discharge instructions.[^47^](https://paperpile.com/c/aGrzfz/pOr2V) |
| **Certainty of the evidence of test accuracy** What is the overall certainty of the evidence of test accuracy? | | |
| **JUDGEMENT** | **RESEARCH EVIDENCE** | **ADDITIONAL CONSIDERATIONS** |
| X Very low ○ Low ○ Moderate ○ High ○ No included studies | Generally, extremely wide confidence intervals with delirium detection diagnostic accuracy studies conducted in single academic research settings.  For example, the usual population with pre-test probability of 17%, where a positive screening test with +LR of 5 increased the post-probability to 51%. Using the 11% threshold per Carpenter et al., treatment may be indicated.[^21^](https://paperpile.com/c/aGrzfz/xRp5Y) On the other hand, the usual population with pre-test probability of 17%, where negative screening test with LR of 0.3 decreased the post-test probability to 6%. If the high-risk population with pre-test probability of 30%, where positive screening test with LR of 5 increased the post-test probability to 68%, this indicates the need for treatment. Lastly, the high-risk population with pre-test probability of 30%, where negative screening test with LR of 0.3 decreased the post-test probability to 11%, which is the cutoff for treatment, so serial monitoring is likely indicated. | None. |
| **Certainty of the evidence of test's effects** What is the overall certainty of the evidence for any critical or important direct benefits, adverse effects or burden of the test? | | |
| **JUDGEMENT** | **RESEARCH EVIDENCE** | **ADDITIONAL CONSIDERATIONS** |
| ○ Very low X Low ○ Moderate ○ High ○ No included studies | No direct or indirect evidence implementing delirium detection approaches into practice exists to quantify patient-oriented or process-oriented effects or the balance of benefits, adverse effects, or burden of the various delirium detection approaches. | A serious game was tested to assess cognitive status and delirium in the clinical setting.[^48^](https://paperpile.com/c/aGrzfz/tHbpl)  The delirium detection approaches have no “direct” benefit per se, but rather detection of delirium, which in turn impacts patient management and subsequent outcome, as detailed below.  There are no adverse effects other than those related to the burden of training to perform the test (fixed time & money investment), plus the burden of time to perform these screening tests.  The time for training is variable.  For example, the FAM-CAM requires one hour of staff training.[^49^](https://paperpile.com/c/aGrzfz/YNmeR)  The time-on-task required to detect delirium is variable, ranging from 10 seconds to 6.2 minutes, as detailed in the resource box. Shorter time frames (<1 minute) are preferable for emergency physicians. |
| **Certainty of the evidence of management's effects** What is the overall certainty of the evidence of effects of the management that is guided by the test results? | | |
| **JUDGEMENT** | **RESEARCH EVIDENCE** | **ADDITIONAL CONSIDERATIONS** |
| X Very low ○ Low ○ Moderate ○ High ○ No included studies | Only 10 studies have evaluated ED prevention of incident delirium or treatment of prevalent (Pre-ED) delirium. Three controlled (non-RCT) studies evaluated multifactorial delirium prevention. Three controlled (non-RCT) studies evaluated regional anesthesia for hip fracture. One case control study evaluated the association of foley catheters and delirium duration.[^1^](https://paperpile.com/c/aGrzfz/jJ5Gw)  One RCT demonstrated reduced incident delirium with the use of nocturnal melatonin (OR 0.19 [95% CI 0.06-0.62).    One multifactorial program reduced inpatient delirium prevalence from 41% to 19% and another multifactorial program reduced incident delirium (relative risk 0.37 [95% CI 0.22-0.61]).    The use of Foley catheters in the ED increased the duration of delirium (proportional odds ratio 3.1 [95% CI 1.3-7.4]). | A Cochrane review assessed the effectiveness of antipsychotic medications for treating delirium in non-ICU hospitalized patients and found no significant impact on the severity or duration of delirium.[^50^](https://paperpile.com/c/aGrzfz/Hc58E) Due to the scarcity of research within palliative care, the available evidence was insufficient to determine the effectiveness of pharmacological treatments for delirium in terminally ill individuals.[^51^](https://paperpile.com/c/aGrzfz/ZNHV0) Similarly, insufficient evidence was identified regarding the use of cholinesterase inhibitors for treating delirium in non-ICU hospital settings.[^52^](https://paperpile.com/c/aGrzfz/nrwsD) A review of prevention strategies in long-term care highlighted a lack of focus on delirium interventions in s EDs. [^53^](https://paperpile.com/c/aGrzfz/rvCpk) However, some interventions, such as a software-based system to identify high-risk medications and pharmacist-led medication reviews, showed promise in reducing delirium incidence.[^54^](https://paperpile.com/c/aGrzfz/c7Cla) Additionally, robust evidence supports the effectiveness of multicomponent delirium prevention programs in reducing its occurrence in non-ICU hospital settings. [^55^](https://paperpile.com/c/aGrzfz/MYOgQ)  Patients at high risk for delirium during diagnostic testing might benefit from preventative strategies, so a negative diagnostic test for prevalent (Pre-ED) delirium might be the most important. Thus, we need to consider separating prevalent (Pre-ED) and incident delirium. (Personal communication) |
| **Certainty of the evidence of test result/management** How certain is the link between test results and management decisions? | | |
| **JUDGEMENT** | **RESEARCH EVIDENCE** | **ADDITIONAL CONSIDERATIONS** |
| X Very low ○ Low ○ Moderate ○ High ○ No included studies | Single prospective study from two tertiary hospitals in Australia with sequential introduction of delirium screening (phase 1) and then delirium interventions (phase 2) did not demonstrate significantly different delirium detection or prevention rates.[^25^](https://paperpile.com/c/aGrzfz/pEZFm) | None. |
| **Certainty of effects** What is the overall certainty of the evidence of effects of the test? | | |
| **JUDGEMENT** | **RESEARCH EVIDENCE** | **ADDITIONAL CONSIDERATIONS** |
| X Very low ○ Low ○ Moderate ○ High ○ No included studies | Wide confidence intervals with studies conducted in academic medical centers and no consistent signal of benefits associated with ED delirium diagnosis and corresponding management. | None. |
| **Values** Is there important uncertainty about or variability in how much people value the main outcomes? | | |
| **JUDGEMENT** | **RESEARCH EVIDENCE** | **ADDITIONAL CONSIDERATIONS** |
| ○ Important uncertainty or variability X Possibly important uncertainty or variability ○ Probably no important uncertainty or variability ○ No important uncertainty or variability | Scant research of delirium patients, care partners, ED nurses, or ED physicians’ preferences to quantify the value placed on the identification of delirium. Regional and national qualitative research from the United States implies that ED physicians identify 5 major challenges when contemplating enhancing delirium detection across emergency medicine: availability of care partners, reliability of sources, language barriers, time constraints, and incomplete transfer documentation.[^6,43^](https://paperpile.com/c/aGrzfz/uH3DR+DulHv) | **Patients & Caregivers**  As opposed to evaluating every older adult in the ED for delirium, delirium detection efforts should focus on those with delirium risk factors and comorbidities as some coming to the ED > 65 are not frail or at risk for delirium and might be offended at certain a risk agnostic, one-size-fits-all delirium screening approach and view this unilateral approach as healthcare ageism.[^56^](https://paperpile.com/c/aGrzfz/gAjnZ)  Most people won’t know they are being evaluated for delirium as they might think this was just part of the medical history taken. [care partner representative perspective]  Patients value obtaining a diagnosis. In a study focusing on “what matters” to older ED adults (n=1013), 25.6% identified “obtaining a diagnosis” as their desired outcome.[^57^](https://paperpile.com/c/aGrzfz/g6QSd)  **Healthcare workers**  Nurse and physician barriers that impede effective delirium detection exist.[^58,59^](https://paperpile.com/c/aGrzfz/wsq0V+f5tgi) ED staff will need to focus on competing demands and prioritize other time-dependent emergencies, such as sepsis, stroke, and myocardial infarction for which the quality of care is currently being judged and publicly reported.  In a survey of 46 ED nurses at an academic medical center, 76.1% felt geriatric screening should be standard practice for all EDs.[^39^](https://paperpile.com/c/aGrzfz/mG8zm)  Currently, the link between delirium detection and downstream action to prevent or mitigate delirium in the ED is lacking.[^1^](https://paperpile.com/c/aGrzfz/jJ5Gw) In addition, with ED overcrowding, creating a quiet environment may not be feasible and therefore the detection of delirium may not lead to change in the care provided due to system-level factors.  **Health systems**  Healthcare administrators will need to prioritize myriad geriatric ED guideline recommendations (for delirium, elder abuse, medication safety, falls, dementia, frailty, and palliative care) with already often overloaded staff. Is any ED able to provide geriatric consultation or offer comprehensive geriatric assessment? |
| **Balance of effects** Does the balance between desirable and undesirable effects favor the intervention or the comparison? | | |
| **JUDGEMENT** | **RESEARCH EVIDENCE** | **ADDITIONAL CONSIDERATIONS** |
| ○ Favors the comparison ○ Probably favors the comparison ○ Does not favor either the intervention or the comparison ○ Probably favors the intervention ○ Favors the intervention ○ Varies X Don't know | Insufficient and inconsistent direct and indirect evidence quantifying the potential desirable or undesirable effects associated with ED delirium detection strategies exists. | None. |
| **Resources required** How large are the resource requirements (costs)? | | |
| **JUDGEMENT** | **RESEARCH EVIDENCE** | **ADDITIONAL CONSIDERATIONS** |
| ○ Large costs ○ Moderate costs ○ Negligible costs and savings ○ Moderate savings ○ Large savings X Varies ○Don't know | The following resource requirements and associated costs/revenue of routine ED delirium detection strategies have yet to be quantified but would include:    · Training requirements for skill acquisition and maintenance using the delirium diagnosis instruments individually or collectively.  · ED charges for delirium as a diagnosis.  · Costs associated with inpatient delirium care. | **Cost of administration:**  *Time:*  The required time is variable, as follows:   - Clinical gestalt: requires no additional time - 4AT: requires < 2-3 min to perform [^24,60^](https://paperpile.com/c/aGrzfz/uqH0t+imt9z) - AMT-4: requires less time than the 4AT - O3DY: requires < 2 min[^60^](https://paperpile.com/c/aGrzfz/imt9z) - bCAM: requires < 2 min[^61^](https://paperpile.com/c/aGrzfz/VU6zd) - CAM-ICU: requires 1-3 min [^62^](https://paperpile.com/c/aGrzfz/YGwdU) - DTS: requires 10-20 sec[^34^](https://paperpile.com/c/aGrzfz/cyW8P) - FAM-CAM: requires 5 min [^49^](https://paperpile.com/c/aGrzfz/YNmeR) - mCAM: requires 3.2-6.2 min [^63^](https://paperpile.com/c/aGrzfz/ZIeVu) - NuDESC: requires < 2 min [^64^](https://paperpile.com/c/aGrzfz/6alBg) - RASS: requires < 10 sec [^28^](https://paperpile.com/c/aGrzfz/Ep0ID)   *Monetary costs:*  Varies depending on the length and complexity of the test, as well as whether delirium detection approach relies upon physicians, nurses, or other healthcare professionals. Extrapolating from indirect evidence of general ED screening questions, implementing a 30 sec triage RN screen in an ED with an annual volume of approximately 80,000 patients is $20,000,[^65^](https://paperpile.com/c/aGrzfz/AadZ3) which equates to approximately 25 cents per patient.  An important indirect consequence of delirium detection strategies is the cost of not screening for alternative geriatric syndromes (e.g. frailty, falls, polypharmacy, elder abuse) due to time constraints in the contemporary ED.  **Cost of training:**  Largely unknown, and likely variable. For example, the FAM-CAM requires one hour of staff training.[^49^](https://paperpile.com/c/aGrzfz/YNmeR) |
| **Certainty of evidence of required resources** What is the certainty of the evidence of resource requirements (costs)? | | |
| **JUDGEMENT** | **RESEARCH EVIDENCE** | **ADDITIONAL CONSIDERATIONS** |
| ○ Very low X Low ○ Moderate ○ High ○ No included studies | No direct evidence explored or quantified the resource requirements for delirium detection approaches. | Evidence for time to perform the delirium diagnosis for each strategy is clear with high certainty.  Evidence of time to train for the test, or the monetary cost of the implementation, is largely unknown, with indirect evidence from other training and screening tests. |
| **Cost effectiveness** Does the cost-effectiveness of the intervention favor the intervention or the comparison? | | |
| **JUDGEMENT** | **RESEARCH EVIDENCE** | **ADDITIONAL CONSIDERATIONS** |
| ○ Favors the comparison ○ Probably favors the comparison ○ Does not favor either the intervention or the comparison ○ Probably favors the intervention ○ Favors the intervention ○ Varies X No included studies | No direct or indirect evidence focused on cost effectiveness of delirium detection strategies in the ED was identified. | Comparing “Nursing Delirium Screening Scale (Nu-DESC) vs Confusion Rating Scale (CRS) in hospitalized oncology patients: “Virtually no increased cost in time, effort or money has been associated with Nu-DESC delirium screening, since it can be completed as fast as the CRS and is a part of routine clinical care performed by regular nursing personnel. As presented above, there appears to be an improvement in detection of delirium associated with the use of the Nu-DESC. Cost-effectiveness of using the Nu-DESC thus appears to be high.[^66^](https://paperpile.com/c/aGrzfz/GEhan)  Testing the 4AT in hospitalized patients: “The estimated cost of false-positive cases was £4653, of false-negative cases was £8956, and of a missed diagnosis was £2067.”[^67^](https://paperpile.com/c/aGrzfz/k8gmt) |
| **Equity** What would be the impact on health equity? | | |
| **JUDGEMENT** | **RESEARCH EVIDENCE** | **ADDITIONAL CONSIDERATIONS** |
| ○ Reduced ○ Probably reduced ○ Probably no impact ○ Probably increased ○ Increased ○ Varies X Don't know | No direct or indirect evidence explored health inequities associated with different delirium detection approaches. Sociodemographic characteristics, specifically race and ethnicity, are inconsistently reported. Studies frequently exclude older adults with cognitive impairment and non-English primary languages, and strategies are needed to include them in EM research.[^68^](https://paperpile.com/c/aGrzfz/Ud0Ev) | Qualitative research of ED physicians identifies language barriers as a common barrier to the recognition of delirium.[^6^](https://paperpile.com/c/aGrzfz/DulHv)    **Language**:  **Non-English Speaking**  Qualitative study of nurses’ perspectives on use of CAM with patients with limited English proficiency, which showed that most research to date excluded patients who did not speak primary language of the country, therefore unanswered questions persist around the comparative delirium risk approach discrimination and calibration when there is language discordance between examiner and patient.[^69,70^](https://paperpile.com/c/aGrzfz/LsyAe+JKQDE)  In a systematic review of 31 international investigations examining delirium screening tools in hospitalized individuals, all but four studies excluded non-native language speakers. These screening tools included CAM, 4AT, and NuDESC.[^71^](https://paperpile.com/c/aGrzfz/owrbK)  All the studied screening tools utilize a linguistic component to varying levels, and therefore may perform differently in patients (or caregivers) who Prefer Language Other Than English.  The only exception is RASS, which does not utilize any linguistic component and therefore is not expected to perform differently.  The discordance in the language between the clinical staff and the patient/caregiver is thought to be the cause of the barrier, as some of these instruments have been translated to languages other than English.  Patients with delirium and a non-English preferred language had an increased risk of receiving a physical restraint (RR 2.61, 95% CI 1.40-4.85) compared to patients who preferred English.[^72^](https://paperpile.com/c/aGrzfz/jFbVX) They also had an increased risk of receiving orders for new antipsychotic medications (RR 1.50, 95% CI 1.03–2.19).[^72^](https://paperpile.com/c/aGrzfz/jFbVX)  **Socioeconomic status**  Given the resources required to train the staff (whether nurses or physicians) on performing these tests and implementing a documentation system, it is possible that affluent hospitals are more likely to adopt a highly-sensitive formal screening process, whereas less affluent hospitals are more likely to adopt an informal screening process such as clinical gestalt, which is less sensitive. This may lead to a downstream effect in a difference in detecting delirium in patients of different socioeconomic backgrounds.  Area deprivation index was associated with delirium incidence and severity of delirium: “Neighborhood-level makers of social disadvantage are associated with delirium incidence and severity, and demonstrated an exposure-response relationship.”[^73^](https://paperpile.com/c/aGrzfz/Fjjp4) |
| **Acceptability** Is the intervention acceptable to key stakeholders? | | |
| **JUDGEMENT** | **RESEARCH EVIDENCE** | **ADDITIONAL CONSIDERATIONS** |
| ○ No X Probably no ○ Probably yes ○ Yes ○ Varies ○ Don't know | Emergency medicine physicians note several reservations regarding ED delirium screening, including difficulties implementing non-pharmacologic delirium prevention/treatment protocols, clinical time constraints, inadequate knowledge about non-pharmacologic approaches, and under-appreciation of the significance of unrecognized delirium.[^43^](https://paperpile.com/c/aGrzfz/uH3DR) | The lack of a definitive delirium prevention or delirium management intervention raises questions about the ethics, malpractice risk, and utility of delirium delirium detection.[^1^](https://paperpile.com/c/aGrzfz/jJ5Gw)  ICU patients who experienced delirium report delirium as a distressing experience, including strange and scary hallucinations and/or delusions, which provides impetus for ongoing assessments of the detection and management of delirium.[^74^](https://paperpile.com/c/aGrzfz/a4lv4)  In a qualitative study of inpatient nursing perspectives on screening, delirium screening perceived to be a low value activity by some nurses as positive screens do not trigger any alerts or interventions or lead to a change in clinical management.[^75^](https://paperpile.com/c/aGrzfz/2nzqX)  Patients may not tolerate repeated demands to perform such tests.[^76^](https://paperpile.com/c/aGrzfz/NL6cb)  Some patients express frustration when delirium protocol questions are perceived as irrelevant to their medical condition.[^77^](https://paperpile.com/c/aGrzfz/BZYBl)  In hospitalized patients, UB-CAM screening (which screens for delirium) was accepted.[^77^](https://paperpile.com/c/aGrzfz/BZYBl) |
| **Feasibility** Is the intervention feasible to implement? | | |
| **JUDGEMENT** | **RESEARCH EVIDENCE** | **ADDITIONAL CONSIDERATIONS** |
| ○ No ○ Probably no ○ Probably yes ○ Yes X Varies ○ Don't know | The majority of ACEP GEDA Level 1 sites and nearly half of Level 2 sites report delirium detection protocols.[^3^](https://paperpile.com/c/aGrzfz/ZUKiA) However, the majority of EDs in the United States and worldwide are not GEDA certified nor planning on becoming GEDA certified, nor have delirium detection strategies been assessed in non-academic real-world settings. Furthermore, the fidelity with which GEDA sites are using delirium detection approaches is unknown. | One Swiss hospital created a hospital-wide delirium management guideline which included delirium detection.[^78^](https://paperpile.com/c/aGrzfz/dEgUn)  Irish national guidance recommends daily delirium screening for all acute hospital inpatients at risk of delirium. “Recognizing Acute Delirium As part of your Routine” was considered the approach to detecting delirium with the best balance between implementation feasibility and diagnostic accuracy in a series of stakeholder workshops.[^79^](https://paperpile.com/c/aGrzfz/x9CIU) |

# **SUMMARY OF JUDGEMENTS**

|  | **JUDGEMENT** | | | | | | |
| --- | --- | --- | --- | --- | --- | --- | --- |
| **PROBLEM** | No | Probably no | Probably yes | **Yes** |  | Varies | Don't know |
| **TEST ACCURACY** | Very inaccurate | Inaccurate | Accurate | Very accurate |  | **Varies** | Don't know |
| **DESIRABLE EFFECTS** | **Trivial** | Small | Moderate | Large |  | Varies | Don't know |
| **UNDESIRABLE EFFECTS** | Large | Moderate | Small | Trivial |  | Varies | **Don't know** |
| **CERTAINTY OF THE EVIDENCE OF TEST ACCURACY** | **Very low** | Low | Moderate | High |  |  | No included studies |
| **CERTAINTY OF THE EVIDENCE OF TEST'S EFFECTS** | Very low | **Low** | Moderate | High |  |  | No included studies |
| **CERTAINTY OF THE EVIDENCE OF MANAGEMENT'S EFFECTS** | **Very low** | Low | Moderate | High |  |  | No included studies |
| **CERTAINTY OF THE EVIDENCE OF TEST RESULT/MANAGEMENT** | **Very low** | Low | Moderate | **High** |  |  | No included studies |
| **CERTAINTY OF EFFECTS** | **Very low** | Low | Moderate | **High** |  |  | No included studies |
| **VALUES** | Important uncertainty or variability | **Possibly important uncertainty or variability** | Probably no important uncertainty or variability | No important uncertainty or variability |  |  |  |
| **BALANCE OF EFFECTS** | Favors the comparison | Probably favors the comparison | Does not favor either the intervention or the comparison | Probably favors the intervention | Favors the intervention | Varies | **Don't know** |
| **RESOURCES REQUIRED** | Large costs | Moderate costs | Negligible costs and savings | Moderate savings | Large savings | **Varies** | Don't know |
| **CERTAINTY OF EVIDENCE OF REQUIRED RESOURCES** | Very low | **Low** | Moderate | High |  |  | No included studies |
| **COST EFFECTIVENESS** | Favors the comparison | Probably favors the comparison | Does not favor either the intervention or the comparison | Probably favors the intervention | Favors the intervention | Varies | **No included studies** |
| **EQUITY** | Reduced | Probably reduced | Probably no impact | Probably increased | Increased | Varies | **Don't know** |
| **ACCEPTABILITY** | No | **Probably no** | Probably yes | Yes |  | Varies | Don't know |
| **FEASIBILITY** | No | Probably no | Probably yes | Yes |  | **Varies** | Don't know |

# **TYPE OF RECOMMENDATION**

| Strong recommendation against the intervention | Conditional recommendation against the intervention | Conditional recommendation for either the intervention or the comparison | **Conditional recommendation for the intervention** | Strong recommendation for the intervention |
| --- | --- | --- | --- | --- |
| ○ | ○ | ○ | X | ○ |

# **CONCLUSIONS**

| **Recommendation** |
| --- |
| Recommendation: Use ED physician-administered 4AT, bCAM, CAM-ICU, mCAM, AMT-4, or RASS to rule-in or rule-out delirium. (Conditional, FOR) [Very Low certainty of evidence]  Recommendation: Use ED physician-administered DTS to rule-out but not to rule-in delirium. (Conditional, FOR) [Very Low certainty of evidence] |
|  |
| **Justification** |
| Diagnostic accuracy studies have been conducted in academic centers (limited external validity) and have not evaluated the potential benefits or harms associated with ED delirium detection (diagnostic impact). The majority of studies did not use the same gold standard and the QUADAS-2 indicates multiple potential sources of bias. |

| **Subgroup considerations** |
| --- |
| Some studies reported on the subgroup of patients with dementia.  No subgroup accuracy reported for hyperactive delirium, hypoactive delirium, incident delirium, or prevalent (Pre-ED) delirium. |
| **Implementation considerations** |
| Implementation approaches for ED delirium detection will need to contemplate local cultural capacity for change, applicable behavioral change models, resource requirements, explanatory versus pragmatic design models, fidelity, adaptability, and sustainability.[^40,80,81^](https://paperpile.com/c/aGrzfz/jTZVI+eAoLK+EmxE8) |

| **Monitoring and evaluation** |
| --- |
| Process quality improvement efforts, including numerator and denominator of patients and thresholds for minimally acceptable delirium detection exist.[^21^](https://paperpile.com/c/aGrzfz/xRp5Y) In addition to local delirium screening and identification rates, the potential harms of efforts to improve delirium detection including care delays, anchoring bias, or premature closure should be monitored. |
| **Research priorities** |
| The following research priorities exist for ED delirium screening accuracy.  a) Quantifying the accuracy of various delirium identification strategies for etiologic phenotypes (medication vs. infectious disease vs. delirium overlying dementia vs. other) and delirium motor subtypes would provide depth of understanding for ideal use cases for individual dementia detection approaches.[^10^](https://paperpile.com/c/aGrzfz/AqCnh)  b) Cost analyses and cost-effectiveness research are lacking from any perspective with anticipated costs to include nurse/physician training requirements and potential savings to include avoidable healthcare costs.  c) Implementation Science research exploring acceptability and feasibility of ED delirium detection strategies in addition to fidelity, adaptability, and sustainability of protocols to identify the presence or absence of delirium.[^82,83^](https://paperpile.com/c/aGrzfz/tzbyF+R60dB) |

**References**

1. [Lee S, Chen H, Hibino S, et al. Can we improve delirium prevention and treatment in the emergency department? A systematic review. *J Am Geriatr Soc*. 2022;70(6):1838-1849.](http://paperpile.com/b/aGrzfz/jJ5Gw)

2. [American College of Emergency Physicians, American Geriatrics Society, Emergency Nurses Association, Society for Academic Emergency Medicine, Geriatric Emergency Department Guidelines Task Force. Geriatric emergency department guidelines. *Ann Emerg Med*. 2014;63(5):e7-e25.](http://paperpile.com/b/aGrzfz/3Olhh)

3. [Kennedy M, Lesser A, Israni J, et al. Reach and adoption of a Geriatric Emergency Department Accreditation program in the United States. *Ann Emerg Med*. 2022;79(4):367-373.](http://paperpile.com/b/aGrzfz/ZUKiA)

4. [Schnitker LM, Martin-Khan M, Burkett E, et al. Structural quality indicators to support quality of care for older people with cognitive impairment in emergency departments. *Acad Emerg Med*. 2015;22(3):273-284.](http://paperpile.com/b/aGrzfz/f5qH1)

5. [Schnitker LM, Martin-Khan M, Burkett E, et al. Process quality indicators targeting cognitive impairment to support quality of care for older people with cognitive impairment in emergency departments. *Acad Emerg Med*. 2015;22(3):285-298.](http://paperpile.com/b/aGrzfz/BYxZm)

6. [Chary AN, Castilla-Ojo N, Joshi C, et al. Evaluating older adults with cognitive dysfunction: A qualitative study with emergency clinicians. *J Am Geriatr Soc*. 2022;70(2):341-351.](http://paperpile.com/b/aGrzfz/DulHv)

7. [Shih RD, Carpenter CR, Tolia V, Binder EF, Ouslander JG. Balancing vision with pragmatism: The geriatric emergency department guidelines-realistic expectations from emergency medicine and geriatric medicine. *J Am Geriatr Soc*. 2022;70(5):1368-1373.](http://paperpile.com/b/aGrzfz/SVlpC)

8. [Assa AH, Wicks MN, Umberger RA. Family caregivers’ experience of patients with delirium in critical care units: A state-of-the-science integrative review. *Am J Crit Care*. 2021;30(6):471-478.](http://paperpile.com/b/aGrzfz/GEvpq)

9. [Mahmoud A, Raghuraman S, Richards E, et al. 2218 Experience of family caregivers for older patients with delirium: a qualitative study. *Age Ageing*. 2024;53(Supplement_3). doi:](http://paperpile.com/b/aGrzfz/7GnQQ)[10.1093/ageing/afae139.079](http://dx.doi.org/10.1093/ageing/afae139.079)

10. [Carpenter CR, Hammouda N, Linton EA, et al. Delirium prevention, detection, and treatment in emergency medicine settings: A Geriatric Emergency care Applied Research (GEAR) Network scoping review and consensus statement. *Acad Emerg Med*. 2021;28(1):19-35.](http://paperpile.com/b/aGrzfz/AqCnh)

11. [Hustey FM, Meldon SW. The prevalence and documentation of impaired mental status in elderly emergency department patients. *Ann Emerg Med*. 2002;39(3):248-253.](http://paperpile.com/b/aGrzfz/55uXg)

12. [Han JH, Zimmerman EE, Cutler N, et al. Delirium in older emergency department patients: recognition, risk factors, and psychomotor subtypes. *Acad Emerg Med*. 2009;16(3):193-200.](http://paperpile.com/b/aGrzfz/fweGj)

13. [Suffoletto B, Miller T, Frisch A, Callaway C. Emergency physician recognition of delirium. *Postgrad Med J*. 2013;89(1057):621-625.](http://paperpile.com/b/aGrzfz/shvWi)

14. [Kakuma R, du Fort GG, Arsenault L, et al. Delirium in older emergency department patients discharged home: effect on survival. *J Am Geriatr Soc*. 2003;51(4):443-450.](http://paperpile.com/b/aGrzfz/cy2C5)

15. [McCusker J, Cole MG, Dendukuri N, Belzile E. Does delirium increase hospital stay? *J Am Geriatr Soc*. 2003;51(11):1539-1546.](http://paperpile.com/b/aGrzfz/Z4PCV)

16. [Fong TG, Jones RN, Shi P, et al. Delirium accelerates cognitive decline in Alzheimer disease. *Neurology*. 2009;72(18):1570-1575.](http://paperpile.com/b/aGrzfz/pzXFN)

17. [Langan C, Sarode DP, Russ TC, Shenkin SD, Carson A, Maclullich AM. Psychiatric symptomatology after delirium: a systematic review Psychogeriatrics. 2017;17:327-337.](http://paperpile.com/b/aGrzfz/2iSlc)

18. [Han JH, Shintani A, Eden S, et al. Delirium in the emergency department: an independent predictor of death within 6 months. *Ann Emerg Med*. 2010;56(3):244-252.e1.](http://paperpile.com/b/aGrzfz/0AfrB)

19. [Kennedy M, Enander RA, Tadiri SP, Wolfe RE, Shapiro NI, Marcantonio ER. Delirium risk prediction, healthcare use and mortality of elderly adults in the emergency department. *J Am Geriatr Soc*. 2014;62(3):462-469.](http://paperpile.com/b/aGrzfz/DpgeV)

20. [Han JH, Schnelle JF, Ely EW. The relationship between a chief complaint of “altered mental status” and delirium in older emergency department patients. *Acad Emerg Med*. 2014;21(8):937-940.](http://paperpile.com/b/aGrzfz/DwcMv)

21. [Carpenter CR, Lee S, Kennedy M, et al. Delirium detection in the emergency department: A diagnostic accuracy meta-analysis of history, physical examination, laboratory tests, and screening instruments. *Acad Emerg Med*. 2024;31(10):1014-1036.](http://paperpile.com/b/aGrzfz/xRp5Y)

22. [Gagné AJ, Voyer P, Boucher V, et al. Performance of the French version of the 4AT for screening the elderly for delirium in the emergency department. *CJEM*. 2018;20(6):903-910.](http://paperpile.com/b/aGrzfz/yqACa)

23. [O’Sullivan D, Brady N, Manning E, et al. Validation of the 6-Item Cognitive Impairment Test and the 4AT test for combined delirium and dementia screening in older Emergency Department attendees. *Age Ageing*. 2018;47(1):61-68.](http://paperpile.com/b/aGrzfz/BWkuo)

24. [Shenkin SD, Fox C, Godfrey M, et al. Delirium detection in older acute medical inpatients: a multicentre prospective comparative diagnostic test accuracy study of the 4AT and the confusion assessment method. *BMC Med*. 2019;17(1):138.](http://paperpile.com/b/aGrzfz/uqH0t)

25. [Arendts G, Love J, Nagree Y, Bruce D, Hare M, Dey I. Rates of delirium diagnosis do not improve with emergency risk screening: Results of the emergency department delirium initiative trial. *J Am Geriatr Soc*. 2017;65(8):1810-1815.](http://paperpile.com/b/aGrzfz/pEZFm)

26. [Dyer AH, Briggs R, Nabeel S, O’Neill D, Kennelly SP. The Abbreviated Mental Test 4 for cognitive screening of older adults presenting to the Emergency Department. *Eur J Emerg Med*. 2017;24(6):417-422.](http://paperpile.com/b/aGrzfz/1iOhL)

27. [Mailhot T, Darling C, Ela J, Malyuta Y, Inouye SK, Sacyznski J. Family identification of delirium in the emergency department in patients with and without Dementia: validity of thefamily confusion assessment method (FAM-CAM). *J Am Geriatr Soc*. 2020;68:983-990.](http://paperpile.com/b/aGrzfz/zUAyN)

28. [Han JH, Vasilevskis EE, Schnelle JF, et al. The diagnostic performance of the Richmond agitation sedation scale for detecting delirium in older emergency department patients. *Acad Emerg Med*. 2015;22(7):878-882.](http://paperpile.com/b/aGrzfz/Ep0ID)

29. [Han JH, Wilson A, Graves AJ, et al. Validation of the Confusion Assessment Method for the Intensive Care Unit in older emergency department patients. *Acad Emerg Med*. 2014;21(2):180-187.](http://paperpile.com/b/aGrzfz/lxMjR)

30. [Grossmann FF, Hasemann W, Kressig RW, Bingisser R, Nickel CH. Performance of the modified Richmond Agitation Sedation Scale in identifying delirium in older ED patients. *Am J Emerg Med*. 2017;35(9):1324-1326.](http://paperpile.com/b/aGrzfz/ORy9o)

31. [Byrt T. How good is that agreement? *Epidemiology*. 1996;7(5):561.](http://paperpile.com/b/aGrzfz/WNFq9)

32. [Gallo L, Hua N, Mercuri M, Silveira A, Worster A, Best Evidence in Emergency Medicine (BEEM; beem.ca). Adherence to standards for reporting Diagnostic Accuracy in emergency medicine research. *Acad Emerg Med*. 2017;24(8):914-919.](http://paperpile.com/b/aGrzfz/rkc3Z)

33. [Hayden SR, Brown MD. Likelihood ratio: A powerful tool for incorporating the results of a diagnostic test into clinical decisionmaking. *Ann Emerg Med*. 1999;33(5):575-580.](http://paperpile.com/b/aGrzfz/qc2RW)

34. [Han JH, Wilson A, Vasilevskis EE, et al. Diagnosing delirium in older emergency department patients: validity and reliability of the delirium triage screen and the brief confusion assessment method. *Ann Emerg Med*. 2013;62(5):457-465.](http://paperpile.com/b/aGrzfz/cyW8P)

35. [Vonnes C, Tofthagen C. Impacting outcomes in the hospitalized oncology patient: Evidence-informed quality and safety project to implement routine screening for delirium. *PatientSaf*. Published online September 16, 2022:20-29.](http://paperpile.com/b/aGrzfz/f5fbt)

36. [Brummel NE, Vasilevskis EE, Han JH, Boehm L, Pun BT, Ely EW. Implementing delirium screening in the ICU: secrets to success. *Crit Care Med*. 2013;41(9):2196-2208.](http://paperpile.com/b/aGrzfz/aDUyX)

37. [Graf S, Hediger H, Knüppel Lauener S. Delirium at the Hospital - Nursing effort and risk of falling: A Routine data analysis: Eine Routinedatenanalyse. *Pflege*. 2020;33(3):133-142.](http://paperpile.com/b/aGrzfz/FImLQ)

38. [Ista E, Trogrlic Z, Bakker J, Osse RJ, van Achterberg T, van der Jagt M. Improvement of care for ICU patients with delirium by early screening and treatment: study protocol of iDECePTIvE study. *Implement Sci*. 2014;9(1):143.](http://paperpile.com/b/aGrzfz/oIfOd)

39. [Southerland LT, Hunold KM, Van Fossen J, et al. An implementation science approach to geriatric screening in an emergency department. *J Am Geriatr Soc*. 2022;70(1):178-187.](http://paperpile.com/b/aGrzfz/mG8zm)

40. [Southerland LT, Gulker P, Van Fossen J, et al. Implementation of geriatric screening in the emergency department using the Consolidated Framework for Implementation Research. *Acad Emerg Med*. 2023;30(11):1117-1128.](http://paperpile.com/b/aGrzfz/eAoLK)

41. [Oldham MA, Heinrich T, Luccarelli J. Requesting that delirium achieve parity with acute encephalopathy in the MS-DRG system. *J Acad Consult Liaison Psychiatry*. 2024;65(3):302-312.](http://paperpile.com/b/aGrzfz/uhnsi)

42. [Davenport K, Cameron A, Samson M, Sri-On J, Liu SW. Fall prevention knowledge, attitudes, and behaviors: A survey of emergency providers. *West J Emerg Med*. 2020;21(4):826-830.](http://paperpile.com/b/aGrzfz/FdV6K)

43. [Chary AN, Lesser A, Inouye SK, Carpenter CR, Stuck AR, Kennedy M. A survey of delirium self-reported knowledge and practices among emergency physicians in the United States. *J Geriatr Emerg Med*. 2021;2(12). doi:](http://paperpile.com/b/aGrzfz/uH3DR)[10.17294/2694-4715.1010](http://dx.doi.org/10.17294/2694-4715.1010)

44. [Duggan MC, Van J, Ely EW. Delirium assessment in critically ill older adults: Considerations during the COVID-19 pandemic. *Crit Care Clin*. 2021;37(1):175-190.](http://paperpile.com/b/aGrzfz/bqlnn)

45. [Hustey FM, Meldon SW, Smith MD, Lex CK. The effect of mental status screening on the care of elderly emergency department patients. *Ann Emerg Med*. 2003;41(5):678-684.](http://paperpile.com/b/aGrzfz/ROsyo)

46. [Reeves RR, Parker JD, Burke RS, Hart RH. Inappropriate psychiatric admission of elderly patients with unrecognized delirium. *South Med J*. 2010;103(2):111-115.](http://paperpile.com/b/aGrzfz/NslMb)

47. [Han JH, Bryce SN, Ely EW, et al. The effect of cognitive impairment on the accuracy of the presenting complaint and discharge instruction comprehension in older emergency department patients. *Ann Emerg Med*. 2011;57(6):662-671.e2.](http://paperpile.com/b/aGrzfz/pOr2V)

48. [Tong T, Chignell M, Tierney MC, Lee J. A serious game for clinical assessment of cognitive status: Validation study. *JMIR Serious Games*. 2016;4(1):e7.](http://paperpile.com/b/aGrzfz/tHbpl)

49. [Mailhot T, Darling C, Ela J, Malyuta Y, Inouye SK, Saczynski J. Family identification of delirium in the emergency department in patients with and without dementia: Validity of the Family Confusion Assessment Method (FAM-CAM). *J Am Geriatr Soc*. 2020;68(5):983-990.](http://paperpile.com/b/aGrzfz/YNmeR)

50. [Burry L, Mehta S, Perreault MM, et al. Antipsychotics for treatment of delirium in hospitalised non-ICU patients. *Cochrane Database Syst Rev*. 2018;6(6):CD005594.](http://paperpile.com/b/aGrzfz/Hc58E)

51. [Candy B, Jackson KC, Jones L, Leurent B, Tookman A, King M. Drug therapy for delirium in terminally ill adult patients. *Cochrane Database Syst Rev*. 2012;11:CD004770.](http://paperpile.com/b/aGrzfz/ZNHV0)

52. [Yu A, Wu S, Zhang Z, et al. Cholinesterase inhibitors for the treatment of delirium in non-ICU settings. *Cochrane Database Syst Rev*. 2018;6:CD012494.](http://paperpile.com/b/aGrzfz/nrwsD)

53. [Woodhouse R, Burton JK, Rana N, Pang YL, Lister JE, Siddiqi N. Interventions for preventing delirium in older people in institutional long-term care. *Cochrane Database Syst Rev*. 2019;4:CD009537.](http://paperpile.com/b/aGrzfz/rvCpk)

54. [Herling SF, Greve IE, Vasilevskis EE, et al. Interventions for preventing intensive care unit delirium in adults. *Cochrane Database Syst Rev*. 2018;11(1):CD009783.](http://paperpile.com/b/aGrzfz/c7Cla)

55. [Burton JK, Craig LE, Yong SQ, et al. Non-pharmacological interventions for preventing delirium in hospitalised non-ICU patients. *Cochrane Database Syst Rev*. 2021;7(7):CD013307.](http://paperpile.com/b/aGrzfz/MYOgQ)

56. [Cohen RG. Ageism in health care? Yep, it’s a thing. *The Boston globe*. April 28, 2022.](http://paperpile.com/b/aGrzfz/gAjnZ)

57. [Chera T, Tinetti M, Travers J, et al. “What Matters” in the emergency department: A prospective analysis of older adults’ concerns and desired outcomes. *Med Care*. 2024;62(12 Suppl 1):S50-S56.](http://paperpile.com/b/aGrzfz/g6QSd)

58. [Eagles D, Cheung WJ, Avlijas T, et al. Barriers and facilitators to nursing delirium screening in older emergency patients: a qualitative study using the theoretical domains framework. *Age Ageing*. 2022;51(1). doi:](http://paperpile.com/b/aGrzfz/wsq0V)[10.1093/ageing/afab256](http://dx.doi.org/10.1093/ageing/afab256)

59. [Lee JS, Tong T, Chignell M, et al. Prevalence, management and outcomes of unrecognized delirium in a National Sample of 1,493 older emergency department patients: how many were sent home and what happened to them? *Age Ageing*. 2022;51(2). doi:](http://paperpile.com/b/aGrzfz/f5tgi)[10.1093/ageing/afab214](http://dx.doi.org/10.1093/ageing/afab214)

60. [Nowroozpoor A, Dussetschleger J, Perry W, et al. Detecting cognitive impairment and dementia in the emergency department: A scoping review. *J Am Med Dir Assoc*. 2022;23(8):1314.e31-e1314.e88.](http://paperpile.com/b/aGrzfz/imt9z)

61. [Han JH, Wilson A, Graves AJ, Shintani A, Schnelle JF, Ely EW. A quick and easy delirium assessment for nonphysician research personnel. *Am J Emerg Med*. 2016;34(6):1031-1036.](http://paperpile.com/b/aGrzfz/VU6zd)

62. [Inouye SK. *The Confusion Assessment Method (CAM): Training Manual and Coding Guide*. Hospital Elder Life Program; 2003.](http://paperpile.com/b/aGrzfz/YGwdU)

63. [Hasemann W, Grossmann FF, Stadler R, et al. Screening and detection of delirium in older ED patients: performance of the modified Confusion Assessment Method for the Emergency Department (mCAM-ED). A two-step tool. *Intern Emerg Med*. Published online December 30, 2017. doi:](http://paperpile.com/b/aGrzfz/ZIeVu)[10.1007/s11739-017-1781-y](http://dx.doi.org/10.1007/s11739-017-1781-y)

64. [Hargrave A, Bastiaens J, Bourgeois JA, et al. Validation of a nurse-based delirium-screening tool for hospitalized patients. *Psychosomatics*. 2017;58(6):594-603.](http://paperpile.com/b/aGrzfz/6alBg)

65. [Migdal VL, Harper K, Haqqani N, Janiak B. Time cost of standardized nursing screens in the emergency department. *West J Emerg Med*. 2019;20(6):851-854.](http://paperpile.com/b/aGrzfz/AadZ3)

66. [Gaudreau JD, Gagnon P, Harel F, Roy MA. Impact on delirium detection of using a sensitive instrument integrated into clinical practice. *Gen Hosp Psychiatry*. 2005;27(3):194-199.](http://paperpile.com/b/aGrzfz/GEhan)

67. [MacLullich AM, Shenkin SD, Goodacre S, et al. The 4 “A”s test for detecting delirium in acute medical patients: a diagnostic accuracy study. *Health Technol Assess*. 2019;23(40):1-194.](http://paperpile.com/b/aGrzfz/k8gmt)

68. [Chary AN, Suh M, Ordoñez E, et al. A scoping review of geriatric emergency medicine research transparency in diversity, equity, and inclusion reporting. *J Am Geriatr Soc*. 2024;72(11):3551-3566.](http://paperpile.com/b/aGrzfz/Ud0Ev)

69. [Reppas-Rindlisbacher C, Panov ED, Cuperfain AB, Rawal S. A survey of nurses’ perspectives on delirium screening in older adult medical inpatients with limited English proficiency. *J Gerontol Nurs*. 2021;47(4):29-34.](http://paperpile.com/b/aGrzfz/LsyAe)

70. [Chary AN, Torres B, Brickhouse E, et al. Language discordance in emergency department delirium screening: Results from a qualitative interview-based study. *J Am Geriatr Soc*. 2023;71(4):1328-1331.](http://paperpile.com/b/aGrzfz/JKQDE)

71. [De J, Wand APF. Delirium screening: A systematic review of delirium screening tools in hospitalized patients. *Gerontologist*. 2015;55(6):1079-1099.](http://paperpile.com/b/aGrzfz/owrbK)

72. [Reppas-Rindlisbacher C, Shin S, Purohit U, et al. Association between non-English language and use of physical and chemical restraints among medical inpatients with delirium. *J Am Geriatr Soc*. 2022;70(12):3640-3643.](http://paperpile.com/b/aGrzfz/jFbVX)

73. [Arias F, Chen F, Fong TG, et al. Neighborhood-level social disadvantage and risk of delirium following major surgery. *J Am Geriatr Soc*. 2020;68(12):2863-2871.](http://paperpile.com/b/aGrzfz/Fjjp4)

74. [la Cour KN, Andersen-Ranberg NC, Mortensen C, et al. Patient recall of intensive care delirium: A qualitative investigation. *Acta Anaesthesiol Scand*. 2024;68(8):1050-1058.](http://paperpile.com/b/aGrzfz/a4lv4)

75. [Ragheb J, Norcott A, Benn L, et al. Barriers to delirium screening and management during hospital admission: a qualitative analysis of inpatient nursing perspectives. *BMC Health Serv Res*. 2023;23(1):712.](http://paperpile.com/b/aGrzfz/2nzqX)

76. [Pendlebury ST. Delirium screening in older patients. *Age Ageing*. 2018;47(5):635-637.](http://paperpile.com/b/aGrzfz/NL6cb)

77. [Husser EK, Fick DM, Boltz M, et al. Implementing a rapid, two-step delirium screening protocol in acute care: Barriers and facilitators. *J Am Geriatr Soc*. 2021;69(5):1349-1356.](http://paperpile.com/b/aGrzfz/BZYBl)

78. [Schubert M, Schürch R, Boettger S, et al. A hospital-wide evaluation of delirium prevalence and outcomes in acute care patients - a cohort study. *BMC Health Serv Res*. 2018;18(1):550.](http://paperpile.com/b/aGrzfz/dEgUn)

79. [O’Donnell N, Doyle M, Laffan S, et al. 184 Paying attention to the feasibility of daily delirium screening in an acute hospital setting. *Age Ageing*. 2023;52(Supplement_3). doi:](http://paperpile.com/b/aGrzfz/x9CIU)[10.1093/ageing/afad156.161](http://dx.doi.org/10.1093/ageing/afad156.161)

80. [Carpenter CR, Southerland LT, Lucey BP, Prusaczyk B. Around the EQUATOR with clinician-scientists transdisciplinary aging research (Clin-STAR) principles: Implementation science challenges and opportunities. *J Am Geriatr Soc*. 2022;70(12):3620-3630.](http://paperpile.com/b/aGrzfz/jTZVI)

81. [Chary AN, Bhananker AR, Brickhouse E, et al. Implementation of delirium screening in the emergency department: A qualitative study with early adopters. *J Am Geriatr Soc*. 2024;72(12):3753-3762.](http://paperpile.com/b/aGrzfz/EmxE8)

82. [Pinnock H, Barwick M, Carpenter CR, et al. Standards for reporting implementation studies (StaRI) statement. *BMJ*. Published online March 6, 2017:i6795.](http://paperpile.com/b/aGrzfz/tzbyF)

83. [Carpenter CR, Southerland LT, Lucey BP, Prusaczyk B. Around the EQUATOR with clinician-scientists transdisciplinary aging research (Clin-STAR) principles: implementation science challenges and opportunities J Am Geriatr Soc 2022. *J Am Geriatr Soc*. Published online 2022.](http://paperpile.com/b/aGrzfz/R60dB)

Appendix S3. EtD document PICO3

| **Question** | |
| --- | --- |
| **Should older adults with delirium in the emergency department get a non-contrast head CT or not?** | |
| **POPULATION:** | Older adults (65 years and older) presenting to the emergency department with delirium |
| INTERVENTION (Exposure): | Head computed tomography (CT) |
| **PURPOSE OF THE TEST:** | Identifying subset of delirium patients who would have clinical benefit from identifying delirium-causative pathology identified by head CT. |
| **ROLE OF THE TEST:** | Rule in or rule out acute abnormal findings on head CT as a cause of delirium |
| **LINKED TREATMENTS:** | Specific treatment dependent on the CT finding:  **Stroke**: reperfusion therapy if ischemic (24.3% (43/202) of ischemic stroke had acute confusion according to Henon [^1–3^](https://paperpile.com/c/HSTcT3/DzXKr+wARye+4x3Gv)), neurosurgery if hemorrhagic, acute blood pressure management, secondary prevention (e.g. antiplatelet therapy, risk factor modification), anticoagulant reversal (if applicable)  **Intracranial hemorrhage**: neurosurgery, acute blood pressure management, anticoagulant reversal (if applicable)[^4^](https://paperpile.com/c/HSTcT3/e99XN)  **Tumor**: surgery, steroids, radiation[^5^](https://paperpile.com/c/HSTcT3/ziU3U) |
| **ANTICIPATED OUTCOMES:** | Accurately and expeditiously diagnosing acute clinically significant intracranial abnormality to improve clinical recovery |
| **SETTING:** | ED |
| **PERSPECTIVE:** | Patient, care partner, nurse, physician, healthcare system |
| **BACKGROUND:** | Older adults presenting to the ED with delirium are more likely than those without delirium to have a head CT as part of their workup.[^6^](https://paperpile.com/c/HSTcT3/wGFEf) However, the yield of this test is unclear. Furthermore, obtaining a head CT in an (Background cont) acutely confused patient may be logistically challenging, necessitating sedation and other resources, with an unclear clinical benefit.  MRI would be a more accurate imaging modality to identify acute abnormalities as a cause of the delirium than CT. However, its lack of availability and expense make it quite impractical in the ED setting, therefore we focused our discussion on head CT only.  The association between the emergency department (ED) diagnosis of delirium in older adults and abnormal CT head imaging is not well delineated.[^7^](https://paperpile.com/c/HSTcT3/DIHj4) However, since most hospitals do not screen for delirium,[^8^](https://paperpile.com/c/HSTcT3/KfuK0) that delirium is heterogeneous and there is no gold standard definition, screening tool or uniformly accepted detection approach, this is not surprising. Therefore, this evidence synthesis is based entirely on indirect evidence exploring the diagnostic utility of CT for altered mental status (AMS) or acute confusional state.  In general, infection is the most common identified etiology among older ED patients with AMS (39.5%) and neurologic diseases ( ischemic stroke, intraparenchymal hemorrhage, status epilepticus/prolonged postictal, intracranial mass/shift, chronic subdural/SAH, nonconvulsive status, Parkinson/Alzheimer exacerbations)  account for 36.5%.[^9^](https://paperpile.com/c/HSTcT3/fNWBf)    Many clinical practitioners do not recognize (or document) delirium as a separate disease entity from acute AMS. [^10–12^](https://paperpile.com/c/HSTcT3/0JV3p+htW5y+BJo18) It is unclear whether the diagnosis of altered mental status relates to the diagnosis of delirium. One study found that AMS was highly specific for delirium (98.9% specific (95% CI 97.2% to 99.6%)).[^13^](https://paperpile.com/c/HSTcT3/t73KO) In this study, AMS was only 38.0% sensitive (95% CI 25.9% to 51.9%) for delirium implying that substituting AMS for delirium does identify cases of delirium, but missed over half of all delirium cases. However Aslaner et al.[^9^](https://paperpile.com/c/HSTcT3/fNWBf) found that only 56% of patients with AMS had delirium (much lower specificity than Han et al. found), so AMS may relate poorly to delirium.    ED health care teams also identify confusion in older adults. We included identification of confusion by any method, including implicit estimation of the treating physician and delirium detection tools. Delirium detection tools such as the 4 A’s Test (4AT) to rule in (pooled LR+ 7.5, 95% CI 2.7-20.7) and rule out (pooled LR- 0.18, 95% CI 0.09-0.34) delirium are used in some EDs. [^14^](https://paperpile.com/c/HSTcT3/Na7Mg) |
| **SUBGROUPS:** | Patients with delirium (or AMS) and clinical features (trauma, focal neurological deficit, and anticoagulation use) which are commonly thought to be associated with a clinically significant abnormality on the head CT. These subgroups were evaluated in our accompanying systematic review. [^15^](https://paperpile.com/c/HSTcT3/Vz1zp) |
| **CONFLICT OF INTERESTS:** | **Kerstin de Wit, MD:** Nothing to disclose.  **Jane M. Hayes, MD, MPH:** Nothing to disclose.  **Danya Khoujah, MBBS, MEHP:** Nothing to disclose.  **Sangil Lee MD, MS:** Nothing to disclose.  **Shan W. Liu, MD, SD:** Serves on the American College of Emergency Physicians’ Geriatric Emergency Department Accreditation Advisory Board, as well as the International Federation of Emergency Medicine Geriatric Emergency Medicine Special Interest Group.  **Alexander Lo, MD, PhD:** Nothing to disclose.  **Sangil Lee, MD, MS:** Nothing to disclose. |

# **ASSESSMENT**

| **Problem: Is obtaining a head CT in older adults with delirium a problem?** Is the problem a priority? | | |
| --- | --- | --- |
| **JUDGEMENT** | **RESEARCH EVIDENCE** | **ADDITIONAL CONSIDERATIONS** |
| ○ No ○ Probably no ○ Probably yes X Yes ○ Varies ○ Don't know | Delirium in geriatric ED patients is frequently present (6-38% of older ED patients)[^16^](https://paperpile.com/c/HSTcT3/x0VSe) though usually missed or underappreciated real-time.[^11,12,17,18^](https://paperpile.com/c/HSTcT3/htW5y+BJo18+PcchM+uFmmS) Given that the number of older adults presenting to the ED is increasing[^19,20^](https://paperpile.com/c/HSTcT3/xTHax+JP9hA) and ED visits and boarding may cause and exacerbate delirium,[^21,22^](https://paperpile.com/c/HSTcT3/4Bjq6+4ribb) we need clinical practice guidelines for evaluation of patients with delirium. Some subpopulations have clear guidelines for delirium evaluation and management (e.g. trauma, ICU patients[^23^](https://paperpile.com/c/HSTcT3/PTTZ3) but these guidelines may not be appropriate for the ED patient population or address the clinical scenarios typically confronted in the ED setting.    One study[^9^](https://paperpile.com/c/HSTcT3/fNWBf) showed that the etiology for delirium in the ED includes infection (39.5%), neurological (36.5%), metabolic (17.3%), cardiopulmonary (14/3%),  gastrointestinal (5.3%), trauma or toxicology (3.3%), and others (3.3%).    CTs are frequently (sometimes reflexively, as reported 23%) obtained in older adults with delirium.[^24^](https://paperpile.com/c/HSTcT3/2FkAJ) Tu conducted a retrospective study [^25^](https://paperpile.com/c/HSTcT3/8F7PU) of head CTs and reported the rate of abnormality was 9.8% among those with chief complaints of AMS.  The 2014 Geriatric ED guidelines do not provide recommendations for delirium and the use of imaging studies.[^26^](https://paperpile.com/c/HSTcT3/v7P4O) | There is no direct evidence from ED settings that prompt treatment of the presumptive cause of delirium or symptoms of delirium improves outcome.[^27^](https://paperpile.com/c/HSTcT3/76KR4)    The American College of Radiology states “Unless the etiology is clear and the risk of intracranial pathology is low, neuroimaging should be included in the initial assessment of recent AMS.[^28^](https://paperpile.com/c/HSTcT3/VFYJi)    Undesired effect of obtaining head CT in confused older adults, including cost, time, and need for additional sedatives to complete diagnostic tests. |
| **Test accuracy: How accurate is delirium in predicting a clinically significant abnormality on head CT as a cause of delirium?** How accurate is the test? | | |
| **JUDGEMENT** | **RESEARCH EVIDENCE** | **ADDITIONAL CONSIDERATIONS** |
| ○ Very inaccurate ○ Inaccurate ○ Accurate ○ Very accurate ○ Varies X Don't know | Our meta-analysis showed that based on two small studies [^29,30^](https://paperpile.com/c/HSTcT3/B98Ne+cb8Fu) AMS is not associated with abnormal head CT findings (OR 0.35; 95%CI 0.31- 4.0).[^7^](https://paperpile.com/c/HSTcT3/DIHj4)    The proportion of geriatric patients presenting to the ED with delirium who were found to have an abnormal head CT was 15.6% (95% CI 7.3-26.2). Results from two studies (n=506) found that the presence of a focal neuro deficit in a patient with AMS was a strong indicator for an abnormal head CT, with odds ratio (OR) of 101.8 (95% CI 30.5-340.1). Results from two studies (n=384) found that anticoagulation was not associated with abnormal head CT, with OR of 1.2 (95% CI 0.4-3.3).[^15^](https://paperpile.com/c/HSTcT3/Vz1zp) | Exposure variable of interest (delirium) was not available in studies; confusion, AMS were used as a surrogate, which may have high specificity but low sensitivity for delirium, likely contributing to misclassification of delirium.  Head CT was chosen as the outcome, rather than an MRI, due to the difficulty of obtaining MRIs in the ED, especially on patients with delirium. This comes at the expense of a decreased sensitivity. (Expert opinion)    Observed estimates of sensitivity could be artificially increased by partial verification bias, differential verification bias, imperfect gold standard bias, and spectrum bias. Concurrently, observed estimates of specificity could be reduced by partial verification bias, differential verification bias, and imperfect gold standard bias.[^31^](https://paperpile.com/c/HSTcT3/YCtNc)  It’s important to note that in the included studies, obtaining the head CT was up to the treating clinician’s discretion. Therefore, the 15.6% was the proportion of abnormal head CTs in geriatric ED patients with AMS *in whom the clinician decided to get a CT on*, and not in all-comers. Therefore, the proportion of abnormal head CTs in *all* geriatric ED patients with AMS may differ.  Extrapolating from prior data,[^24^](https://paperpile.com/c/HSTcT3/2FkAJ) less than half of older adults presenting with delirium will receive a head CT.  Based on indirect evidence on the accuracy of physical examination in identifying patients with an abnormal head CT, depending on the history or physical examination alone may not be sufficient to determine the presence of an abnormal head CT, and therefore may lead to missed abnormalities if a CT were not obtained. The data below is not specific to older adults or those with associated AMS:   - *The FAST may be inaccurate to identify a stroke:* In a study of ambulance paramedics utilizing the Face, Arm, Speech Test (FAST), the stroke diagnosis was correct in 144 of 183 (79%) stroke patients who initially presented to them.[^32^](https://paperpile.com/c/HSTcT3/DUc2b) - *The neurological examination may not be reproducible among different physicians:* Upper extremity neurological exam by occupational medicine physicians: Strength, sensibility to touch, pain and vibration, and mechanosensitivity were predominantly assessed with moderate to very good reproducibility (median kappa-values 0.54, 0.69, 0.48, 0.58, and 0.53, respectively). The reproducibility of the defined patterns was fair to excellent (median correlation coefficient = 0.75) and the overall identification of limbs with/without pattern(s) was good (kappa = 0.75).[^33^](https://paperpile.com/c/HSTcT3/NOuvv) - *The neurological examination may not be accurate for neuropathy:* Neurological exam done by occupational medicine physicians: the sensitivity, specificity, positive and negative predictive values for upper extremity neuropathy were 0.73, 0.86, 0.93 and 0.90, respectively.[^34^](https://paperpile.com/c/HSTcT3/Ipqo7) - *The diagnostic accuracy of various neurological exams in identifying a focal neurological lesion*: The positive likelihood ratios for focal neurological lesions ranged from 1.06 (pronator drift) to 22.11 (single leg stance with eyes open, while the negative likelihood ratios ranged from 0.47 (tandem gait) to 0.97 (pupil symmetry). The inter-rater reliability was generally poor, with only tandem gait showing excellent agreement (kappa 0.92).[^35^](https://paperpile.com/c/HSTcT3/t69AP) - Accuracy of identifying patients with severe head trauma is summarized in the systematic review by Easter et al.[^36^](https://paperpile.com/c/HSTcT3/6ODXF) The presence of physical examination findings suggestive of skull fracture (likelihood ratio [LR], 16; 95% CI, 3.1-59; specificity, 99%), GCS score of 13 (LR, 4.9; 95% CI, 2.8-8.5; specificity, 97%), 2 or more vomiting episodes (LR, 3.6; 95% CI, 3.1-4.1; specificity, 92%), any decline in GCS score (LR range, 3.4-16; specificity range, 91%-99%;), and pedestrians struck by motor vehicles (LR range, 3.0-4.3; specificity range, 96%-97%) were associated with severe intracranial injury on CT. Among patients with apparent minor head trauma, the absence of any of the features of the Canadian CT Head Rule (≥65 years; ≥2 vomiting episodes, amnesia >30 minutes, pedestrian struck, ejected from vehicle, fall >1 m, suspected skull fracture, or GCS score <15 at 2 hours) had an LR of 0.04 (95% CI, 0-0.65), lowering the probability of severe injury to 0.31% (95% CI, 0%-4.7%). The absence of all the New Orleans Criteria findings (>60 years, intoxication, headache, vomiting, amnesia, seizure, or trauma above the clavicle) had an LR of 0.08 (95% CI, 0.01-0.84), lowering the probability of severe intracranial injury to 0.61% (95% CI, 0.08%-6.0%). - *Accuracy of identifying patients who require neuroimaging for headache* is summarized in the systematic review by Detsky et al. [^37^](https://paperpile.com/c/HSTcT3/0ox7D). They reported that the best predictors can be summarized by the mnemonic POUNDing (Pulsating, duration of 4-72 hOurs, Unilateral, Nausea, Disabling). If 4 of the 5 criteria are met, the likelihood ratio (LR) for definite or possible migraine is 24 (95% confidence interval [CI], 1.5-388); if 3 are met, the LR is 3.5 (95% CI, 1.3-9.2), and if 2 or fewer are met, the LR is 0.41 (95% CI, 0.32-0.52). For the neuroimaging question, several clinical features were found on pooled analysis to predict the presence of a serious intracranial abnormality: cluster-type headache (LR, 10.7; 95% CI, 2.2-52); abnormal findings on neurologic examination (LR, 5.3; 95% CI, 2.4-12); undefined headache (ie, not cluster-, migraine-, or tension-type) (LR, 3.8; 95% CI, 2.0-7.1); headache with aura (LR, 3.2; 95% CI, 1.6-6.6); headache aggravated by exertion or a valsalva-like maneuver (LR, 2.3; 95% CI, 1.4-3.8); and headache with vomiting (LR, 1.8; 95% CI, 1.2-2.6). No clinical features were useful in ruling out significant pathologic conditions. - *Accuracy of identifying patient taking anticoagulation*: Per ED/hospital clinicians, the use of any anticoagulant or antiplatelet agent was identified in 595 (28.2%) patients. Kappa statistics between EMS and ED/hospital clinicians for the specific agents were: 0.76 (95% CI 0.71–0.82) for warfarin, 0.45 (95% CI 0.19–0.71) for DOAC agents. EMS and ED/hospital clinicians have acceptable agreement with preinjury warfarin.[^38^](https://paperpile.com/c/HSTcT3/5hCHp) - Another study showed that exact agreement was 87%, Cohen's kappa 0.66 (95% CI, 0.63-0.72), sensitivity 84.0% (95% CI, 79.3%-83.8%), and specificity 87.6% (95% CI, 85.1%-89.7%) for anticoagulant medication use. Patient-reported outcome and exposure data were unreliable in this study. [^39^](https://paperpile.com/c/HSTcT3/eSGLE) |
| **Desirable Effects:**  How substantial are the desirable anticipated effects?  **In other words, if this were true, How substantial are the desirable anticipated effects of using acute delirium to identify patients with acute clinically significant abnormality on head CT?** | | |
| **JUDGEMENT** | **RESEARCH EVIDENCE** | **ADDITIONAL CONSIDERATIONS** |
| ○ Trivial ○ Small X Moderate ○ Large ○ Varies ○ Don't know | Head CTs are ordered on a large proportion of pts with AMS (44% as per Nesselroth et al.[^29^](https://paperpile.com/c/HSTcT3/B98Ne)), although the yield, defined as the positive predictive value, is relatively low. Missed acute intracranial cause of AMS can lead to preventable poor outcomes (e.g. missed intracranial bleed).Ordering CTs on all the patients with AMS who are most likely to benefit from expedited recognition and intervention would improve prognosis if acute intracranial abnormalities are diagnosed and intervened upon promptly.    Concurrently, it would be similarly beneficial to identify patients who are confused but would not likely benefit from a head CT, however our reviews have not identified any relevant study.[^7,15^](https://paperpile.com/c/HSTcT3/DIHj4+Vz1zp) Focusing on obtaining imaging on only a subpopulation of patients with delirium will decrease unnecessary testing, cost and length of stay in the ED.[^40^](https://paperpile.com/c/HSTcT3/ND4dO) | Lai et al. reported that true positive CT ﬁndings were present in 29 of 200 patients with a yield of 14.5%. Thirteen patients had ischaemic strokes, seven had subdural hemorrhage (SDH) with or without SAH and nine had intracerebral hemorrhage. [^41^](https://paperpile.com/c/HSTcT3/FCOfH) We were unable to identify what % required emergency intervention or any intervention.    There is no evidence on morality benefit or disease oriented outcome in the literature.    The desired effect would be those individuals with AMS most likely to benefit from a CT receive a timely CT, while those unlikely to benefit from a CT do not receive a CT.    Nesselroth[^29^](https://paperpile.com/c/HSTcT3/B98Ne) did not have data on outcomes among delirious patients or among delirious patients, nor did Segard.[^30^](https://paperpile.com/c/HSTcT3/cb8Fu) |
| Undesirable Effects: How substantial are the undesirable anticipated effects of using delirium to identify patients with acute clinically significant abnormality on head CT as cause of altered mental status? | | |
| **JUDGEMENT** | **RESEARCH EVIDENCE** | **ADDITIONAL CONSIDERATIONS** |
| ○ Large ○ Moderate ○ Small ○ Trivial ○ Varies X Don't know | Direct evidence:  Clinicians report the challenge of establishing change in mental status if there is no family member or report from a nursing home regarding a patient’s baseline status. [^42^](https://paperpile.com/c/HSTcT3/KBCfI)  Evaluating a patient for delirium could take more time than is currently spent with a patient meaning physicians might see fewer patients/shifts.    Physicians may not accurately or reliably identify delirium.[^11,12,43^](https://paperpile.com/c/HSTcT3/htW5y+BJo18+ezCRr)    Identifying AMS by using the single question in delirium (SQiD) with patient and caregiver has sensitivity of 44% (95%CI 41-80), specificity of 87% (95%CI 74-95) in the oncology inpatient. No significant undesirable effect was seen.[^44,45^](https://paperpile.com/c/HSTcT3/vxYKd+uZRbQ)    Inaccurate recognition of AMS among clinicians, its diagnostic characteristics for delirium and etiology for delirium may lead to an increase in ordering neuroimaging, with subsequent delays in care for the individual patient and entire ED, and increase ED length of stay and boarding (expert opinion).  We evaluated the accuracy of history and physical exam findings of focal neurological deficit, trauma (if there is any), headache (if there is any), and the use of anticoagulation. None of these studies contemplated or reported undesirable effects of leveraging findings on exams to guide CT decision-making.    Harms of missing AMS and delirium lead to sedation in patients with hyperactive delirium, delay in care and increase ED length of stay, which worsens delirium, and transport to another facility for rural EDs. Several studies are cited below.    A systematic review (not restricted to age 65 or above) suggested [^46^](https://paperpile.com/c/HSTcT3/KZ4QN) that the safety profile of lorazepam, alone or in combination. Dizziness was reported in ≥10% of patients in 3 trials. Sedation/somnolence was documented in about 10% of patients in three trials.    The link between increased ED length of stay and risk of delirium has been reported in the literature.[^47^](https://paperpile.com/c/HSTcT3/0uk2t) The odds of delirium was 2.23 (95%CI 1.13 to 4.41) for those who spent more than 10 hours in the ED.    There was no study evaluating the ED to ED transfer and the risk of delirium. One study on the room to room transfer showed an increased odds of delirium(OR 9.69, 95%CI 6.20-15.16) from hospital-based case-control study.[^48^](https://paperpile.com/c/HSTcT3/xwAvz) | Improving the positive predictive value of head CT by choosing the right type of patients needs to be weighed against reduction of cost of fewer CTs ordered, minimizing harm with sedation and ED LOS to complete the test. (Expert opinion)    False negative head CTs could lead to premature closure for evaluation of etiology for delirium.[^49^](https://paperpile.com/c/HSTcT3/gRv2O)    Based on indirect evidence on the accuracy of physical examination in identifying patients with an abnormal head CT, depending on the history or physical examination alone may not be sufficient to determine the presence of an abnormal head CT, and therefore may lead to missed abnormalities if a CT was not obtained. The data is detailed above in the “Test Accuracy” section.  Incidental findings: Rate of incidental findings among 682 CT scans was 228/682 (33.4%) Of the 405 head CT scans completed, 19.8% had incidental findings.[^50^](https://paperpile.com/c/HSTcT3/zdEyt) |
| **Certainty of the evidence of test accuracy: How certain are we of the results above (i.e. accuracy of delirium in predicting an acute clinically significant abnormality on head CT as a cause of delirium)?** What is the overall certainty of the evidence of test accuracy? | | |
| **JUDGEMENT** | **RESEARCH EVIDENCE** | **ADDITIONAL CONSIDERATIONS** |
| X Very low ○ Low ○ Moderate ○ High ○ No included studies | Overall, there was very low certainty of the evidence of test accuracy. The two studies included in the meta-analysis examining whether change in mental status predicted an abnormal head CT had largely high to unclear risk of bias.[^7^](https://paperpile.com/c/HSTcT3/DIHj4)  For the ROB analysis regarding proportion of abnormal head CT’s among confused patients, there was variable ROB in the studies included in our systematic review.[^15^](https://paperpile.com/c/HSTcT3/Vz1zp) | Lai et al did an inpatient study of 200 patients admitted to a delirium unit who had head CT, 14.5% (29) had acute intracranial pathology accountable for the delirium.[^41^](https://paperpile.com/c/HSTcT3/FCOfH) This rate shows that the reported rate of abnormality is similar in the ED, adding the external validation.  An additional systematic review of the yield of head CTs in adults with delirium/AMS was identified and added to the EtD, regarding “Certainty of the evidence of test accuracy”, which included ED and admitted patients. The yield for head CT in the Ed/inpatient was 13% (95% CI: 10.2%–15.9%) with considerable heterogeneity. Yieldof CT head diminished after year 2000, implying an increase in unnecessary CTs. Older age (with different cut-offs throughout the studies) was identified as a predictor of abnormal head CT as was presence of focal neurological deficit[^51^](https://paperpile.com/c/HSTcT3/Nqsj2). |
| **Certainty of the evidence of test's effects (that a head CT will be obtained)** What is the overall certainty of the evidence for any critical or important direct benefits, adverse effects or burden of the test? | | |
| **JUDGEMENT** | **RESEARCH EVIDENCE** | **ADDITIONAL CONSIDERATIONS** |
| ○ Very low ○ Low ○ Moderate ○ High X No included studies | Direct evidence is limited over all about certainty of the test’s effect. An older study by Naughton, of 333 ED patients with delirium/impaired consciousness/impaired cognition >70 years, found that 23.7% had a head CT but provided no assessment of outcomes associated with the head CT. [^24^](https://paperpile.com/c/HSTcT3/2FkAJ) | Emergency physicians miss the diagnosis of delirium in nearly two-thirds of patients with delirium.[^11,12^](https://paperpile.com/c/HSTcT3/htW5y+BJo18) Suffoletto et al found the identification rates of delirium among older ED patients among attending EPs was 8/24 vs. the 24 identified by the trained researchers.[^43^](https://paperpile.com/c/HSTcT3/ezCRr)      Any evaluation quantifying the value and effect of head CT to evaluate suspected or confirmed delirium is dependent on the accurate identification of delirium and non-delirium cases. Challenges to routinely detecting delirium t or even assessing baseline mental status include clinician time, family availability, language barriers, incomplete information from nursing homes, and reliability of sources.[^42^](https://paperpile.com/c/HSTcT3/KBCfI)    There is indirect evidence that between 44% and 60% of patients had head CTs though not limited to older ED patients and the desirable or undesirable effects associated with that advanced imaging is not typically reported. (Leong studied 967 patients >18 years, 60% older than 65, 674 (70%) had a Head CT).[^52^](https://paperpile.com/c/HSTcT3/bSuml)    Lim did study of 578 patients > 15 years of age who had change in mental status and 56.6% had head ct.  Non ED studies  In a small study of 92 hospitalized patients, of delirious older patients, 44% had head imaging. [^53^](https://paperpile.com/c/HSTcT3/yYda7) |
| **Certainty of the evidence of management's effects: How certain are we of the effects of treatment of acute clinically significant abnormality found on head CT?** What is the overall certainty of the evidence of effects of the management that is guided by the test results? | | |
| **JUDGEMENT**   ○ Very low  X Low  ○ Moderate  ○ High  ○ No included studies | **RESEARCH EVIDENCE** Low certainty for standard, uniform approach to abnormal head CT in confused older patients. Head CT findings and patient function/condition impact management decisions.  Direct evidence: None | **ADDITIONAL CONSIDERATIONS**Indirect evidence does exist highlighting variable management effects depending on condition, comorbidities, and baseline patient function   **Acute stroke**: Strokes more common in geriatric patients. Management is complex, specialized and time-critical and delivered in stroke centers.[^54^](https://paperpile.com/c/HSTcT3/XHTwj)    **ICH**: Variable depending on size, age of patient, clinical presentation in terms of if traumatic, aneurysmal, amyloid. Per Sahni [^4,55^](https://paperpile.com/c/HSTcT3/i3Po6+e99XN) no interventions have demonstrable benefit.    **Brain mass**: No standard therapy for older patients with primary brain mass.[^5,56^](https://paperpile.com/c/HSTcT3/pIJaL+ziU3U)    **Trauma**: Among 220 hospital patients with delirium who had head CT without fall, head trauma, or neurologic deficit who had CT,6 (2.7%) identified a traumatic brain injury and 4 (1.8%) were equivocal.  All traumatic brain injury or other abnormal CT findings or equivocal findings had changes in management.[^57^](https://paperpile.com/c/HSTcT3/3LP8q) |
| **Certainty of the evidence of test result/management: How certain are we that identification of delirium would lead to head CT and then lead to management of CT findings.** How certain is the link between test results and management decisions? | | |
| **JUDGEMENT** | **RESEARCH EVIDENCE** | **ADDITIONAL CONSIDERATIONS** |
| ○ Very low ○ Low ○ Moderate ○ High X No included studies | To answer this question, requires multiple steps:    1) Evidence that ED clinicians assess for delirium accurately and reliably, then  2) For patients with delirium, what certainty is there ED clinicians obtain head CT, then  3) How consistently do ED clinicians recognize subsets of delirium patients with abnormal head CT findings likely to benefit from a linked action, engage appropriate consultant services in a timely fashion (Neurology, Neurosurgery, Oncology) and evaluate patient-centered outcomes to assess whether benefits outweigh harms    Direct evidence: No included studies that link all 3 steps directly. | Indirect evidence:  1) Emergency physicians do not reliably assess delirium. Emergency physicians were noted to miss the diagnosis of delirium in nearly two-thirds of patients with delirium.[^11,12^](https://paperpile.com/c/HSTcT3/htW5y+BJo18) Suffoletto et al found the identification rates of delirium among older ED patients among attending EPs was 8/24 vs. the 24 identified by the trained researchers.[^43^](https://paperpile.com/c/HSTcT3/ezCRr)    2) Emergency physician CT head use varies between physicians, with head CTs ordered for 4% to 17% of all emergency patients.[^58^](https://paperpile.com/c/HSTcT3/mdbRA) Altered mental status is known to be a predictor of stroke, intracranial bleeding and cerebral tumor [^59^](https://paperpile.com/c/HSTcT3/pgOaN) and CT use is common, especially among older adults (25% of older adult ED visits involved a CT scan in 2007 according to Larson.[^60^](https://paperpile.com/c/HSTcT3/qO6Gj) Although there are no studies evaluating the association between emergency physician recognition of altered mental status and ordering a head CT scan, it would not be an unusual practice pattern.    Naughton, of 333 ED patients with delirium/impaired consciousness/impaired cognition >70 years, found that 23.7% had a head CT.[^24^](https://paperpile.com/c/HSTcT3/2FkAJ)    There is little research reporting on real-time management of abnormal head CT findings, however older adults receive similar benefit from stroke treatments as younger adults.[^61^](https://paperpile.com/c/HSTcT3/f7cB7) Intracranial bleeding has a high mortality rate in older adults, [^62^](https://paperpile.com/c/HSTcT3/pwsyl) so early diagnosis is important to ensure timely intervention. |
| **Certainty of effects**  What is the overall certainty of the evidence of effects of the test? | | |
| **JUDGEMENT** | **RESEARCH EVIDENCE** | **ADDITIONAL CONSIDERATIONS** |
| ○ Very low ○ Low ○ Moderate ○ High X No included studies | No direct evidence    No research has studied the benefits and harms from linking head CT scanning to delirium in the ED. The discussion is complex because some patients with delirium will require sedation to enable CT scanning, CT scanning causes delays in the ED, and sometimes, patients have to be transferred to another facility for CT scanning (all of which could cause harm to the patient). | The question is nuanced. For example, in abdominal pain, CT for all older adults does change management for most patients.[^63^](https://paperpile.com/c/HSTcT3/5s7KR) |
| **Values: Is there important uncertainty about or variability in how much stakeholders value diagnosing acute clinical intracranial abnormality as the cause of delirium?** Is there important uncertainty about or variability in how much people value the main outcomes? | | |
| **JUDGEMENT** | **RESEARCH EVIDENCE** | **ADDITIONAL CONSIDERATIONS** |
| ○ Important uncertainty or variability ○ Possibly important uncertainty or variability ○ Probably no important uncertainty or variability X No important uncertainty or variability | **Patient Values**  Rodriguez et al provided indirect evidence that nearly all trauma patients (desire 91.2%, 95% CI [89.4-93.1]) preferred exposure to radiation to detect acute life-threatening traumatic injuries (LTI) when the odds of LTI was 25%.[^64^](https://paperpile.com/c/HSTcT3/TUIiK) Preferences for receiving CT decreased accordingly when the risk of LTI fell as follows: LTI 10% (desire 79.3%, 95% CI [76.7–81.9]), LTI 5% (desire 69.1%, 95% CI [66.1–72.1]) and LTI <2% (desire 53.8%, 95% CI [50.6–57.0]).    The care partner representative in our subgroup believes/advocated value in knowing if they are going to be ok if they are going home. Important that option is there if clinicians feel that CT scans will help identify any issues. Patient perspective is wanting to know, will affect quality of life. This is in alignment with the sentiment expressed by individuals who were surveyed/interviewed by a member of our group as part of a qualitative study on falls patients. A strong theme was wanting to know whether they had intracranial bleeding (de Wit, personal communication. | **Clinician Values**  ED clinician brain CT imaging decisions may be influenced by clinical decision support rules, patient out-of-pocket cost information and findings from malpractice case review.[^65^](https://paperpile.com/c/HSTcT3/R2UPw)    Abnormal CT scans are inherently valuable as they provide concrete answers to clinicians and patients about the cause of the change in mental status. In some cases, abnormal CT findings can lead to emergent procedures and treatment. However, normal and non-diagnostic CT scans are also valuable as they may be able to assuage worries that family members may have about intracranial abnormalities. A normal or non-diagnostic head CT can help guide a clinician to initiate a broader workup for the cause of an acute change in mental status. A normal or non-diagnostic head CT can also be key to helping a clinician feel confident that they have ruled out emergent causes of AMS and are appropriately managing their patient.  Overall, what matters to the patient and their family or care partners is an essential compass to guide individualized decision-making around CT imaging. [^66–68^](https://paperpile.com/c/HSTcT3/V2pGg+voBWP+qfazI) Patients/family may understand if it is explained to them that diagnosing ICH may mean surgery. If that is not within goals of care, then give the family the option to avoid head CT. Other factors including unique patient circumstance, comorbid illness burden, cognitive capacity, and frailty may also impact decisional capacity and values. [care partner representative input]    On the other hand, a U-shaped curve of testing exists in emergency medicine – where doing more improves care initially, then there is no benefit and ultimately, doing more is harmful to patients.[^69^](https://paperpile.com/c/HSTcT3/439XY) |
| **Balance of effects: Does the balance between desirable and undesirable effects of using acute delirium to identify patients with acute clinically significant abnormality favor using this feature to obtain a head CT or not?** Does the balance between desirable and undesirable effects favor the intervention or the comparison? | | |
| **JUDGEMENT** | **RESEARCH EVIDENCE** | **ADDITIONAL CONSIDERATIONS** |
| ○ Favors the comparison ○ Probably favors the comparison ○ Does not favor either the intervention or the comparison ○Probably favors the intervention ○ Favors the intervention ○ Varies X Don't know | Our systematic review found that the proportion of older ED patients with delirium, using AMS and confusion as surrogates, who were found to have an abnormal head CT was 15.6% (95% CI 7.3-26.2%). We believe that the possibility of finding an intracranial abnormality in 15% of geriatric patients who present to the ED with AMS is clinically important.[^15^](https://paperpile.com/c/HSTcT3/Vz1zp)    Using AMS as our screening tool rather than delirium has both advantages and disadvantages. This approach is sensitive [^70^](https://paperpile.com/c/HSTcT3/5WZaw) and broad enough to catch all patients with delirium, but will likely exclude many with delirium. |  |
| **Resources required: How large are the costs of obtaining a head CT on older adults with acute AMS in the ED?** How large are the resource requirements (costs)? | | |
| **JUDGEMENT** | **RESEARCH EVIDENCE** | **ADDITIONAL CONSIDERATIONS** |
| ○ Large costs X Moderate costs ○ Negligible costs and savings ○ Moderate savings ○ Large savings ○ Varies ○ Don't know | **Direct cost:**  Delirium is the underlying reason for about 1.5 million ED visits annually in the US.[^16^](https://paperpile.com/c/HSTcT3/x0VSe) Delirium is only a subset of all ED presentations for altered mental status, therefore the number of patients with acute AMS is much higher. Therefore, the cost of obtaining a head CT on all older adults in the ED with acute AMS would be substantial. However, this practice is already common.[^24^](https://paperpile.com/c/HSTcT3/2FkAJ) Therefore, our recommendation FOR obtaining CT would likely not incur a substantial additional cost. However, a recommendation AGAINST would likely be associated with cost savings.    The cost of obtaining a CT (and reading it) ~ $1700[^71^](https://paperpile.com/c/HSTcT3/Xv1zS) (from a cost-effectiveness study in patients with minor head trauma, costs were in 2005 $US). Cost of head CT in the ED was $763, which was 3 times more expensive than routine head CT. [^72^](https://paperpile.com/c/HSTcT3/ix2oE)    **Indirect cost:**  Length of stay: For the past two years during the COVID-19 pandemic, ED boarding has increased. [^73^](https://paperpile.com/c/HSTcT3/QRW2T) Increasing the number of diagnostic tests may further increase ED wait times and lengths of stay.[^74^](https://paperpile.com/c/HSTcT3/vWagJ)). Boarding in the ED has been associated with increased mortality. [^75^](https://paperpile.com/c/HSTcT3/DFEi2) A study revealed a net increase in ED length of stay (ED LOS) of 5 hours from obtaining neuroimaging for bizarre behavior. [^76^](https://paperpile.com/c/HSTcT3/PU9Uq) In that study, 85% of patients were referred to a consultant, 92% were to psychiatry in the ED.    Financial costs to patients: The costs of the imaging is passed along to patients. This cost is unfairly burdensome to patients of lower socioeconomic status.    Medical costs to patients: Older patients with AMS may require sedation to safely and effectively complete CT imaging. The sedation required for imaging could worsen their mental status and lead to a worse recovery. Cost of sedation - study of sedation for pediatrics imaging in Japan. 74.2-92.7 USD for intravenous sedation by non-anesthesiologists, 112.1-458.3 USD for oral or rectal sedation by non-anesthesiologists.[^77^](https://paperpile.com/c/HSTcT3/MWnbw)    Need for sedation and further delay of care: anecdotally happens often. Some case reports for sedation for neuro causes causing delays of care.[^78^](https://paperpile.com/c/HSTcT3/4uBko)    Cost of transport if CT is not available: average EMS transfer cost of $700 for ground and $5800 for air. [^79^](https://paperpile.com/c/HSTcT3/tETIG) Ward estimated that avoiding rural transfers save $2673.[^80^](https://paperpile.com/c/HSTcT3/Wtdd7)    CT may identify incidental inconsequential findings which might lead to additional unnecessary testing. |  |
| **Certainty of evidence of required resources (above)** What is the certainty of the evidence of resource requirements (costs)? | | |
| **JUDGEMENT** | **RESEARCH EVIDENCE** | **ADDITIONAL CONSIDERATIONS** |
| ○ Very low ○ low  ○ Moderate[8]  ○ High X No included studies | Cost around CT scans, increased LOS need to be considered.    Need for sedation is based on expert opinion/anecdotal. |  |
| **Cost effectiveness: Does the cost-effectiveness of using delirium alone as reason to obtain head ct to determine an acute intracranial lesion as cause of his/her delirium favor the intervention or comparison.** Does the cost-effectiveness of the intervention favor the intervention or the comparison? | | |
| **JUDGEMENT** | **RESEARCH EVIDENCE** | **ADDITIONAL CONSIDERATIONS** |
| ○ Favors the comparison ○ Probably favors the comparison ○ Does not favor either the intervention or the comparison ○ Probably favors the intervention ○ Favors the intervention ○ Varies X No included studies | No direct evidence explored or quantified cost-effectiveness for using delirium as a trigger for routine CT imaging. | Indirect Evidence  Cost-effectiveness of obtaining a head CT in minor head injury in older adult (vs admission for observation for 24 h): less expensive.[^71^](https://paperpile.com/c/HSTcT3/Xv1zS)    The presence of acute AMS is not clearly associated with an acute abnormal clinically significant finding on CT  CTs are expensive (both directly and indirectly)  BUT  The proportion of older adults with acute AMS with abnormal clinically significant findings on CT neuroimaging is 15.6% (95% CI 7.3-26.2).[^15^](https://paperpile.com/c/HSTcT3/Vz1zp) |
| **Health Equity**What would be the impact on health equity? | | |
| **JUDGEMENT** | **RESEARCH EVIDENCE** | **ADDITIONAL CONSIDERATIONS** |
| ○ Reduced ○ Probably reduced ○ Probably no impact ○ Probably increased ○ Increased ○ Varies X Don't know | No direct evidence: No identified articles on equity. But if physicians could accurately identify delirium in all older ED patients, and if the finding of delirium led to a head CT scan, it may avoid current inequities described below.It is unclear whether language is a barrier to accurately identifying delirium in older ED patients.  Indirect evidence from studies of missed diagnoses of stroke in the U.S. EDs demonstrated significant inequity across socio demographic groups: In a 2009 analysis of state-wide administrative data from 9 states, the odds of a missed diagnosis of ischemic and hemorrhagic stroke was higher for women, persons younger than 45 years, Blacks, Asian-Pacific Islanders and Hispanics (compared to Whites), and patients presenting to either non-teaching hospitals or small-volume hospitals.[^81^](https://paperpile.com/c/HSTcT3/hfzFr)  Analysis of 2006-2016 data from NHAMCS found that women, minority race (Blacks OR=0.78, Hispanics OR=0.94 or Asians OR=0.82, compared with whites), rural ED location and uninsured status were less likely to receive imaging studies in general. In particular, Blacks or Hispanics compared with Whites were less likely to receive CT or MR imaging.[^82^](https://paperpile.com/c/HSTcT3/ETImF) | As delirium is missed in 2/3rds of ED cases with language concordance, delirium is likely missed when there is language discordance.[^83^](https://paperpile.com/c/HSTcT3/a9IXg)  Historically, ED delirium health inequities across gender/sex, race/ethnicity, persons living with dementia, primary language, and rurality have not been transparently reported or explored.[^84^](https://paperpile.com/c/HSTcT3/HGSi9) |
| **Acceptability: Is using delirium as the reason to obtain a head CT acceptable to key stakeholders (as listed above)?** Is the intervention acceptable to key stakeholders? | | |
| **JUDGEMENT** | **RESEARCH EVIDENCE** | **ADDITIONAL CONSIDERATIONS** |
| ○ No ○ Probably no X Probably yes ○ Yes ○ Varies ○ Don't know | No published data on this exact question. The care partner representative in our group stated that patients preferred knowing if there were any concerning findings on the head CT.  Physicians/practitioners clearly accept advanced imaging with head CT as a part of routine.    Hospital systems/payors: We do not have data on how medicare reimburses for this  CMS code “R41.82- Altered mental status, unspecified” under the ICD-10 codes that support medical necessity.[^85^](https://paperpile.com/c/HSTcT3/uuHaB) | CT is acceptable to patients/families, as it is a familiar intervention that they are aware of.  However, obtaining a CT on a non-cooperative patient may be logistically difficult and administering sedation in order to achieve that may be less acceptable.  With agitated patients with delirium and dementia finding out "what matters" most to the patient and the caregiver is needed so communication and education to patients, family, and caregivers can help make these decisions regarding sedating and getting a CT scan. [care partner representative perspective] |
| **Feasibility** Is the intervention feasible to implement? | | |
| **JUDGEMENT** | **RESEARCH EVIDENCE** | **ADDITIONAL CONSIDERATIONS** |
| ○ No ○ Probably no ○ Probably yes ○ Yes X Varies ○ Don't know | No direct evidence explored the feasibility of using delirium as a trigger for routine CT imaging.  **CT Feasibility**  In a study of a sample of U.S. EDs in the NEDI database, 96% have access to a CT scanner and with only 1% within this group not having 24-hour access to the scanner; 86% have access to either an on-site or mobile MRI and among these 39% have an on-site or on-call MR technician.[^86^](https://paperpile.com/c/HSTcT3/rYdg0)  15% of Canadian rural EDs have access to a CT scanner indicating challenges and potentially health inequities for patients in the rural setting. [^87^](https://paperpile.com/c/HSTcT3/mkVjb) | **Delirium Identification Feasibility**  As noted above, delirium is routinely missed in ED settings [^11,12^](https://paperpile.com/c/HSTcT3/htW5y+BJo18) and emergency medicine nurses/physicians have noted multiple obstacles to protocolized delirium screening or detection initiatives[^42,88–92^](https://paperpile.com/c/HSTcT3/lJnTD+KBCfI+LoFfQ+cktJu+FWqKx+hXxtt) which raises concerns about the feasibility of leveraging delirium recognition as a trigger for impactful CT imaging. |

# **SUMMARY OF JUDGEMENTS**

|  | **JUDGEMENT** | | | | | | |
| --- | --- | --- | --- | --- | --- | --- | --- |
| **PROBLEM** | No | Probably no | Probably yes | **Yes** |  | Varies | Don't know |
| **TEST ACCURACY** | Very inaccurate | Inaccurate | Accurate | Very accurate |  | Varies | **Don't know** |
| **DESIRABLE EFFECTS** | Trivial | Small | **Moderate** | Large |  | Varies | Don't know |
| **UNDESIRABLE EFFECTS** | Large | Moderate | Small | Trivial |  | Varies | **Don't know** |
| **CERTAINTY OF THE EVIDENCE OF TEST ACCURACY** | **Very low** | Low | Moderate | High |  |  | No included studies |
| **CERTAINTY OF THE EVIDENCE OF TEST'S EFFECTS** | Very low | Low | Moderate | High |  |  | **No included studies** |
| **CERTAINTY OF THE EVIDENCE OF MANAGEMENT'S EFFECTS** | Very low | **Low** | Moderate | High |  |  | No included studies |
| **CERTAINTY OF THE EVIDENCE OF TEST RESULT/MANAGEMENT** | Very low | Low | Moderate | High |  |  | **No included studies** |
| **CERTAINTY OF EFFECTS** | Very low | Low | Moderate | High |  |  | **No included studies** |
| **VALUES** | Important uncertainty or variability | Possibly important uncertainty or variability | Probably no important uncertainty or variability | **No important uncertainty or variability** |  |  |  |
| **BALANCE OF EFFECTS** | Favors the comparison | Probably favors the comparison | Does not favor either the intervention or the comparison | Probably favors the intervention | Favors the intervention | Varies | **Don't know** |
| **RESOURCES REQUIRED** | Large costs | **Moderate costs** | Negligible costs and savings | Moderate savings | Large savings | Varies | Don't know |
| **CERTAINTY OF EVIDENCE OF REQUIRED RESOURCES** | Very low | Low | Moderate | High |  |  | **No included studies** |
| **COST EFFECTIVENESS** | Favors the comparison | Probably favors the comparison | Does not favor either the intervention or the comparison | Probably favors the intervention | Favors the intervention | Varies | **No included studies** |
| **EQUITY** | Reduced | Probably reduced | Probably no impact | Probably increased | Increased | Varies | **Don't know** |
| **ACCEPTABILITY** | No | Probably no | **Probably yes** | Yes |  | Varies | Don't know |
| **FEASIBILITY** | No | Probably no | Probably yes | Yes |  | **Varies** | Don't know |

# **TYPE OF RECOMMENDATION**

| Strong recommendation against the intervention | Conditional recommendation against the intervention | Conditional recommendation for either the intervention or the comparison | Conditional recommendation for the intervention | Strong recommendation for the intervention |
| --- | --- | --- | --- | --- |
| ○ | ○ | X | ○ | ○ |

# **CONCLUSIONS**

| **Recommendation** |
| --- |
| In the undifferentiated older ED patients with delirium or altered mental status, we have insufficient data to recommend obtaining a head CT vs not obtaining a head CT. [Conditional recommendation for either the intervention or the comparison, very low certainty of evidence]. |
|  |
| **Justification** |
| We downgraded our recommendation based upon indirectness and imprecision. Indirectness was a consequence of AMS as a surrogate for delirium and imprecision because of the lack of an estimate of management effect linked to identification of delirium to focus head CT imaging decisions or outcomes. Our systematic review did not show AMS predicted abnormal head CT,[^7^](https://paperpile.com/c/HSTcT3/DIHj4) and studies were small with variable risk of bias. Among patients who did have confusion or AMS and had head CT, approximately 15% had an acute abnormality on their scan.[^15^](https://paperpile.com/c/HSTcT3/Vz1zp) However, cost and resources were certainly higher to undergo head CT while the certainty of the evidence of the test results management were low.  Although 15% appears important, there is a high risk of spectrum bias towards sicker subsets of the ED population given the studies included patients who the clinician chose to obtain a head CT on, not in all those with AMS. Therefore, the true proportion of CTs that alter management decisions and patient-oriented outcomes is unknown, and is likely much lower than observed 15% if CTs were obtained indiscriminately on all patients with delirium. Overall, direct evidence is absent to generate number needed to treat by obtaining one CT on an individual with delirium to improve outcome (delirium severity or duration, morbidity, mortality) who would otherwise not have been treated. |

| **Subgroup considerations** |
| --- |
| Patients with focal neurological deficits are more likely to have an abnormal head CT than those without, therefore we would recommend obtaining a head CT in this subgroup. Whether the patient is on anticoagulation or not does not significantly change the proportion of abnormal head CT in this population, therefore, this does not change our recommendation in this subgroup.  There is insufficient data to make any specific consideration for patients presenting with a headache. [^15^](https://paperpile.com/c/HSTcT3/Vz1zp) |
| **Implementation considerations** |
| Head CTs for altered mental status, delirium or confusion are common. We sought to determine if we could safely decrease head CT utilization for unnecessary cases but we did not find evidence to do so.  An important consideration regarding obtaining a head CT in patients with AMS would be the timing of the head CT itself, whether in the ED as an inpatient. Unfortunately, current data does not clarify this distinction.  In considering a future state in which sufficiently high quality evidence exists to inform diagnostic imaging decisions, clinical practice guideline teams will need to consider the Knowledge Translation Pipeline to accelerate uptake of these recommendations (below).  **Knowledge Translation Pipeline**[^93^](https://paperpile.com/c/HSTcT3/VxQOP)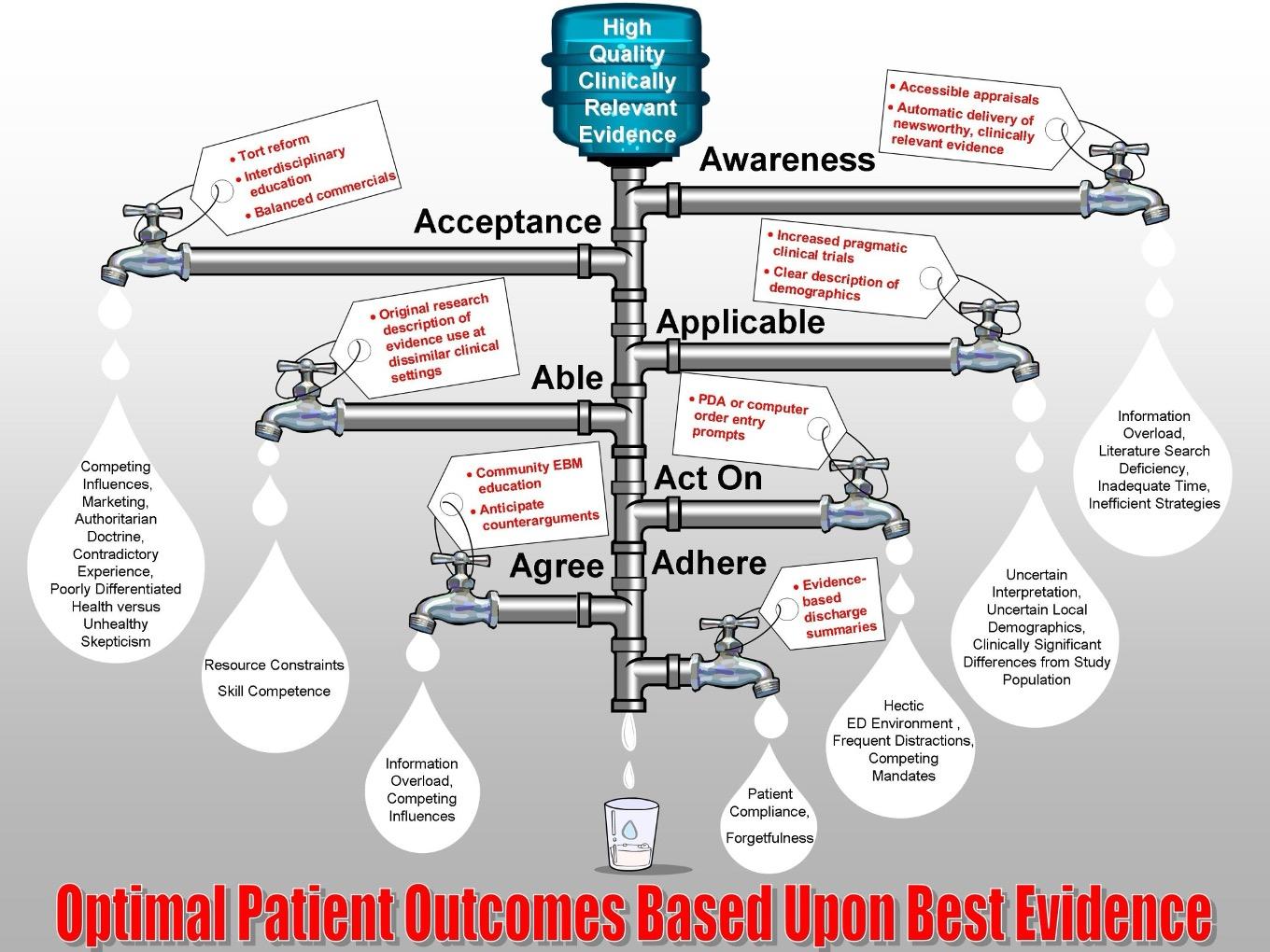 |

[^93^](https://paperpile.com/c/HSTcT3/VxQOP)

| **Monitoring and evaluation** |
| --- |
| Not applicable given no actionable recommendations. |
| **Research priorities** |
| 1. Higher quality research that minimizes diagnostic biases like spectrum bias and partial verification bias quantifying whether older patients diagnosed with delirium in the ED are more likely to have abnormal head CT compared to those without delirium are needed. (and number needed to test)  2. Identifying predictors associated with an abnormal head CT among older ED patients with delirium are needed.  3. Exploring the association between ED identification of delirium, clinician head CT order, time-dependent actionability of abnormal head CT, and patient-oriented outcomes are needed to more confidently qualify the link between test (delirium recognition & CT) and management and derive quantitative estimates of Number Needed to Treat with delirium recognition guided CT to attain improved outcomes that otherwise would not have occurred.  4. Research exploring the association with ED delirium triggered head CT with quality of life outcomes are needed.  5. If subsets of ED older adults with delirium who would benefit from head CT are identified, cost-benefit analyses from the perspective of payers, institutions, and society would be needed.  6. If subsets of ED-older adults with delirium who would benefit from head CT and this approach is cost-effective, efficacious implementation strategies would need to be identified to scale up these approaches. |

**References**

1. [Hénon H, Lebert F, Durieu I, et al. Confusional state in stroke: Relation to preexisting dementia, patient characteristics, and outcome. *Stroke*. 1999;30(4):773-779.](http://paperpile.com/b/HSTcT3/DzXKr)

2. [American College of Emergency Physicians Clinical Policies Subcommittee (Writing Committee) on Acute Ischemic Stroke, Lo BM, Carpenter CR, et al. Clinical policy: Critical issues in the management of adult patients presenting to the emergency department with acute ischemic stroke. *Ann Emerg Med*. 2023;82(2):e17-e64.](http://paperpile.com/b/HSTcT3/wARye)

3. [American College of Emergency Physicians Clinical Policies Subcommittee (Writing Committee) on Thrombolytics, Lo BM, Carpenter CR, et al. Clinical policy: Use of thrombolytics for the management of acute ischemic stroke in the emergency department. *Ann Emerg Med*. 2024;84(6):e57-e86.](http://paperpile.com/b/HSTcT3/4x3Gv)

4. [Greenberg SM, Ziai WC, Cordonnier C, et al. 2022 guideline for the management of patients with spontaneous intracerebral hemorrhage: A guideline from the American heart association/American stroke association. *Stroke*. 2022;53(7):e282-e361.](http://paperpile.com/b/HSTcT3/e99XN)

5. [Redjal N, Venteicher AS, Dang D, et al. Guidelines in the management of CNS tumors. *J Neurooncol*. 2021;151(3):345-359.](http://paperpile.com/b/HSTcT3/ziU3U)

6. [Waefler N, Abid I, Montaut V, Donzé J, Zender H, John G. Neurological diagnostic tests for patients with and without delirium: a prospective observational study. *GeroScience*. 2024;46(6):6383-6393.](http://paperpile.com/b/HSTcT3/wGFEf)

7. [Lee S, Cavalier FR, Hayes JM, et al. Delirium, confusion, or altered mental status as a risk for abnormal head CT in older adults in the emergency department: A systematic review and meta-analysis. *Am J Emerg Med*. 2023;71:190-194.](http://paperpile.com/b/HSTcT3/DIHj4)

8. [Kennedy M, Hwang U, Han JH. Delirium in the emergency department: Moving from tool-based research to system-wide change. *J Am Geriatr Soc*. 2020;68(5):956-958.](http://paperpile.com/b/HSTcT3/KfuK0)

9. [Aslaner MA, Boz M, Çelik A, et al. Etiologies and delirium rates of elderly ED patients with acutely altered mental status: a multicenter prospective study. *Am J Emerg Med*. 2017;35(1):71-76.](http://paperpile.com/b/HSTcT3/fNWBf)

10. [Wilber ST, Ondrejka JE. Altered mental status and delirium. *Emerg Med Clin North Am*. 2016;34(3):649-665.](http://paperpile.com/b/HSTcT3/0JV3p)

11. [Lewis LM, Miller DK, Morley JE, Nork MJ, Lasater LC. Unrecognized delirium in ED geriatric patients. *Am J Emerg Med*. 1995;13(2):142-145.](http://paperpile.com/b/HSTcT3/htW5y)

12. [Hustey FM, Meldon SW. The prevalence and documentation of impaired mental status in elderly emergency department patients. *Ann Emerg Med*. 2002;39(3):248-253.](http://paperpile.com/b/HSTcT3/BJo18)

13. [Han JH, Schnelle JF, Ely EW. The relationship between a chief complaint of “altered mental status” and delirium in older emergency department patients. *Acad Emerg Med*. 2014;21(8):937-940.](http://paperpile.com/b/HSTcT3/t73KO)

14. [Carpenter CR, Lee S, Kennedy M, et al. Delirium detection in the emergency department: A diagnostic accuracy meta-analysis of history, physical examination, laboratory tests, and screening instruments. *Acad Emerg Med*. 2024;31(10):1014-1036.](http://paperpile.com/b/HSTcT3/Na7Mg)

15. [Liu SW, Lee S, Hayes JM, et al. Head computed tomography findings in geriatric emergency department patients with delirium, altered mental status, and confusion: A systematic review. *Acad Emerg Med*. 2023;30(6):616-625.](http://paperpile.com/b/HSTcT3/Vz1zp)

16. [Carpenter CR, Hammouda N, Linton EA, et al. Delirium prevention, detection, and treatment in emergency medicine settings: A Geriatric Emergency care Applied Research (GEAR) Network scoping review and consensus statement. *Acad Emerg Med*. 2021;28(1):19-35.](http://paperpile.com/b/HSTcT3/x0VSe)

17. [Hustey FM, Meldon SW, Smith MD, Lex CK. The effect of mental status screening on the care of elderly emergency department patients. *Ann Emerg Med*. 2003;41(5):678-684.](http://paperpile.com/b/HSTcT3/PcchM)

18. [Han JH, Zimmerman EE, Cutler N, et al. Delirium in older emergency department patients: recognition, risk factors, and psychomotor subtypes. *Acad Emerg Med*. 2009;16(3):193-200.](http://paperpile.com/b/HSTcT3/uFmmS)

19. [Pallin DJ, Allen MB, Espinola JA, Camargo CA Jr, Bohan JS. Population aging and emergency departments: visits will not increase, lengths-of-stay and hospitalizations will. *Health Aff (Millwood)*. 2013;32(7):1306-1312.](http://paperpile.com/b/HSTcT3/xTHax)

20. [Salvi F, Morichi V, Grilli A, Giorgi R, De Tommaso G, Dessì-Fulgheri P. The elderly in the emergency department: a critical review of problems and solutions. *Intern Emerg Med*. 2007;2(4):292-301.](http://paperpile.com/b/HSTcT3/JP9hA)

21. [van Loveren K, Singla A, Sinvani L, et al. Increased emergency department hallway length of stay is associated with development of delirium. *West J Emerg Med*. 2021;22(3):726-735.](http://paperpile.com/b/HSTcT3/4Bjq6)

22. [Moura Junior V, Westover MB, Li F, et al. Hospital complications among older adults: Better processes could reduce the risk of delirium. *Health Serv Manage Res*. 2022;35(3):154-163.](http://paperpile.com/b/HSTcT3/4ribb)

23. [Devlin JW, Skrobik Y, Gélinas C, et al. Executive summary: Clinical practice guidelines for the prevention and management of pain, agitation/sedation, delirium, immobility, and sleep disruption in adult patients in the ICU. *Crit Care Med*. 2018;46(9):1532-1548.](http://paperpile.com/b/HSTcT3/PTTZ3)

24. [Naughton BJ, Moran M, Ghaly Y, Michalakes C. Computed tomography scanning and delirium in elder patients. *Acad Emerg Med*. 1997;4(12):1107-1110.](http://paperpile.com/b/HSTcT3/2FkAJ)

25. [Tu LH, Venkatesh AK, Malhotra A, et al. Scenarios to improve CT head utilization in the emergency department delineated by critical results reporting. *Emerg Radiol*. 2022;29(1):81-88.](http://paperpile.com/b/HSTcT3/8F7PU)

26. [American College of Emergency Physicians, American Geriatrics Society, Emergency Nurses Association, Society for Academic Emergency Medicine, Geriatric Emergency Department Guidelines Task Force. Geriatric emergency department guidelines. *Ann Emerg Med*. 2014;63(5):e7-e25.](http://paperpile.com/b/HSTcT3/v7P4O)

27. [Lee S, Chen H, Hibino S, et al. Can we improve delirium prevention and treatment in the emergency department? A systematic review. *J Am Geriatr Soc*. 2022;70(6):1838-1849.](http://paperpile.com/b/HSTcT3/76KR4)

28. [American College of Radiology ACR Appropriateness Criteria® Altered Mental Status, Coma, Delirium, and Psychosis. Published online 2024.](http://paperpile.com/b/HSTcT3/VFYJi) <https://acsearch.acr.org/docs/3102409/Narrative/>

29. [Nesselroth D, Klang E, Soffer S, et al. Yield of head CT for acute findings in patients presenting to the emergency department. *Clin Imaging*. 2021;73:1-5.](http://paperpile.com/b/HSTcT3/B98Ne)

30. [Segard J, Montassier E, Trewick D, Le Conte P, Guillon B, Berrut G. Urgent computed tomography brain scan for elderly patients: can we improve its diagnostic yield? *Eur J Emerg Med*. 2013;20(1):51-53.](http://paperpile.com/b/HSTcT3/cb8Fu)

31. [Kohn MA, Carpenter CR, Newman TB. Understanding the direction of bias in studies of diagnostic test accuracy. *Acad Emerg Med*. 2013;20(11):1194-1206.](http://paperpile.com/b/HSTcT3/YCtNc)

32. [Harbison J, Hossain O, Jenkinson D, Davis J, Louw SJ, Ford GA. Diagnostic accuracy of stroke referrals from primary care, emergency room physicians, and ambulance staff using the face arm speech test. *Stroke*. 2003;34(1):71-76.](http://paperpile.com/b/HSTcT3/DUc2b)

33. [Jepsen JR, Laursen LH, Hagert CG, Kreiner S, Larsen AI. Diagnostic accuracy of the neurological upper limb examination I: inter-rater reproducibility of selected findings and patterns. *BMC Neurol*. 2006;6(1):8.](http://paperpile.com/b/HSTcT3/NOuvv)

34. [Jepsen JR, Laursen LH, Hagert CG, Kreiner S, Larsen AI. Diagnostic accuracy of the neurological upper limb examination II: relation to symptoms of patterns of findings. *BMC Neurol*. 2006;6(1):10.](http://paperpile.com/b/HSTcT3/Ipqo7)

35. [Sullivan SJ, Hammond-Tooke GD, Schneiders AG, Gray AR, McCrory P. The diagnostic accuracy of selected neurological tests. *J Clin Neurosci*. 2012;19(3):423-427.](http://paperpile.com/b/HSTcT3/t69AP)

36. [Easter JS, Haukoos JS, Meehan WP, Novack V, Edlow JA. Will neuroimaging reveal a severe intracranial injury in this adult with minor head trauma?: The rational clinical examination systematic review. *JAMA*. 2015;314(24):2672-2681.](http://paperpile.com/b/HSTcT3/6ODXF)

37. [Detsky ME, McDonald DR, Baerlocher MO, Tomlinson GA, McCrory DC, Booth CM. Does this patient with headache have a migraine or need neuroimaging? *JAMA*. 2006;296(10):1274-1283.](http://paperpile.com/b/HSTcT3/0ox7D)

38. [Nishijima DK, Gaona S, Waechter T, et al. Do EMS providers accurately ascertain anticoagulant and antiplatelet use in older adults with head trauma? *Prehosp Emerg Care*. 2017;21(2):209-215.](http://paperpile.com/b/HSTcT3/5hCHp)

39. [Selvanayagam N, Mowbray F, Clayton N, et al. Reliability of patient-reported outcome measures: Hemorrhage, anticoagulant, antiplatelet medication use. *Res Pract Thromb Haemost*. 2021;5(4):e12501.](http://paperpile.com/b/HSTcT3/eSGLE)

40. [Carpenter CR, Raja AS, Brown MD. Overtesting and the downstream consequences of overtreatment: Implications of “Preventing Overdiagnosis” for emergency medicine. *Acad Emerg Med*. 2015;22(12):1484-1492.](http://paperpile.com/b/HSTcT3/ND4dO)

41. [Lai MMY, Wong Tin Niam DM. Intracranial cause of delirium: computed tomography yield and predictive factors. *Intern Med J*. 2012;42(4):422-427.](http://paperpile.com/b/HSTcT3/FCOfH)

42. [Chary AN, Castilla-Ojo N, Joshi C, et al. Evaluating older adults with cognitive dysfunction: A qualitative study with emergency clinicians. *J Am Geriatr Soc*. 2022;70(2):341-351.](http://paperpile.com/b/HSTcT3/KBCfI)

43. [Suffoletto B, Miller T, Frisch A, Callaway C. Emergency physician recognition of delirium. *Postgrad Med J*. 2013;89(1057):621-625.](http://paperpile.com/b/HSTcT3/ezCRr)

44. [Sands MB, Sharma S, Carpenter L, et al. “SQiD, the Single Question in Delirium; can a single question help clinicians to detect delirium in hospitalised cancer patients?” running heading Single Question in Delirium" (Bcan-D-20-01665). *BMC Cancer*. 2021;21(1):75.](http://paperpile.com/b/HSTcT3/vxYKd)

45. [McCleary E, Cumming P. Improving early recognition of delirium using SQiD (Single Question to identify Delirium): a hospital based quality improvement project. *BMJ Qual Improv Rep*. 2015;4(1):u206598.w2653.](http://paperpile.com/b/HSTcT3/uZRbQ)

46. [Amore M, D’Andrea M, Fagiolini A. Treatment of agitation with lorazepam in clinical practice: A systematic review. *Front Psychiatry*. 2021;12:628965.](http://paperpile.com/b/HSTcT3/KZ4QN)

47. [Oliveira J E Silva L, Berning MJ, Stanich JA, et al. Risk factors for delirium in older adults in the emergency department: A systematic review and meta-analysis. *Ann Emerg Med*. 2021;78(4):549-565.](http://paperpile.com/b/HSTcT3/0uk2t)

48. [Goldberg A, Straus SE, Hamid JS, Wong CL. Room transfers and the risk of delirium incidence amongst hospitalized elderly medical patients: a case-control study. *BMC Geriatr*. 2015;15(1):69.](http://paperpile.com/b/HSTcT3/xwAvz)

49. [Kane B, Carpenter CR. Cognition and Decision Making. In: *Washington Manual of Patient Safety and Quality Improvement (Lippincott Manual Series)*. Lippincott Williams and Wilkins; 2016:195-209.](http://paperpile.com/b/HSTcT3/gRv2O)

50. [Thompson RJ, Wojcik SM, Grant WD, Ko PY. Incidental findings on CT scans in the emergency department. *Emerg Med Int*. 2011;2011:624847.](http://paperpile.com/b/HSTcT3/zdEyt)

51. [Akhtar H, Chaudhry SH, Bortolussi-Courval É, et al. Diagnostic yield of CT head in delirium and altered mental status-A systematic review and meta-analysis. *J Am Geriatr Soc*. 2023;71(3):946-958.](http://paperpile.com/b/HSTcT3/Nqsj2)

52. [Leong LB, Jian KHW, Vasu A, Seow E. Prospective study of patients with altered mental status: clinical features and outcome. *Int J Emerg Med*. 2008;1(3):179-182.](http://paperpile.com/b/HSTcT3/bSuml)

53. [Hasan LM, Khan A, Singh M, Malone ML. Brain imaging in older patients with delirium. *J Patient Cent Res Rev*. 2017;4(4):261.](http://paperpile.com/b/HSTcT3/yYda7)

54. [Thomson J, Peter Wayne New. Pharmacological management of stroke in older people. *J Pharm Pr Res*. 2021;51(1):67-77.](http://paperpile.com/b/HSTcT3/XHTwj)

55. [Sahni R, Weinberger J. Management of intracerebral hemorrhage. *Vasc Health Risk Manag*. 2007;3(5):701-709.](http://paperpile.com/b/HSTcT3/i3Po6)

56. [Nayak L, Iwamoto FM. Primary brain tumors in the elderly. *Curr Neurol Neurosci Rep*. 2010;10(4):252-258.](http://paperpile.com/b/HSTcT3/pIJaL)

57. [Theisen-Toupal J, Breu AC, Mattison MLP, Arnaout R. Diagnostic yield of head computed tomography for the hospitalized medical patient with delirium. *J Hosp Med*. 2014;9(8):497-501.](http://paperpile.com/b/HSTcT3/3LP8q)

58. [Prevedello LM, Raja AS, Zane RD, et al. Variation in use of head computed tomography by emergency physicians. *Am J Med*. 2012;125(4):356-364.](http://paperpile.com/b/HSTcT3/mdbRA)

59. [Wang X, You JJ. Head CT for nontrauma patients in the emergency department: clinical predictors of abnormal findings. *Radiology*. 2013;266(3):783-790.](http://paperpile.com/b/HSTcT3/pgOaN)

60. [Larson DB, Johnson LW, Schnell BM, Salisbury SR, Forman HP. National trends in CT use in the emergency department: 1995-2007. *Radiology*. 2011;258(1):164-173.](http://paperpile.com/b/HSTcT3/qO6Gj)

61. [Emberson J, Lees KR, Lyden P, et al. Effect of treatment delay, age, and stroke severity on the effects of intravenous thrombolysis with alteplase for acute ischaemic stroke: a meta-analysis of individual patient data from randomised trials. *Lancet*. 2014;384(9958):1929-1935.](http://paperpile.com/b/HSTcT3/f7cB7)

62. [Benko MJ, Abdulla SG, Cuoco JA, et al. Short- and long-term geriatric mortality after acute traumatic subdural hemorrhage. *World Neurosurg*. 2019;130:e350-e355.](http://paperpile.com/b/HSTcT3/pwsyl)

63. [Millet I, Sebbane M, Molinari N, et al. Systematic unenhanced CT for acute abdominal symptoms in the elderly patients improves both emergency department diagnosis and prompt clinical management. *Eur Radiol*. 2017;27(2):868-877.](http://paperpile.com/b/HSTcT3/5s7KR)

64. [Rodriguez RM, Henderson TM, Ritchie AM, et al. Patient preferences and acceptable risk for computed tomography in trauma. *Injury*. 2014;45(9):1345-1349.](http://paperpile.com/b/HSTcT3/TUIiK)

65. [Gimbel RW, Pirrallo RG, Lowe SC, et al. Effect of clinical decision rules, patient cost and malpractice information on clinician brain CT image ordering: a randomized controlled trial. *BMC Med Inform Decis Mak*. 2018;18(1):20.](http://paperpile.com/b/HSTcT3/R2UPw)

66. [Hess EP, Grudzen CR, Thomson R, Raja AS, Carpenter CR. Shared Decision-making in the Emergency Department: Respecting Patient autonomy when seconds count. *Acad Emerg Med*. 2015;22(7):856-864.](http://paperpile.com/b/HSTcT3/V2pGg)

67. [Kanzaria HK, McCabe AM, Meisel ZM, et al. Advancing patient-centered outcomes in emergency Diagnostic Imaging: A Research Agenda. *Acad Emerg Med*. 2015;22(12):1435-1446.](http://paperpile.com/b/HSTcT3/voBWP)

68. [Hogan TM, Richmond NL, Carpenter CR, et al. Shared decision making to improve the emergency care of older adults: A research agenda. *Acad Emerg Med*. 2016;23(12):1386-1393.](http://paperpile.com/b/HSTcT3/qfazI)

69. [Fatovich DM. The inverted U curve and emergency medicine: Overdiagnosis and the law of unintended consequences. *Emerg Med Australas*. 2016;28(4):480-482.](http://paperpile.com/b/HSTcT3/439XY)

70. [Han JH, Wilson A, Graves AJ, et al. Validation of the Confusion Assessment Method for the Intensive Care Unit in older emergency department patients. *Acad Emerg Med*. 2014;21(2):180-187.](http://paperpile.com/b/HSTcT3/5WZaw)

71. [Stein SC, Burnett MG, Glick HA. Indications for CT scanning in mild traumatic brain injury: A cost-effectiveness study. *J Trauma*. 2006;61(3):558-566.](http://paperpile.com/b/HSTcT3/Xv1zS)

72. [Jordan YJ, Lightfoote JB, Jordan JE. Computed tomography imaging in the management of headache in the emergency department: cost efficacy and policy implications. *J Natl Med Assoc*. 2009;101(4):331-335.](http://paperpile.com/b/HSTcT3/ix2oE)

73. [Janke AT, Melnick ER, Venkatesh AK. Hospital occupancy and emergency department boarding during the COVID-19 pandemic. *JAMA Netw Open*. 2022;5(9):e2233964.](http://paperpile.com/b/HSTcT3/QRW2T)

74. [Kanzaria HK, Probst MA, Ponce NA, Hsia RY. The association between advanced diagnostic imaging and ED length of stay. *Am J Emerg Med*. 2014;32(10):1253-1258.](http://paperpile.com/b/HSTcT3/vWagJ)

75. [Boudi Z, Lauque D, Alsabri M, et al. Association between boarding in the emergency department and in-hospital mortality: A systematic review. *PLoS One*. 2020;15(4):e0231253.](http://paperpile.com/b/HSTcT3/DFEi2)

76. [Ng P, McGowan M, Goldstein M, Kassardjian CD, Steinhart BD. The impact of computed tomography head scans on emergency department management and length of stay in bizarre behavior patients. *Am J Emerg Med*. 2017;36(2):213-217.](http://paperpile.com/b/HSTcT3/PU9Uq)

77. [Obara S, Nakata Y, Yamaoka K. Cost-effectiveness analysis of sedation and general anesthesia regimens for children undergoing magnetic resonance imaging in Japan. *J Anesth*. 2022;36(3):359-366.](http://paperpile.com/b/HSTcT3/MWnbw)

78. [Gawor G, Biese K, Platts-Mills TF. Delay in spinal cord injury diagnosis due to sedation: a case report. *J Emerg Med*. 2012;43(6):e413-e418.](http://paperpile.com/b/HSTcT3/4uBko)

79. [Arnold MR, Cunningham KW, Atkins TG, et al. Redefining Mild Traumatic Brain Injury (mTBI) delineates cost effective triage. *Am J Emerg Med*. 2020;38(6):1097-1101.](http://paperpile.com/b/HSTcT3/tETIG)

80. [Ward MM, Carter KD, Ullrich F, et al. Averted transfers in rural emergency departments using telemedicine: Rates and costs across six networks. *Telemed J E Health*. 2021;27(5):481-487.](http://paperpile.com/b/HSTcT3/Wtdd7)

81. [Newman-Toker DE, Moy E, Valente E, Coffey R, Hines AL. Missed diagnosis of stroke in the emergency department: a cross-sectional analysis of a large population-based sample. *Diagnosis (Berl)*. 2014;1(2):155-166.](http://paperpile.com/b/HSTcT3/hfzFr)

82. [Ross AB, Kalia V, Chan BY, Li G. The influence of patient race on the use of diagnostic imaging in United States emergency departments: data from the National Hospital Ambulatory Medical Care survey. *BMC Health Serv Res*. 2020;20(1):840.](http://paperpile.com/b/HSTcT3/ETImF)

83. [Chary AN, Torres B, Brickhouse E, et al. Language discordance in emergency department delirium screening: Results from a qualitative interview-based study. *J Am Geriatr Soc*. 2023;71(4):1328-1331.](http://paperpile.com/b/HSTcT3/a9IXg)

84. [Chary AN, Suh M, Ordoñez E, et al. A scoping review of geriatric emergency medicine research transparency in diversity, equity, and inclusion reporting. *J Am Geriatr Soc*. 2024;72(11):3551-3566.](http://paperpile.com/b/HSTcT3/HGSi9)

85. [Billing and Coding: MRI and CT Scans of the Head and Neck. 2019. Accessed December 18, 2024.](http://paperpile.com/b/HSTcT3/uuHaB) <https://www.cms.gov/medicare-coverage-database/view/article.aspx?articleId=57215>

86. [Ginde AA, Foianini A, Renner DM, Valley M, Camargo CA Jr. Availability and quality of computed tomography and magnetic resonance imaging equipment in U.S. emergency departments. *Acad Emerg Med*. 2008;15(8):780-783.](http://paperpile.com/b/HSTcT3/rYdg0)

87. [Bergeron C, Fleet R, Tounkara FK, Lavallée-Bourget I, Turgeon-Pelchat C. Lack of CT scanner in a rural emergency department increases inter-facility transfers: a pilot study. *BMC Res Notes*. 2017;10(1):772.](http://paperpile.com/b/HSTcT3/mkVjb)

88. [Chary AN, Lesser A, Inouye SK, Carpenter CR, Stuck AR, Kennedy M. A survey of delirium self-reported knowledge and practices among emergency physicians in the United States. *J Geriatr Emerg Med*. 2021;2(12). doi:](http://paperpile.com/b/HSTcT3/lJnTD)[10.17294/2694-4715.1010](http://dx.doi.org/10.17294/2694-4715.1010)

89. [Chary A, Liu SW, Santangelo I, et al. A qualitative study of emergency department delirium prevention initiatives. *Delirium Commun*. 2022;2022. doi:](http://paperpile.com/b/HSTcT3/LoFfQ)[10.56392/001c.55690](http://dx.doi.org/10.56392/001c.55690)

90. [Chary A, Joshi C, Castilla-Ojo N, et al. Emergency clinicians’ perceptions of communication tools to establish the mental baseline of older adults: A Qualitative study. *Cureus*. 2021;13(12):e20616.](http://paperpile.com/b/HSTcT3/cktJu)

91. [Chary AN, Bhananker AR, Brickhouse E, et al. Implementation of delirium screening in the emergency department: A qualitative study with early adopters. *J Am Geriatr Soc*. 2024;72(12):3753-3762.](http://paperpile.com/b/HSTcT3/FWqKx)

92. [Shih RD, Carpenter CR, Tolia V, Binder EF, Ouslander JG. Balancing vision with pragmatism: The geriatric emergency department guidelines-realistic expectations from emergency medicine and geriatric medicine. *J Am Geriatr Soc*. 2022;70(5):1368-1373.](http://paperpile.com/b/HSTcT3/hXxtt)

93. [Diner BM, Carpenter CR, O’Connell T, et al. Graduate medical education and knowledge translation: role models, information pipelines, and practice change thresholds. *Acad Emerg Med*. 2007;14(11):1008-1014.](http://paperpile.com/b/HSTcT3/VxQOP)

**Supplement 4: AGREE-II assessment tool for a trustworthy clinical practice guideline**


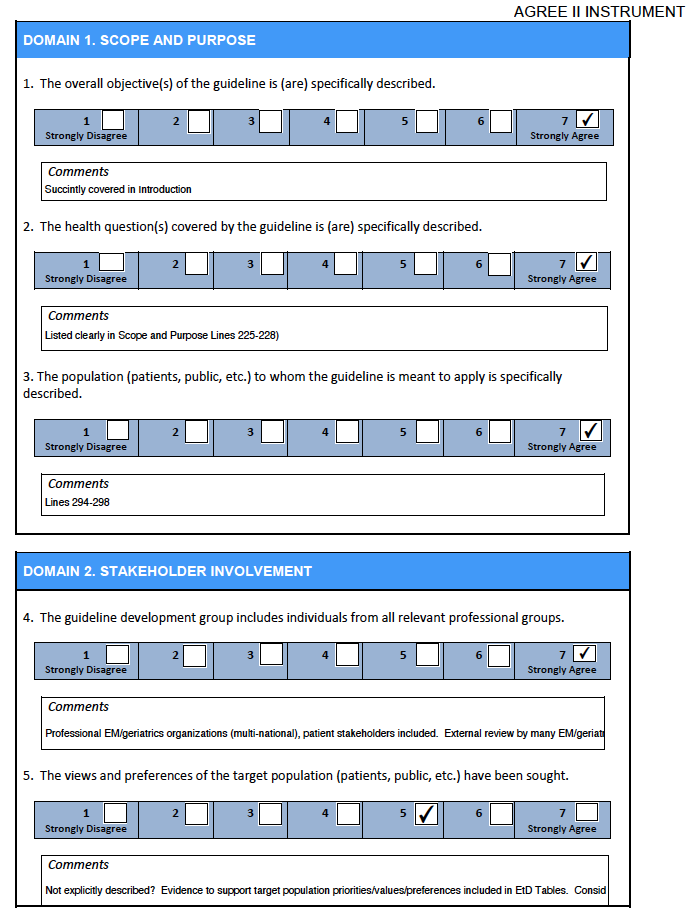


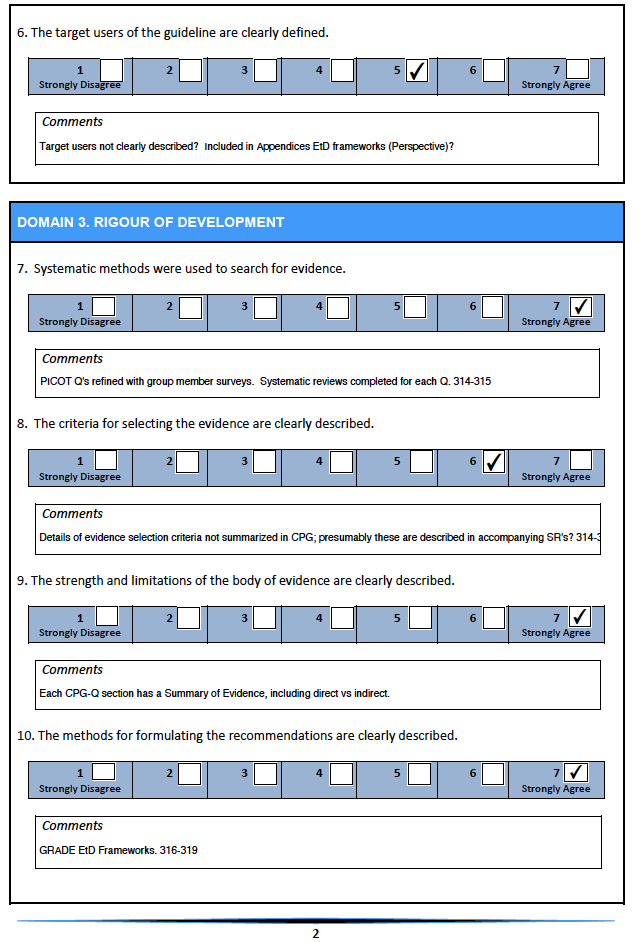


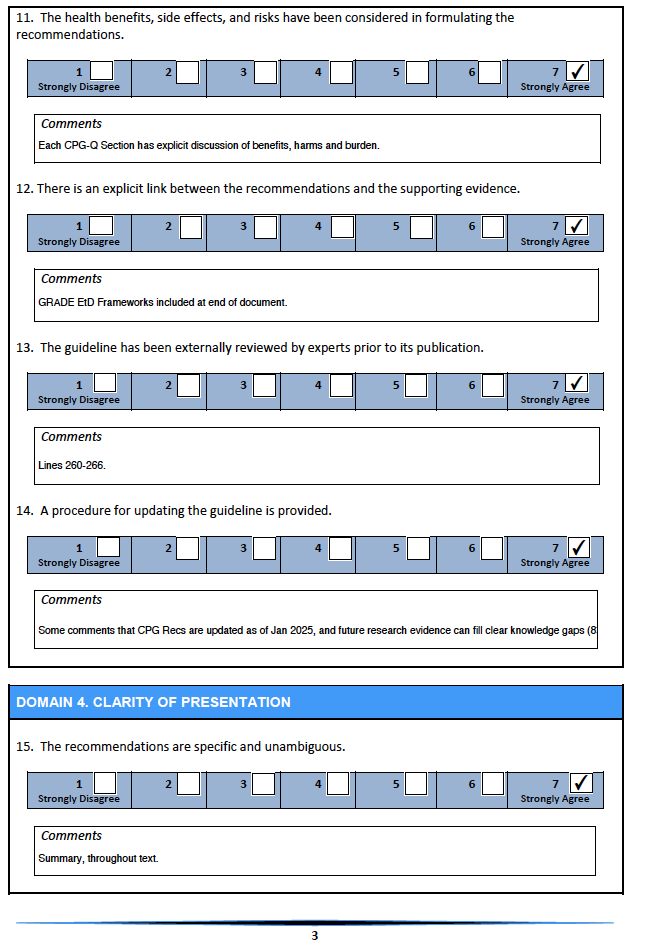


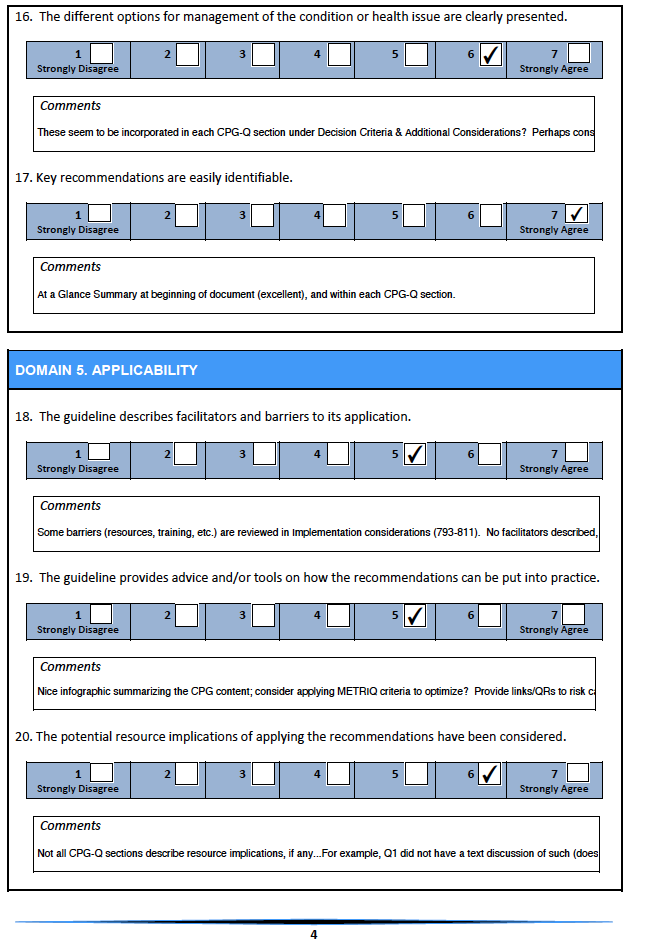


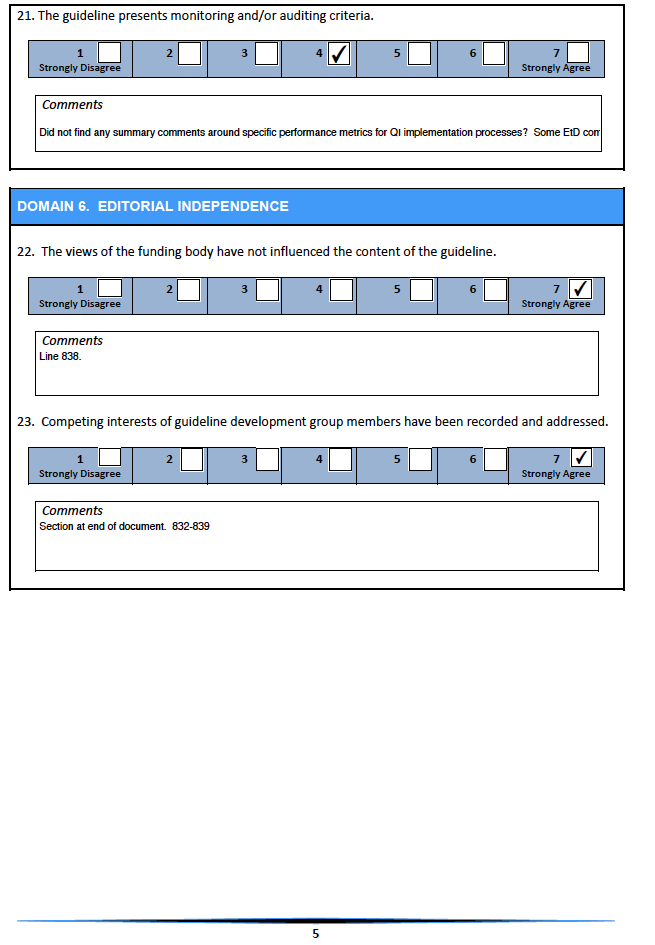


**Supplement 5: Large Group Voting results on selection of PICO questions**
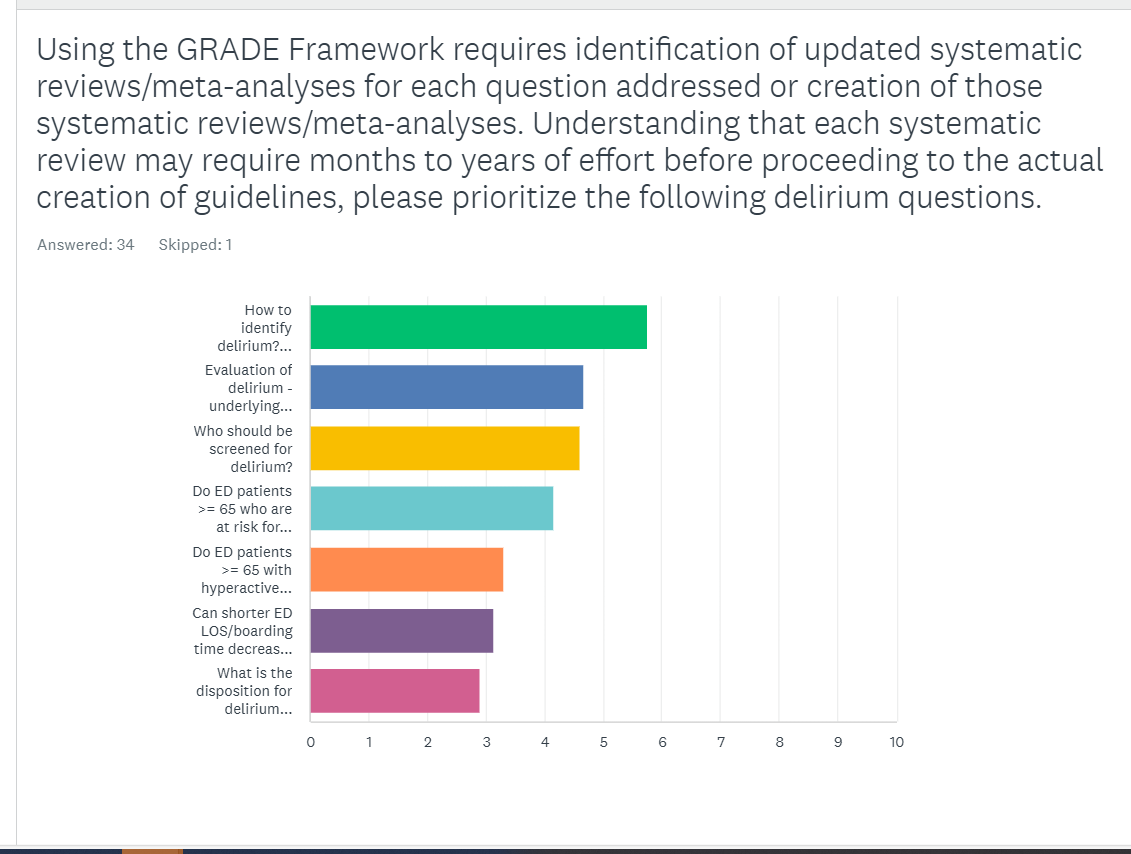

Supplement: Supplementary file 1 — Appendix S1: PICO 1 Evidence to Decision document. Appendix S2: PICO2 Evidence to Decision document. Appendix S3: PICO3 Evidence to Decision document. [file ACEM-33-0-s001.docx]
